# Atom Economical QPCs: Phenyl-Free Biscationic Quaternary Phosphonium Compounds as Potent Disinfectants

**DOI:** 10.1021/acsinfecdis.2c00575

**Published:** 2023-02-09

**Authors:** Laura M. Thierer, Ashley A. Petersen, Marina E. Michaud, Christian A. Sanchez, Samantha R. Brayton, William M. Wuest, Kevin P. C. Minbiole

**Affiliations:** † Department of Chemistry, Villanova University, Villanova, Pennsylvania 19085, United States of America; ‡ Department of Chemistry, Emory University, Atlanta, Georgia 30322, United States of America

**Keywords:** Amphiphiles, Disinfectants, Quaternary Phosphonium Compounds, QPC

## Abstract

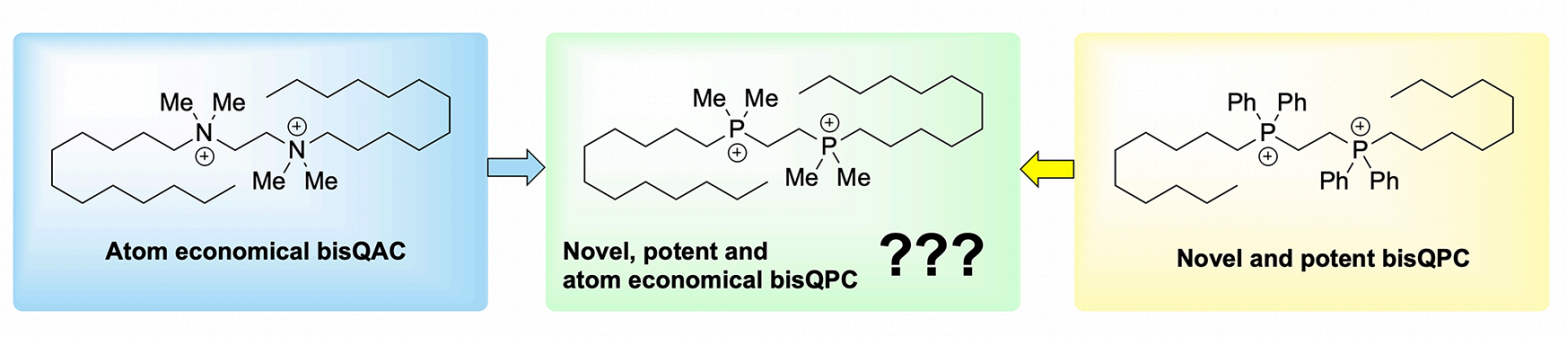

Quaternary ammonium compounds (QACs) are vital disinfectants for the neutralization of pathogenic bacteria in clinical, domestic, and commercial settings. After decades of dependence on QACs, the emergence of antimicrobial resistance to this class of compounds threatens the ability of existing QAC products to effectively manage rising bacterial threats. The need for new disinfectants is therefore urgent, with quaternary phosphonium compounds (QPCs) emerging as a new class of promising antimicrobials that boast significant activity against highly resistant bacteria. Reported here is a series of twenty-one novel QPCs that replace phenyl substituents on the phosphorus center with alkyl groups yet allow for rapid synthetic routes in high yields. Within this series are structures containing methyl, ethyl, or cyclohexyl phosphonium substituents on bisphosphane scaffolds bearing ethyl linkers, affording atom economical structures and ones that represent exact analogs to nitrogenous amphiphiles. The resultant bisQPC structures display high antibacterial efficacy enjoyed by comparably constructed QACs, with three structures in the single-digit micromolar activity range despite structural simplification.

For approximately 100 years, quaternary ammonium compounds (QACs) have been employed as successful tools to mitigate the transfer of pathogenic bacteria.^1,2^ Over much of this time, society has heavily relied on just a handful of these disinfecting amphiphilic compounds, with the most visible commercial disinfectant of this class being benzalkonium chloride (BAC), a mixture of long-chained alkyl dimethylbenzylammonium chlorides.^2−4^ While mainstay disinfectants such as BAC have proven highly valuable for decades, they are now showing vulnerabilities as bacteria continue to evolve antimicrobial resistance mechanisms.^5^ Notable pathogens such as *A. baumannii* and *P. aeruginosa* have developed sufficient resistance mechanisms toward common disinfectants that they are renewing the problem of bacterial infections in hospital, domestic, and commercial settings.^6−10^ In order to combat the growing problem of antimicrobial resistance (AMR), ongoing efforts toward the synthesis and antimicrobial evaluation of novel QACs are underway.^11^ Along these lines, our groups have systematically evaluated a variety of QAC structural motifs to identify molecular features that can inform the rational design of new classes of disinfectants; we have recently advanced to machine learning techniques to predict the bioactivity of proposed compounds.^12^ Despite the development of over 700 different compounds by our groups in the past decade, concerns remain about the cross-resistance in AMR mechanisms^13^ between established QACs such as BAC into any novel QAC.^3,10^ Accordingly, exploration of molecules with markedly different cationic amphiphilic heads, such as quaternary phosphonium compounds (QPCs)^14−19^ and trivalent sulfonium compounds (TSCs),^20−22^ are being pursued. Investigation into alternative amphiphiles resulted in the discovery of highly effective novel biscationic QPC compounds, highlighted by a structure termed P6P-10,10; for clarity within this paper, it will be labeled hereafter as ^Ph^P6P-10,10 (Figure 1).^15^ This structure took advantage of the ready availability and stability of phenyl-substituted bisphosphines, which are often employed as ligands for catalysis; presumably, the phenyl substituents stabilize the phosphines and inhibit oxidation and other reactivity. The high degree of antimicrobial efficacy observed with ^Ph^P6P-10,10 toward both Gram-positive and Gram-negative pathogens, including highly resistant pathogenic bacteria, was unprecedented when compared with 14 antibiotics and 15 other disinfectant amphiphiles.^10,15^

**Figure 1:**
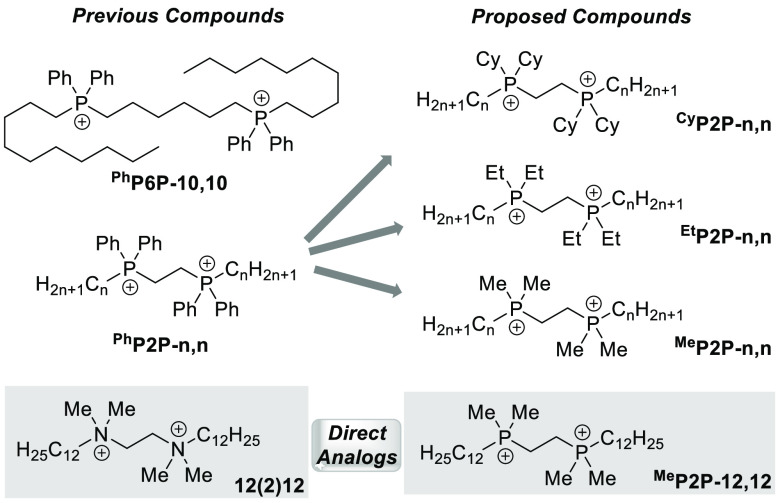
Prior examples of bisQAC and newly synthesized bisQPCs. All compounds have bromide counterions (not shown).

Given the promise of this early generation of bisQPC disinfectants, new alternatives are desired to expand this emerging class of amphiphiles. We envisioned an opportunity to accomplish two parallel goals, aiming (1) to determine if the bulky phenyl groups could be replaced with smaller alkyl groups, rendering the disinfectant structures more atom economical, and (2) to prepare exact nitrogen- and phosphorus-bearing analogs in amphiphilic disinfectants, thus scrutinizing the role of the cationic element in the antimicrobial action. We drew inspiration from the highly effective tetramethylethylenediamine-derived bisQACs prepared in our laboratories, specifically 12(2)12, and prior work in comparing analogous monoQAC and monoQPC compounds.^19,23^ We thus sought to replace the phenyl rings on the ^Ph^P2P-n,n structure (Figure 1, left) with alkyl substituents (Figure 1, right), leading to series dubbed ^Cy^P2P-n,n, ^Et^P2P-n,n, and ^Me^P2P-n,n for cyclohexyl, ethyl, and methyl analogs, respectively. Cyclohexyl-substituted QPCs would serve to inform the transition from aromatic substituents in the first generation of bisQPCs to alkyl substituents, presumably providing steric protection from air oxidation for the phosphorus atom. Preparation of methyl- and ethyl-substituted bisQPC analogs would serve to maximize atom economy within this structural motif and identify minimum structural requirements for strong bioactivity. Further, the methyl-substituted phosphonium compounds would allow for direct comparison of bisQPC to bisQAC activity (Figure 1 bottom, shaded compounds), since the only change in the molecules would be the pnictogen atom (nitrogen or phosphorus) of the cationic headgroup.

## Results and discussion

### Synthesis of Alkyl bisQPCs

Prior work from our groups^14,15,24^ and others^25^ has indicated that amphiphilic QAC and QPC compounds containing alkyl chain(s) of 10 to 12 carbon atoms generally display optimal efficacy as disinfectants. We therefore targeted the preparation of a series of compounds with alkyl chain lengths between 8 and 16 carbons to bracket this hypothesized ideal nonpolar region.

**Scheme 1:**
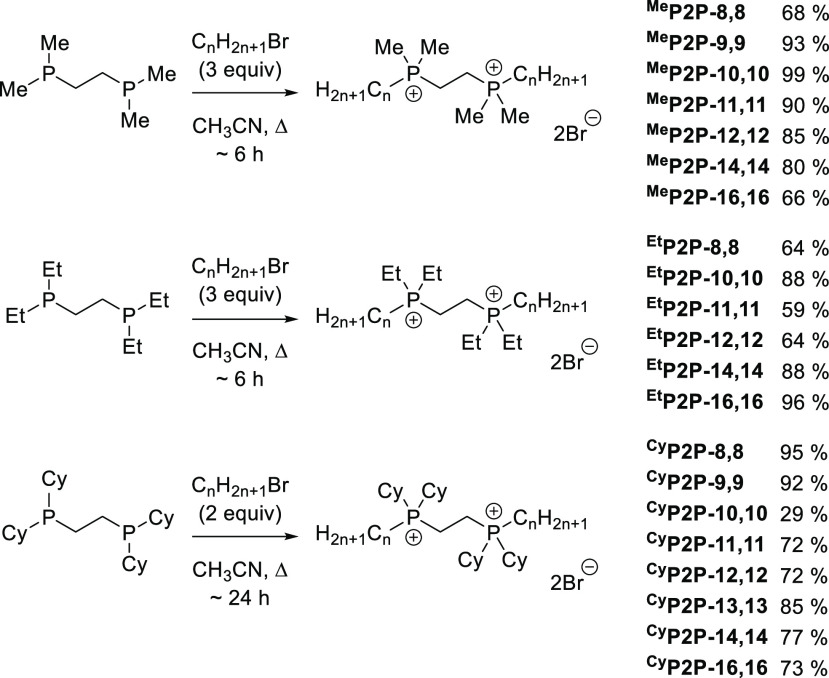
Preparation of Alkyl Substituted bisQPCs

Preparation of the alkyl substituted bisQPCs was readily accomplished through the treatment of the relevant bisphosphinoethane with excess equivalents of a linear chain alkyl bromide in acetonitrile at elevated temperatures (Scheme 1). To reduce the risk of oxidizing the alkyl phosphine, reactions were conducted under an atmosphere of argon in pressure-rated reaction vials with rupture disk septum caps. BisQPC products were generally isolated in 60–95% yields, with the primary source of variation in yields stemming from the process of handling and purifying waxy or glassy products. Detailed reaction procedures as well as characterization of the prepared compounds by ^1^H, ^13^C, and ^31^P NMR and HRMS are available in the Supporting Information.

The sterically hindered 1,2(bisdicyclohexylphosphonium)ethane was slow to oxidize under ambient atmospheric conditions allowing for quick handling of the solid on the bench. Subsequent exclusion of air from the reaction vial via a 15 min argon flush following the addition of the phosphine created a suitable reaction environment. The more reactive 1,2(bisdiethylphosphonium)ethane and 1,2(bisdimethylphosphonium)ethane were significantly more air sensitive and therefore were loaded to argon-flushed reaction vials via syringe through a septum cap using standard Schlenk line techniques. The quantity of acetonitrile used as a solvent for all reactions was minimized to reduce adventitious water. These measures effectively prevented the formation of phosphine oxide compounds, as evidenced by the observation of little to no oxidized product in ^31^P NMR spectra, even in crude product mixtures.

The reaction times required to reach conversion correlated to the expected influence of steric hindrance around the phosphine. The less sterically constrained methyl- and ethyl-substituted phosphines quickly increased viscosity upon heating, and the reactions were complete within 5–6 h, while the phosphines with the much larger cyclohexyl groups generally required heating for 24 h to ensure complete alkylation. It is notable that, while reactions with alkyl bromides were uniformly successful, the use of alkyl chloride electrophiles led to incomplete reactions and, ultimately, significant phosphorus oxidation in our hands.

### Characterization Data

**Figure 2:**
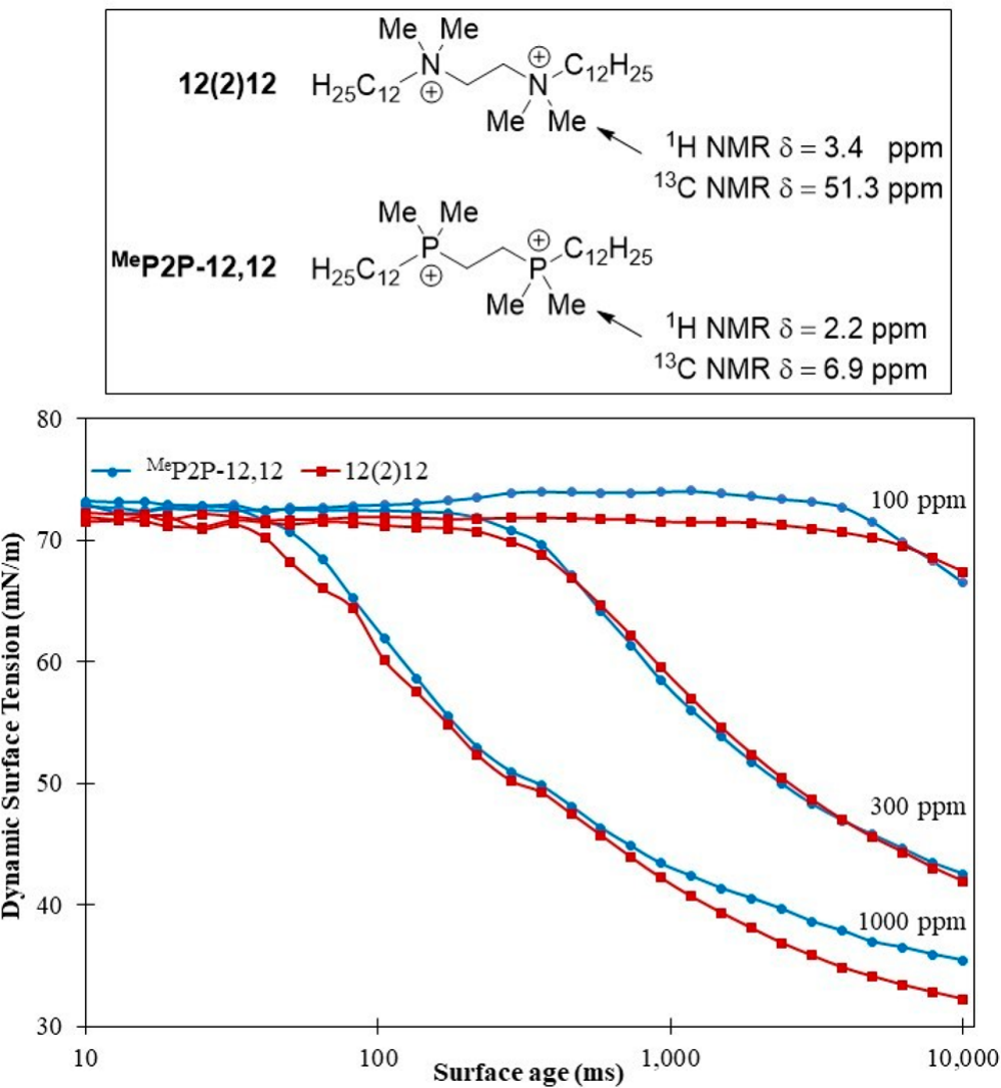
Characterization data for structurally analogous bisQAC and bisQPC compounds 12(2)12 and ^Me^P2P-12,12. Shown are the NMR chemical shifts (top) and dynamic surface tension (mN/m) data when plotted as a function of surface age (ms) at concentrations of 100, 300, and 1000 ppm (bottom). Bromide counterions omitted for clarity.

In comparing the spectroscopic data of ^Me^P2P-12,12 to its bisQAC analog 12(2)12, significant differentiation was noted. Both the ^1^H NMR and ^13^C NMR data demonstrate a marked upfield shift in the signals for atoms neighboring the phosphonium cation, as compared to the ammonium cation (Figure 2, top). Comparison of the methyl substituents was particularly revealing, as chemical shifts for the ^1^H NMR signals assigned to the methyl substituents on the phosphonium cation were 2.2 ppm compared to the corresponding methyl groups on the ammonium cation of 3.4 ppm, indicating a more electron-rich environment on the methyl substituents adjacent to the phosphonium cation. Kanazawa and co-workers also reported P-CH_3_^1^H NMR signals near 2 ppm in monoQPCs.^19^ An ^1^H–^13^C HSQC NMR experiment was subsequently conducted on each of these compounds to unambiguously identify the ^13^C NMR signal associated with the methyl group within each compound (see Figures S64 and S65 in the SI). From this analysis, the methyl carbons again revealed a marked change, from a chemical shift of 51.3 ppm in 12(2)12 to a significantly more shielded 6.9 ppm for ^Me^P2P-12,12. Chen and co-workers reported similar spectra in ionic liquid investigations; chemical shift differences were rationalized by electronegativity differences of nitrogen versus phosphorus when bonded to carbon atoms at the amphiphilic headgroup.^26^ These data highlight the markedly different electronic environments and cationic distribution in these pnictogen analogs.

In order to further investigate these highly analogous bis-cationic structures, a Krüss bubble pressure tensiometer was utilized to measure the dynamic surface tension of 12(2)12 and ^Me^P2P-12,12. Antimicrobial activity is expected to be affected by differences in solution behavior of amphiphilic molecules, such as the propensity to form micelles and solution dispersion rates.^27^ Structural factors such as the nature of the charge and the alkyl chain length of the amphiphile have been previously reported to change the behavior of surfactants.^27−30^ These types of structural factors are consistent with the factors that influence our proposed mechanism of action for bacteria membrane disruption by multicationic QACs.^31^ We, therefore, were interested in whether the identity of the cationic species in these amphiphiles would change dynamic surface tension as a physical property that could be correlated to observed bioactivity data. The dynamic surface tension experiments were completed at three different concentrations (100, 300, and 1000 ppm) to elucidate any concentration dependence upon the data. When comparing the dynamic surface tension of 12(2)12 and ^Me^P2P-12,12, it is evident that these compounds behave almost identically in solution, despite the change of cationic center (Figure 2, bottom). This indicates that the similarity in structure may be the prevailing factor in antimicrobial behavior over the nature of the cationic head itself for these analogous compounds.

### Biological Activity

The prepared library of biscationic amphiphilic compounds was assessed for performance as antibacterial disinfectants by determining the minimum inhibitory concentration (MIC) for each against a panel of bacteria. The bacteria selected for the study were chosen to evaluate the broad spectrum antimicrobial performance of the QPCs and, therefore, included both Gram-positive and Gram-negative bacterial strains, including those with known antimicrobial resistance mechanisms.^4^ These bacteria include the Gram-positive strains of methicillin-susceptible *Staphylococcus aureus* [MSSA; SH1000], community-acquired methicillin-resistant *Staphylococcus aureus* [CA-MRSA; USA 300-0114], and hospital-acquired methicillin-resistant *Staphylococcus aureus* [HA-MRSA; ATCC 33591] and the Gram-negative strains of *Enterococcus faecalis* [OG1RF], *Escherichia coli* [MC4100], *Acinetobacter baumannii* [ATCC 17498], and *Pseudomonas aeruginosa* [PAO1]. The potential cytotoxicity of the compounds was also considered with the evaluation of hemolysis (lysis20) assays with 20% lysis of red blood cells serving as an indicator of potentially toxic compounds. Finally, the bioactivity of these compounds was benchmarked against a commercial benzalkonium chloride source (BAC; 70% benzyldimethyldodecylammonium chloride, and 30% benzyldimethyltetradecylammonium chloride) as well as some previously reported disinfectant compounds from our groups; included were our current best-in-class bisQPC ^Ph^P6P-10,10 as well as ^Ph^P2P-10,10 and 12(2)12, in light of the aforementioned similarity of structural motifs to these ^R^P2P-n,n compounds.^15,24^ A detailed description of the experimental procedures used to evaluate bioactivity and hemolysis assays can be found in the Methods section, with the resulting data reported in Table 1.

**Table 1:**
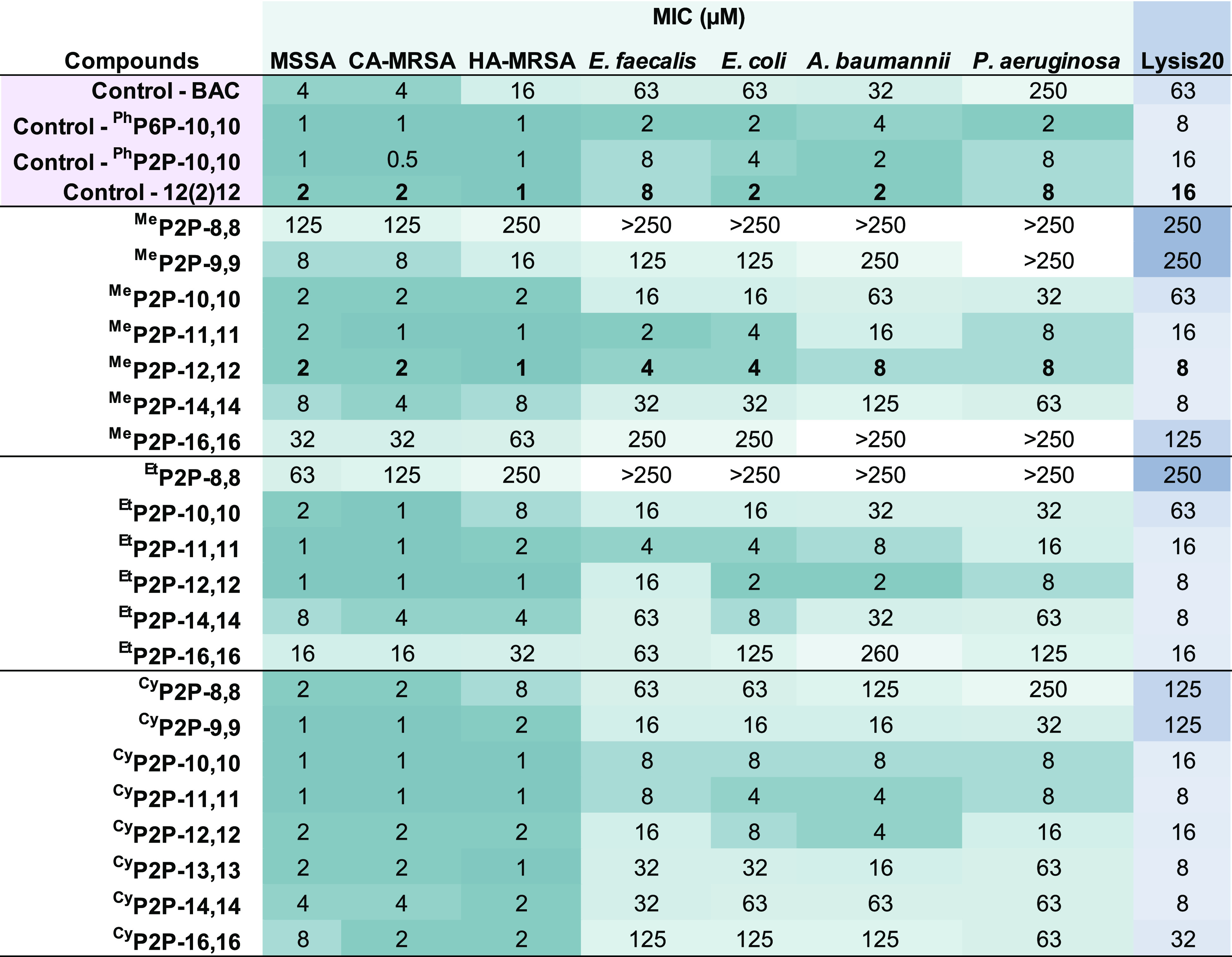
Biological Activity and Lysis20 Data of Alkyl bisQPC Compounds^a^

a Darker colors indicate preferential performance. Bolded results highlight the structurally analogous bisQPC MeP2P-12,12 and bisQAC 12(2)12.

While our physical characterization of the bisQPC structures demonstrated some similarities and differences in comparison to the nitrogenous bisQAC analogs, we were most interested in biological activity assessments, particularly in comparison to phenyl-containing analogs. In line with expectations, the most visible bioactivity trend indicated that the highest antimicrobial activity was observed for the structures with alkyl chain lengths of 10–12 carbons. Performance progressively diminished as chain lengths either increased to 16 carbons (compromising solubility) or decreased to eight carbons (reducing amphiphilicity). The most effective disinfectants within this series were observed to be ^Cy^P2P-11,11, ^Et^P2P-12,12, and ^Me^P2P-12,12; all display single-digit micromolar activity with just one exception in the ^Et^P2P-12,12 panel. This correlation between chain length and bioactivity has been repeatedly observed by our groups as well as others.^14,15,24,25^ Among these three lead compounds, ^Cy^P2P-11,11 has a slight overall advantage in broad-spectrum potency within this data set, with MIC values of 1–8 μM. All three of these top compounds clearly out-perform the commercial BAC control but do not reach the level of performance observed with our current best-in-class QPC, ^Ph^P6P-10,10, with MIC values of 1–4 μM. Notably, the top-performing compounds still maintained high efficacy against the Gram-negative pathogens, which bear known disinfectant resistance mechanisms, such as the expression of multidrug efflux pumps with cross-resistance to QACs.^10,32,33^ Additionally, relative to BAC, improved activity was observed against the Gram-negative pathogens HA-MRSA and *E. faecalis*, also known to harbor multidrug efflux pumps that decrease their susceptibility to disinfectants.^15,32,33^ Together, these potent bioactivities against prominent refractory Gram-positive and Gram-negative pathogens underscore the promise of QPCs as a nascent, structurally diverse disinfectant class that can evade rapidly spreading QAC resistance.^34^

In comparison of this set to structurally analogous phenyl-substituted ^Ph^P2P-10,10, the bioactivities of these top three compounds approach parity. Interestingly, an additive effect seems to be present between the alkyl chain length (*n*) and the substituent (R) on the compound. When the substituent is Ph or Cy, the optimum alkyl chain length leans to the shorter decyl and undecyl chains (*n* = 10 and 11), while for Et and Me substitutions the dodecyl chains (*n* = 12) are more potent.

The similarities in performance between ^Ph^P2P-10,10 and ^Cy^P2P-11,11 suggest that any electronic differences that may be introduced in the change from the aromatic and electron withdrawing nature of phenyl rings to the most electron donating of the alkyl substituents, cyclohexyl, do not lead to a marked change in bioactivity. While potential explanations such as improved directionality of the alkyl chain due to the steric constraint of the larger groups, changes in lipophilicity and micellular formation of compounds containing larger substituent groups on the phosphonium atoms,^27^ and/or the ability of larger substituents to evade efflux pump resistance mechanisms^10,15^ could be proposed as influencing factors toward this empirical observation, further studies would be needed to confirm such. In consideration of the overall subtle differences in bioactivity between ^Ph^P2P-10,10, ^Cy^P2P-11,11, ^Et^P2P-12,12, and ^Me^P2P-12,12, it can be concluded that any of these phosphorus substituents can lead to promising disinfectants. In situations when atom economy is paramount, methyl-substituted phosphonium cations can serve well while enjoying a decrease in molecular weight of ∼200 g/mol compared to the phenyl- or ∼260 g/mol compared to the cyclohexyl-substituted counterparts.

Within this series, we are also able to make a direct comparison between a structurally analogous bisQAC and bisQPC. 12(2)12 and ^Me^P2P-12,12 have the same architecture, only differing in the ammonium or phosphonium cationic center. Interestingly, despite this difference in how the phosphonium and ammonium cations distribute charges, these two compounds have almost identical bioactivity across the bacterial panel, which parallels the similarity observed in the dynamic surface tension measurements. This comparable bioactivity would imply the structural feature of alkyl chain length has a much greater influence on the MIC value than the nature of the polar headgroup in comparing these two compounds. Work is ongoing within our groups to identify and evaluate these similarities and differences in electronic and structural features of QPCs and QACs and the influence on their performance as disinfectants and propensity toward resistance mechanisms.

Pleased by the activity of our phosphonium analogs, we sought to further investigate the antibacterial properties while comparing the effects of the heteroatom on activity. To do so, we determined the minimum biofilm eradication concentration (MBEC) of bisQPC ^Me^P2P-12,12 and analogous bisQAC 12(2)12 using a pegged-lid microtiter plate assay. Both quaternary amphiphiles demonstrated an ability to eradicate established biofilm against three of the strains tested (Table 2). MBEC values for bisQAC 12(2)12 differed from a previous report, which highlights the effect that an experimental method and growth conditions may have in antibacterial evaluations.^35^ While only minimal difference in potency were observed between the ammonium and phosphonium analogs, these results demonstrated that our novel cationic biocides have superior biofilm eradication potency over commercially available QACs like didecyldimethylammonium chloride (DDAC).

**Table 2:** Minimum Biofilm Eradication Concentrations of bisQPC ^Me^P2P-12,12 and bisQAC 12(2)12, as well as QAC standard DDAC

	MBEC (μM)			
compounds	HA-MRSA	*E. faecalis*	*A. baumannii*	*P. aeruginosa*
^Me^P2P-12,12	8	8	8	>250
12(2)12	4	8	4	>250
DDAC	16	16	8	>250

Finally, the hemolysis (lysis_20_) assays for ^R^P2P-*n*,*n* compounds correlated well with antimicrobial activity and alkyl chain length (*n*); no observed impact corresponded to the substituting group (R). The best balance between lysis_20_ values and antibacterial activity was observed in the ^Cy^P2P-9,9, which displayed MIC values of 16 μM for most of the resistant bacterial strains and a modest lysis_20_ value of 125 μM. This represents an improvement in antimicrobial performance with diminished hemolysis over the commercial BAC control.

## Conclusion

A novel series of alkyl-based bisQPC compounds has been synthesized and evaluated for the ability to serve as disinfectants against a panel of bacteria, including those with previously reported AMR. The resulting compounds continue to demonstrate the effectiveness of bisQPCs as viable antibacterials, with improved bioactivity against multiple antibiotic-resistant bacterial strains over a commercial benchmark of BAC. The substituting groups of methyl, ethyl, cyclohexyl, and phenyl appended to the phosphonium cations in the bisQPCs imparted only subtle differences in bioactivity across this ^R^P2P-*n*,*n* structural motif. The largest determinant leading to low MIC values was the length of the amphiphilic tail, with 10–12 carbons continuing to serve as the optimum alkyl chain length. Comparison of the structural analogs of ^Me^P2P-12,12 and 12(2)12 showed highly analogous bioactivity and dynamic surface tension despite the change from a bisQPC to bisQAC in the respective compounds.

## Methods

### General Information

Reagents and solvents were used from Sigma-Aldrich, ThermoFisher Scientific, Strem Chemicals, and TCI America without further purification. Reaction vials with pressure relieving septum caps were purchased from Chemglass Life Sciences. All reactions were carried out under an argon atmosphere. All yields refer to spectroscopically pure compounds. NMR spectra were measured with a 400 or 500 MHz JEOL spectrophotometer as noted for each compound. Chemical shifts for the ^1^H and ^13^C NMR spectra were reported on a δ scale (ppm) downfield from TMS and internally referenced to the residual chloroform-*d* (CDCl_3_) signals of 7.26 and 77.16 ppm, respectively. ^31^P NMR spectra are reported against an external reference of 85% H_3_PO_4_. Coupling constants in ^1^H NMR spectra were calculated in Hertz. These phosphorus compounds exhibit coupling between ^31^P and ^13^C in the ^13^C NMR spectra consistent with a previously reported AA′X virtual coupling pattern which convolutes the experimentally observed coupling values for *J*_P,C_ with *J*_P,P_. Therefore, coupling values for these compounds have not been unambiguously determined.^36^ For all compounds, this diagnostic ^31^P–^13^C AA′X pattern indicates the expected number of P–C bonds consistent with the indicated compound structure. Accurate mass spectrometry data were acquired on an AB Sciex 5600+ TripleTOF using electrospray ionization in positive mode.

### General Procedure for Synthesis of bisQPCs

The appropriate 1,2-bis(dialkylphosphino)ethane was loaded into a 40 mL reaction vial with a pressure relieving septum cap which contained a magnetic stir bar. Acetonitrile and the corresponding 1-bromoalkane were added to the vial by syringe through the septum cap. The reaction vial was placed in an aluminum heating block preheated to 72–75 °C. The reaction was stirred at temperature for 5–24 h. The volatiles were removed from the vial using rotary evaporation. The products were purified by either trituration or recrystallization. All reactions were conducted under an atmosphere of argon. Detailed procedures for each compound along with all characterization data can be found in the Supporting Information.

### Dynamic Surface Tension Measurements

All samples were analyzed using a Krüss BPT Mobile bubble pressure tensiometer. Solutions of 12(2)12 and ^Me^P2P-12,12 were made in concentrations of 100, 300, and 1000 ppm using deionized water. Measurements were conducted at 22 ± 1 °C. Before each sample run, a new capillary tip was installed, and the instrument was calibrated with deionized water. The dynamic surface tension (mN/m) was measured as a function of surface bubble age (30 collection points over the range of 10 to 10000 ms).

### Biological Assays

Biological testing procedures were adapted from prior work by Melander and co-workers.^37^ For all biological assays, laboratory strains of methicillin-susceptible *Staphylococcus aureus* MSSA (SH1000), *Staphylococcus aureus* CA-MRSA (USA300–0114), *Staphylococcus aureus* HA-MRSA (ATCC 33591), *Escherichia coli* (MC4100), *Enterococcus faecalis* (OG1RF), and *Acinetobacter baumannii* (ATCC 17978) were grown with shaking at 37 °C overnight from freezer stocks in 5 mL of the indicated media: SH1000, OG1RF, MC4100, USA300-0114, and ATCC 17978 were grown in BD Mueller-Hinton broth (MHB), whereas ATCC 33591 was grown in BD tryptic soy broth (TSB). The inoculum was then streaked onto Mueller-Hinton agar (MHA) plates and grown overnight at 37 °C. From the plates, single colonies were selected, diluted into 50 mL of fresh media, and grown overnight at 37 °C with shaking for use in the MIC assays. Optical density (OD) measurements were obtained using a SpectraMax iD3 plate reader (Molecular Devices, United States).

### Minimum Inhibitory Concentration (MIC)

Compounds were serially diluted 2-fold from stock solutions (1.0 mM) to yield 12 100-μL test concentrations, wherein the starting concentration of dimethyl sulfoxide (DMSO) was 2.5%. Overnight *S. aureus*, *E. faecalis*, *E. coli*, *A. baumannii*, USA300-0114 (CA-MRSA), and ATCC 33591 (HA-MRSA) cultures were diluted to ca. 10^6^ CFU/mL in MHB or TSB and regrown to midexponential phase, as determined by optical density recorded at 600 nm (OD_600_). All cultures were then diluted again to ca. 10^6^ CFU/mL, and 100 μL was inoculated into each well of a U-bottom 96-well plate (Corning, 351177) containing 100 μL of compound solution. Plates were incubated statically at 37 °C for 72 h, upon which wells were evaluated visually for bacterial growth. The MIC was determined as the lowest concentration of compound resulting in no bacterial growth visible to the naked eye, based on the highest value in three independent experiments. Aqueous DMSO controls were conducted as appropriate for each compound.

### Red Blood Cell (RBC) Lysis Assay (lysis_20_)

RBC lysis assays were performed on mechanically defibrinated sheep blood (Hemostat Laboratories: DSB030). An aliquot of 1.5 mL blood was placed into a microcentrifuge tube and centrifuged at 3800 rpm for 10 min. The supernatant was removed, and the cells were resuspended with 1 mL of phosphate-buffered saline (PBS). The suspension was centrifuged as described above, the supernatant was removed, and cells were resuspended four additional times in 1 mL of PBS. The final cell suspension was diluted 20-fold with PBS. Compounds were serially diluted with PBS 2-fold from stock solutions (1.0 mM) to yield 100 μL of 12 test concentrations on a flat-bottom 96-well plate (Corning, 351172), wherein the starting concentration of DMSO was 2.5%. To each of the wells, 100 μL of the 20-fold suspension dilution was then inoculated. TritonX (1% by volume) served as a positive control (100% lysis marker), and sterile PBS served as a negative control (0% lysis marker). Samples were then placed in an incubator at 37 °C and shaken at 200 rpm. After 1 h, the samples were centrifuged at 3800 rpm for 10 min. The absorbance of the supernatant was measured with a UV spectrometer at a 540 nm wavelength. The concentration inducing 20% RBC lysis was then calculated for each compound based upon the absorbances of the TritonX and PBS controls. Aqueous DMSO controls were conducted as appropriate for each compound.

## Supporting Information Available

Detailed synthetic procedures; characterization data; NMR spectra (PDF)

## Abbreviations

QAC: quaternary ammonium compound
QPC: quaternary phosphonium compound
AMR: antimicrobial resistance
BAC: benzalkonium chloride
TSC: trivalent sulfonium compound
NMR: nuclear magnetic resonance
HSQC: heteronuclear single quantum coherence spectroscopy
MSSA: methicillin-susceptible *Staphylococcus aureus*
CA-MRSA: community-acquired methicillin-resistant *Staphylococcus aureus*
HA-MRSA: hospital-acquired methicillin-resistant *Staphylococcus aureus*
MIC: minimum inhibitory concentration
DMSO: dimethyl sulfoxide
PBS: phosphate-buffered saline
