# Supplementary material for: Atom Economical QPCs: Phenyl-Free Biscationic Quaternary Phosphonium Compounds as Potent Disinfectants
Source: ACS Infect Dis. 2023 Feb 9;9(3):609–16. doi: 10.1021/acsinfecdis.2c00575 (PMC10032568; doi:10.1021/acsinfecdis.2c00575)
Supplement: Supplementary file 1 — id2c00575_si_001.pdf [file id2c00575_si_001.pdf]

Supporting Information

Atom Economical QPCs: Phenyl-free Biscationic Quaternary Phosphonium Compounds as Potent Disinfectants

Laura M. Thierer,[a] Ashley A. Petersen,[a] Marina E. Michaud,[b] Christian A. Sanchez,[b] Samantha R. Brayton,[a] Prof. William M. Wuest,*[b] and Prof. Kevin P.C. Minbiole*[a]

[a] *Department of Chemistry, Villanova University, Villanova, PA 19085, USA*
[b] *Department of Chemistry, Emory University, Atlanta, GA 30322, USA*
**Corresponding authors*
*Email: kevin.minbiole@villanova.edu

Table of Contents

I. Synthetic Procedures……………………………………………………………………..S2

II. NMR Spectroscopic Data……………………………………………………………….S20

III. Biofilm Eradication Procedure…………………………………………………………S54

I. Synthetic Procedures

Preparation of ^{Me}P2P-8,8-Br

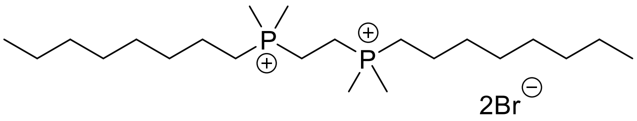

1,2-Bis(dimethylphosphino)ethane (0.35 mL, 0.32 g, 2.1 mmol) was loaded via syringe to a 40 mL reaction vial with pressure relieving septum cap which contained a magnetic stir bar and was flushed with argon for 15 minutes prior to the addition. Acetonitrile (0.7 mL) and 1-bromooctane (1.00 mL, 1.12 g, 5.8 mmol) were added to the vial also by syringe. The reaction vial was placed in an aluminum heating block preheated to 75 °C, venting with a needle twice during the first 5 minutes to avoid over pressurization of the vial. The reaction was stirred for 5 hours at 75 °C, and then allowed to further stir at room temperature for 18 h. The volatiles were removed from the vial using rotary evaporation. The resulting colorless, viscous oil was triturated with diethyl ether to form a white solid. The product was isolated via vacuum filtration as a fine white powder (0.759 g, 67.5 %);^{1}H NMR ($CDCl_3$, 500 MHz): δ 3.38 (br, 4H), 2.48 (br, 4H), 2.18 (t, 12H, $J_{P,H}$ = 5.0 Hz,), 1.53 (br, 4H), 1.42 (br quint, 4H), 1.28-1.18 (br m, 16H), 0.82 (t, 6H, $J_{H,H}$ = 5 Hz); ^{13}C{^{1}H} NMR ($CDCl_3$, 125.8 MHz): δ 31.74, 30.72 (t, J = 7.5 Hz), 29.09, 22.61, 22.06 (t), 21.70 (t, J = 2.5 Hz), 15.67 (quint), 14.12, 6.87 (quint); ^{31}P{^{1}H} NMR ($CDCl_3$, 162.0 MHz): δ 33.55; HRMS (ESI+): Found 188.1677, expected $C_{22}H_{50}P_2$ [M-2Br$^-$]$^{2+}$ 188.1700.

Preparation of ^{Me}P2P-9,9-Br

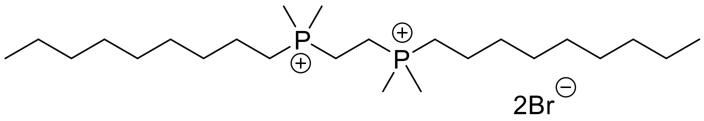

1,2-Bis(dimethylphosphino)ethane (0.40 mL, 0.36 g, 2.4 mmol) was loaded via syringe to a 40 mL reaction vial with pressure relieving septum cap which contained a magnetic stir bar and was flushed with argon for 15 minutes prior to the addition. Acetonitrile (0.8 mL) and 1-bromononane (1.30 mL, 1.41 g, 6.8 mmol) were added to the vial also by syringe. The reaction vial was placed in an aluminum heating block preheated

to 72 °C, venting with a needle twice during the first 5 minutes to avoid over pressurization of the vial. The reaction was stirred for 7 hours at 72 °C, and then cooled to room temperature. The volatiles were removed from the vial using rotary evaporation. The resulting waxy, white solid was triturated with 8 mL of hexanes and the slurry was placed into a -20 °C freezer overnight. The product was isolated via vacuum filtration, then triturated again with 12 mL of diethyl ether and vacuum filtered to afford a white waxy solid. (1.26 g, 93.0 %); ^{1}H NMR ($CDCl_3$, 500 MHz): δ 3.24 (s, 4H), 2.48 (br, 4H), 2.17 (t, 12H, $J_{P,H}$ = 5.0 Hz,), 1.52 (br, 4H), 1.40 (br quint, 4H), 1.27-1.18 (br m, 20H), 0.80 (t, 6H, $J_{H,H}$ = 7.5 Hz); ^{13}C{^{1}H} NMR ($CDCl_3$, 125.8 MHz): δ 31.76, 30.68 (t, J = 8.2 Hz), 29.34, 29.18, 29.09, 22.60, 22.12 (t), 21.68 (t, J = 1.9 Hz), 15.65 (quint), 14.09, 6.92 (quint); ^{31}P{^{1}H} NMR ($CDCl_3$, 202.4 MHz): δ 33.31; HRMS (ESI+): Found 202.1834, expected $C_{24}H_{54}P_2$ [M-2Br$^-$]$^{2+}$ 202.1850.

Preparation of ^{Me}P2P-10,10-Br

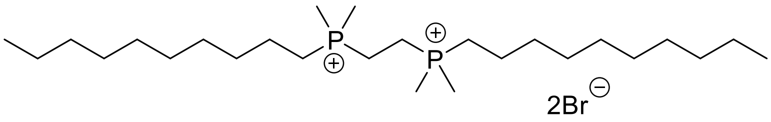

1,2-Bis(dimethylphosphino)ethane (0.40 mL, 0.36 g, 2.4 mmol) was loaded via syringe to a 40 mL reaction vial with pressure relieving septum cap which contained a magnetic stir bar and was flushed with argon for 15 minutes prior to the addition. Acetonitrile (0.7 mL) and 1-bromodecane (1.30 mL, 1.39 g, 6.3 mmol) were added to the vial also by syringe. The reaction vial was placed in an aluminum heating block preheated to 75 °C, venting with a needle twice during the first 5 minutes to avoid over pressurization of the vial. The reaction was stirred for 5 hours at 75 °C, and then allowed to further stir at room temperature for 18 h. The volatiles were removed from the vial using rotary evaporation. The resulting colorless, viscous oil was triturated with diethyl ether to form a white solid. The product was isolated via vacuum filtration as a fine white powder (1.41 g, 99.0%); ^{1}H NMR ($CDCl_3$, 500 MHz): δ 3.32 (d, 4H, $J_{P,H}$ = 5.0 Hz), 2.49 (br, 4H), 2.18 (t, 12H, $J_{P,H}$ = 5.0 Hz,), 1.52 (br, 4H), 1.40 (br quint, 4H), 1.27-1.18 (br m, 24H), 0.80 (t, 6H, $J_{H,H}$ = 7.5 Hz); ^{13}C{^{1}H} NMR ($CDCl_3$, 125.8 MHz): δ 31.81, 30.69 (br t), 29.46, 29.37, 29.23, 29.08, 22.61, 22.19 (t),

21.68, 15.65 (t), 14.07, 6.95 (t); $^{31}P\{^1H\}$ NMR ($CDCl_3$, 202.4 MHz): δ 33.67; HRMS (ESI+): Found 216.1993, expected $C_{26}H_{58}P_2$ $[M]^{2+}$ 216.2000.

Preparation of ^{Me}P2P-11,11-Br

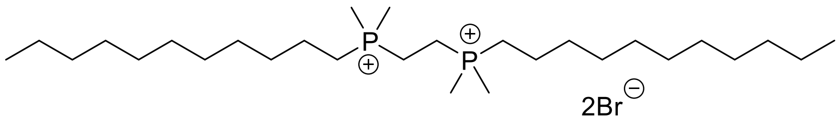

1,2-Bis(dimethylphosphino)ethane (0.40 mL, 0.36 g, 2.4 mmol) was loaded via syringe to a 40 mL reaction vial with pressure relieving septum cap which contained a magnetic stir bar and was flushed with argon for 15 minutes prior to the addition. Acetonitrile (0.6 mL) and 1-bromoundecane (1.60 mL, 1.69 g, 7.2 mmol) were added to the vial also by syringe. The reaction vial was placed in an aluminum heating block preheated to 72 °C, venting with a needle twice during the first 5 minutes to avoid over pressurization of the vial. The reaction was stirred for 5 hours at 72 °C, and then allowed to further stir at room temperature overnight. The volatiles were removed from the vial using rotary evaporation. The resulting colorless, viscous oil was dissolved into ~2 mL of dichloromethane, layered with ~30 mL hexanes and placed into a -20 °C freezer. After 3 h in the freezer, the vial was swirled to help initiate the formation of a white solid, then was allowed to sit in the freezer for 2 days. The product was isolated via vacuum filtration as a fine white powder (1.335 g, 89.7%); ^1H NMR ($CDCl_3$, 500 MHz): δ 3.33 (d, 4H, $J_{P,H}$ = 5.0 Hz), 2.49 (br, 4H), 2.19 (t, 12H, $J_{P,H}$ = 7.5 Hz,), 1.53 (br, 4H), 1.41 (br, 4H), 1.26-1.19 (br m, 28H), 0.81 (t, 6H, $J_{H,H}$ = 7.5 Hz);); $^{13}C\{^1H\}$ NMR ($CDCl_3$, 125.8 MHz): δ 31.86, 30.77 (br t), 29.56, 29.54, 29.40, 29.30, 29.11, 22.65, 22.23 (t), 21.71, 15.70 (t), 14.10, 6.98 (t); $^{31}P\{^1H\}$ NMR ($CDCl_3$, 202.4 MHz): δ 33.65; HRMS (ESI+): Found 230.2151, expected $C_{28}H_{62}P_2$ $[M-2Br^-]^{2+}$ 230.2150.

Preparation of ^{Me}P2P-12,12-Br

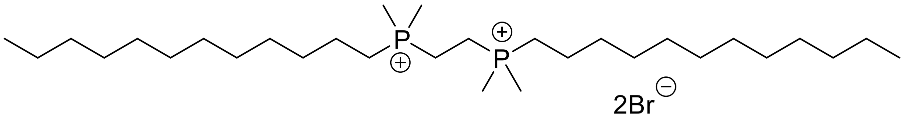

1,2-Bis(dimethylphosphino)ethane (0.30 mL, 0.27 g, 1.8 mmol) was loaded via syringe to a 40 mL reaction vial with pressure relieving septum cap which contained a magnetic stir bar and was flushed with argon for 15 minutes prior to the addition. Acetonitrile (0.6 mL) and 1-bromododecane (1.30 mL, 1.35 g, 5.4 mmol) were added to the vial also by syringe. The reaction vial was placed in an aluminum heating block preheated to 72 °C, venting with a needle twice during the first 5 minutes to avoid over pressurization of the vial. The reaction was stirred for 5 hours at 72 °C, and then allowed to further stir at room temperature overnight. The volatiles were removed from the vial using rotary evaporation. The resulting colorless, viscous oil was dissolved into ~2 mL of dichloromethane, layered with ~30 mL hexanes and placed into a -20 °C freezer. After 3 h in the freezer, the vial was swirled to help initiate the formation of a white solid, then was allowed to sit in the freezer for 2 days. The product was isolated via vacuum filtration as a fine white powder (0.986 g, 84.6%); ^{1}H NMR ($CDCl_3$, 500 MHz): δ 3.24 (s, 4H), 2.47 (br, 4H), 2.16 (t, 12H, $J_{P,H}$ = 7.5 Hz,), 1.52 (br, 4H), 1.39 (br quint, 4H), 1.26-1.17 (br m, 32H), 0.80 (t, 6H, $J_{H,H}$ = 7.5 Hz); ^{13}C{^{1}H} NMR ($CDCl_3$, 125.8 MHz): δ 31.82, 30.68 (t, J = 8.2 Hz), 29.60, 29.58, 29.54, 29.40, 29.28, 29.10, 22.60, 22.11 (t), 21.65, 15.64 (quint), 14.05, 6.89 (quint); ^{31}P{^{1}H} NMR ($CDCl_3$, 202.4 MHz): δ 33.73; HRMS (ESI+): Found 244.2317, expected $C_{30}H_{66}P_2$ [M-2Br$^-$]$^{2+}$ 244.2300.

Preparation of ^{Me}P2P-14,14-Br

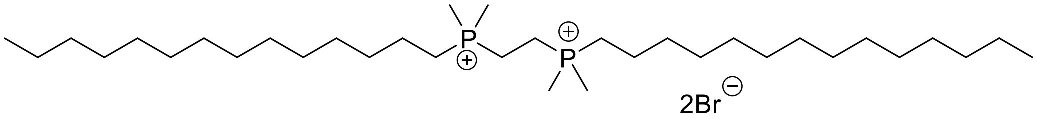

1,2-Bis(dimethylphosphino)ethane (0.30 mL, 0.27 g, 1.8 mmol) was loaded via syringe to a 40 mL reaction vial with pressure relieving septum cap which contained a magnetic stir bar and was flushed with argon for

15 minutes prior to the addition. Acetonitrile (0.7 mL) and 1-bromotetradecane (1.60 mL, 1.49 g, 5.4 mmol) were added to the vial also by syringe. The reaction vial was placed in an aluminum heating block preheated to 72 °C, venting with a needle twice during the first 5 minutes to avoid over pressurization of the vial. The reaction was stirred for 7 hours at 72 °C, and then cooled to room temperature. The volatiles were removed from the vial using rotary evaporation. The resulting waxy, white solid was triturated with 8 mL of hexanes and the slurry was placed into a -20 °C freezer overnight. The product was isolated via vacuum filtration, then triturated again with 12 mL of diethyl ether and vacuum filtered to afford a fine white powder. (1.02 g, 80.3 %); ^{1}H NMR ($CDCl_3$, 500 MHz): δ 3.36 (d, 4H, $J_{P,H}$ = 5.0 Hz), 2.53 (br, 4H), 2.21 (t, 12H, $J_{P,H}$ = 5.0 Hz,), 1.57 (br, 4H), 1.47 (br quint, 4H), 1.33-1.24 (br m, 40H), 0.87 (t, 6H, $J_{H,H}$ = 7.5 Hz); ^{13}C{^{1}H} NMR ($CDCl_3$, 125.8 MHz): δ 32.03, 30.77 (t, J = 7.5 Hz), 29.81, 29.79, 29.77, 29.75, 29.67, 29.50, 29.48, 29.22, 22.80, 22.49 (t), 21.82, 16.03 (t), 14.24, 7.21 (quint); ^{31}P{^{1}H} NMR ($CDCl_3$, 162.0 MHz): δ 33.74; HRMS (ESI+): Found 272.2633, expected $C_{34}H_{74}P_2$ [M-2Br$^-$]$^{2+}$ 272.2650.

Preparation of ^{Me}P2P-16,16-Br

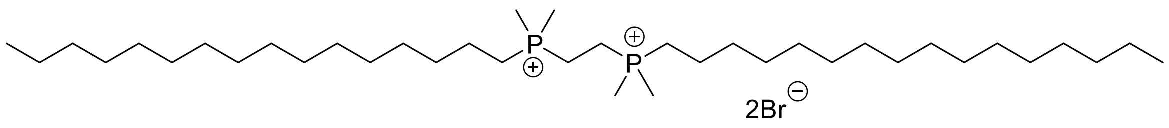

1,2-Bis(dimethylphosphino)ethane (0.30 mL, 0.27 g, 1.8 mmol) was loaded via syringe to a 40 mL reaction vial with pressure relieving septum cap which contained a magnetic stir bar and was flushed with argon for 15 minutes prior to the addition. Acetonitrile (0.7 mL) and 1-bromohexadecane (1.65 mL, 1.65 g, 5.4 mmol) were added to the vial also by syringe. The reaction vial was placed in an aluminum heating block preheated to 72 °C, venting with a needle twice during the first 5 minutes to avoid over pressurization of the vial. The reaction was stirred for 7 hours at 72 °C, and then cooled to room temperature. The volatiles were removed from the vial using rotary evaporation. The resulting waxy, white solid was triturated with 8 mL of hexanes and the slurry was placed into a -20 °C freezer overnight. The product was isolated via vacuum filtration, then triturated again with 12 mL of diethyl ether and vacuum filtered to afford a fine white powder. (0.909

g, 66.4 %); ^{1}H NMR ($CDCl_3$, 500 MHz): δ 3.36 (d, 4H, $J_{P,H}$ = 5.0 Hz), 2.53 (br, 4H), 2.21 (t, 12H, $J_{P,H}$ = 5.0 Hz,), 1.57 (br, 4H), 1.47 (br quint, 4H), 1.33-1.24 (br m, 40H), 0.87 (t, 6H, $J_{H,H}$ = 7.5 Hz); $^{13}C\{^{1}H\}$ NMR ($CDCl_3$, 125.8 MHz): δ 31.97, 30.78 (t, J = 8.2 Hz), 29.78, 29.75, 29.72, 29.68, 29.52, 29.41, 29.22, 22.73, 22.23 (t), 21.78, 15.78 (quint), 14.18, 7.01 (quint); $^{31}P\{^{1}H\}$ NMR ($CDCl_3$, 202.4 MHz): δ 33.70; HRMS (ESI+): Found 300.2946, expected $C_{38}H_{82}P_2$ [M-2Br$^-$]$^{2+}$ 300.2950.

Preparation of ^{Et}P2P-8,8-Br

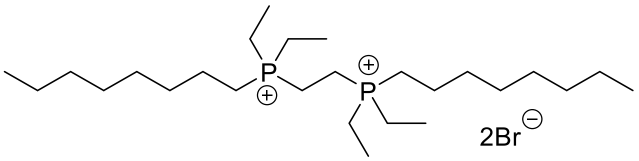

1,2-Bis(diethylphosphino)ethane (0.25 mL, 0.22 g, 1.1 mmol) was loaded via syringe to a 40 mL reaction vial with pressure relieving septum cap which contained a magnetic stir bar and was flushed with argon for 15 minutes prior to the addition. Acetonitrile (1 mL) and 1-bromooctane (0.55 mL, 0.61 g, 3.2 mmol) were added to the vial also by syringe. The reaction vial was placed in an aluminum heating block preheated to 70 °C, venting with a needle twice during the first 5 minutes to avoid over pressurization of the vial. The reaction was stirred for 5 h at 70 °C, then cooled to room temperature and stirred for 15 h. At the end of the stir time, the volatiles were removed from the vial using rotary evaporation. The resulting colorless, viscous oil was dissolved into ~2 mL of dichloromethane, layered with ~30 mL hexanes and placed into a -20 °C freezer. After 3 h in the freezer, the vial was swirled to help initiate the formation of a white solid, then was allowed to sit in the freezer for 1 day. The product was isolated via vacuum filtration as a fine white powder (0.404 g, 63.8%); ^{1}H NMR ($CDCl_3$, 500 MHz): δ 3.29 (d, 4H, $J_{P,H}$ = 5.0 Hz), 2.65 (br sextet, 8H), 2.62 (br q, 4H), 1.55 (br, 4H), 1.45 (br quint, 4H), 1.33-1.20 (br m, 28H), 0.81 (t, 6H, $J_{H,H}$ = 7.5 Hz); $^{13}C\{^{1}H\}$ NMR ($CDCl_3$, 125.8 MHz): δ 31.66, 30.85, 28.99, 22.57, 21.88 (br t), 18.53 (quint), 14.06, 12.69 (t), 6.37-6.25 (m, two signals); $^{31}P\{^{1}H\}$ NMR ($CDCl_3$, 202.4 MHz): δ 40.14; HRMS (ESI+): Found 216.1996, expected $C_{26}H_{58}P_2$ [M-2Br$^-$]$^{2+}$ 216.2000.

Preparation of ^{Et}P2P-10,10-Br

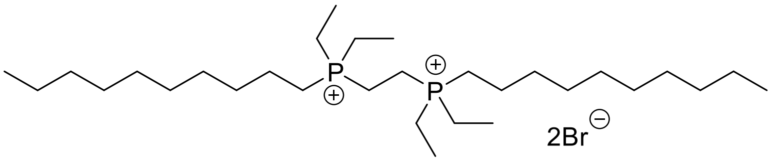

1,2-Bis(diethylphosphino)ethane (0.28 mL, 0.25 g, 1.2 mmol) was loaded via syringe to a 40 mL reaction vial with pressure relieving septum cap which contained a magnetic stir bar and was flushed with argon for 15 minutes prior to the addition. Acetonitrile (1 mL) and 1-bromodecane (0.75 mL, 0.80 g, 3.6 mmol) were added to the vial also by syringe. The reaction vial was placed in an aluminum heating block preheated to 70 °C, venting with a needle twice during the first 5 minutes to avoid over pressurization of the vial. The reaction was stirred for 6 h at 75 °C, then cooled to room temperature. The volatiles were removed from the vial using rotary evaporation. The resulting colorless, viscous oil was dissolved into ~2 mL of dichloromethane, layered with ~30 mL hexanes and placed into a -20 °C freezer. After 3 h in the freezer, the vial was swirled to help initiate the formation of a white solid, then was allowed to sit in the freezer for 2 days. The product was isolated via vacuum filtration as a fine white powder (0.689 g, 87.6%); ^{1}H NMR ($CDCl_3$, 500 MHz): δ 3.15 (d, 4H, $J_{P,H}$ = 10.0 Hz), 2.56 (br sextet, 8H), 2.48 (br, 4H), 1.51 (br, 4H), 1.41 (br quint, 4H), 1.28-1.16 (br m, 36H), 0.78 (t, 6H, $J_{H,H}$ = 7.5 Hz); ^{13}C{^{1}H} NMR ($CDCl_3$, 125.8 MHz): δ 31.75, 30.77, 29.39, 29.30, 29.17, 28.97, 22.55, 21.76. 18.24 (t), 14.01, 12.40 (t), 6.29-6.14 (m); ^{31}P{^{1}H} NMR ($CDCl_3$, 202.4 MHz): δ 40.40; HRMS (ESI+): Found 244.2311, expected $C_{30}H_{66}P_2$ [M-2Br$^-$]$^{2+}$ 244.2300.

Preparation of ^{Et}P2P-11,11-Br

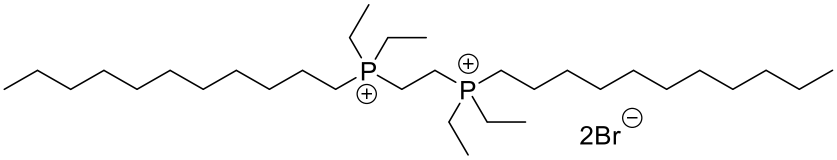

1,2-Bis(diethylphosphino)ethane (0.28 mL, 0.25 g, 1.2 mmol) was loaded via syringe to a 40 mL reaction vial with pressure relieving septum cap which contained a magnetic stir bar and was flushed with argon for

15 minutes prior to the addition. Acetonitrile (1 mL) and 1-bromodecane (0.75 mL, 0.80 g, 3.6 mmol) were added to the vial also by syringe. The reaction vial was placed in an aluminum heating block preheated to 70 °C, venting with a needle twice during the first 5 minutes to avoid over pressurization of the vial. The reaction was stirred for 6 h at 75 °C, then cooled to room temperature. The volatiles were removed from the vial using rotary evaporation. The resulting colorless, viscous oil was dissolved into ~2 mL of dichloromethane, layered with ~30 mL hexanes and placed into a -20 °C freezer. After 3 h in the freezer, the vial was swirled to help initiate the formation of a white solid, then was allowed to sit in the freezer for 2 days. The product was isolated via vacuum filtration as a fine white powder (0.488 g, 59.4%); ^{1}H NMR ($CDCl_3$, 500 MHz): δ 3.26 (d, 4H, $J_{P,H}$ = 10.0 Hz), 2.61 (br sextet, 8H), 2.51 (br q, 4H), 1.54 (br, 4H), 1.44 (br quint, 4H), 1.32-1.18 (br m, 40H), 0.81 (t, 6H, $J_{H,H}$ = 7.5 Hz); ^{13}C{^{1}H} NMR ($CDCl_3$, 125.8 MHz): δ 31.83, 30.84, 29.53, 29.48, 29.34, 29.27, 29.03, 22.64, 21.87 (t), 18.48 (t), 14.09, 12.63 (t), 6.34-6.26 (m, two signals); ^{31}P{^{1}H} NMR ($CDCl_3$, 202.4 MHz): δ 40.20; HRMS (ESI+): Found 258.2476, expected $C_{32}H_{70}P_2$ $[M-2Br^-]^{2+}$ 258.2500.

Preparation of ^{Et}P2P-12,12-Br

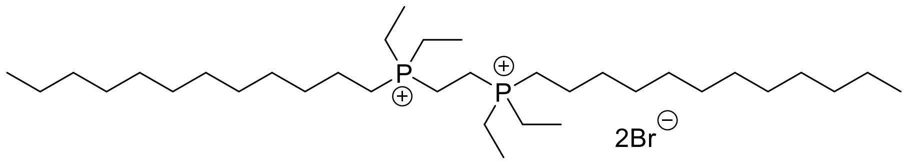

1,2-Bis(diethylphosphino)ethane (0.28 mL, 0.25 g, 1.2 mmol) was loaded via syringe to a 40 mL reaction vial with pressure relieving septum cap which contained a magnetic stir bar and was flushed with argon for 15 minutes prior to the addition. Acetonitrile (1 mL) and 1-bromododecane (0.87 mL, 0.90 g, 3.6 mmol) were added to the vial, also by syringe. The reaction vial was placed in an aluminum heating block preheated to 75 °C, venting with a needle twice during the first 5 minutes to avoid over pressurization of the vial. The reaction was stirred for 6 h at 75 °C, then cooled to room temperature. The volatiles were removed from the vial using rotary evaporation. The resulting colorless, viscous oil was dissolved into ~2 mL of dichloromethane, layered with ~30 mL hexanes and placed into a -20 °C freezer. After 3 h in the freezer, the

vial was swirled to help initiate the formation of a white solid, then was allowed to sit in the freezer for 2 days. The product was isolated via vacuum filtration as a fine white powder (0.539 g, 63.7%); ^{1}H NMR ($CDCl_3$, 500 MHz): δ 3.17 (br, 4H), 2.60 (br sextet, 8H), 2.49 (br, 4H), 1.54 (br, 4H), 1.44 (br, 4H), 1.31-1.18 (br m, 44H), 0.81 (t, 6H, $J_{H,H}$ = 7.5 Hz); ^{13}C{^{1}H} NMR ($CDCl_3$, 125.8 MHz): δ 31.87, 30.83, 29.61, 29.59, 29.52, 29.38, 29.31, 29.05, 22.65, 21.85, 18.30 (t), 14.10, 12.50 (t), 6.31-6.21 (m, two signals); ^{31}P{^{1}H} NMR ($CDCl_3$, 202.4 MHz): δ 40.37; HRMS (ESI+): Found 272.2624, expected $C_{34}H_{74}P_2$ $[M-2Br^{-}]^{2+}$ 272.2650.

Preparation of ^{Et}P2P-14,14-Br

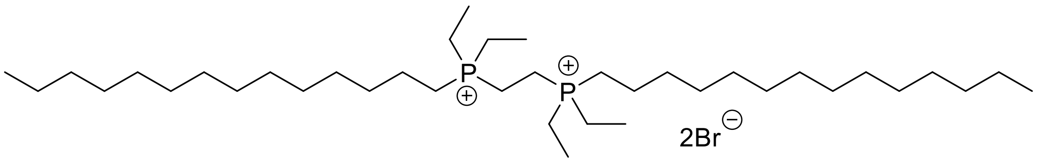

1,2-Bis(diethylphosphino)ethane (0.26 mL, 0.23 g, 1.1 mmol) was loaded via syringe to a 40 mL reaction vial with pressure relieving septum cap which contained a magnetic stir bar and was flushed with argon for 15 minutes prior to the addition. Acetonitrile (1 mL) and 1-bromotetradecane (1.0 mL, 0.93 g, 3.4 mmol) were added to the vial also by syringe. The reaction vial was placed in an aluminum heating block preheated to 70 °C, venting with a needle twice during the first 5 minutes to avoid over pressurization of the vial. The reaction was stirred for 5 h at 70 °C, then cooled to room temperature and stirred for 15 h. At the end of the stir time, the volatiles were removed from the vial using rotary evaporation. The resulting colorless, viscous oil was dissolved into ~2 mL of dichloromethane, layered with ~30 mL hexanes and placed into a -20 °C freezer. After 3 h in the freezer, the vial was swirled to help initiate the formation of a white solid, then was allowed to sit in the freezer for 1 day. The product was isolated via vacuum filtration as a fine white powder (0.744 g, 87.7 %); ^{1}H NMR ($CDCl_3$, 500 MHz): δ 3.22 (br, 4H), 2.60 (br, 8H), 2.50 (br, 4H), 1.54 (br, 4H), 1.43 (br, 4H), 1.32-1.19 (br m, 52H), 0.82 (t, 6H, $J_{H,H}$ = 7.5 Hz); ^{13}C{^{1}H} NMR ($CDCl_3$, 125.8 MHz): δ 31.85, 30.82, 29.61, 29.58, 29.50, 29.35, 29.29, 29.03, 22.62, 21.82, 18.32 (t), 14.07, 12.49 (t), 6.27-6.19 (m,

two signals); $^{31}P\{^1H\}$ NMR ($CDCl_3$, 202.4 MHz): δ 40.29; HRMS (ESI+): Found 300.2931, expected $C_{38}H_{74}P_2$ $[M-2Br^-]^{2+}$ 300.2950.

Preparation of ^{Et}P2P-16,16-Br

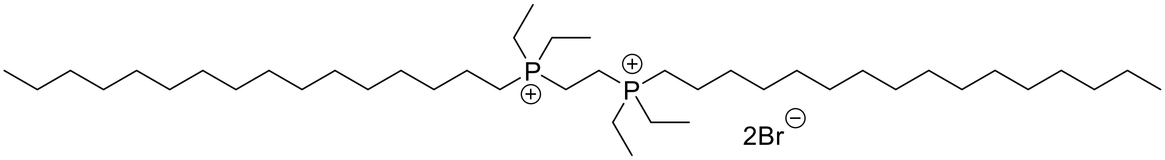

1,2-Bis(diethylphosphino)ethane (0.26 mL, 0.23 g, 1.1 mmol) was loaded via syringe to a 40 mL reaction vial with pressure relieving septum cap which contained a magnetic stir bar and was flushed with argon for 15 minutes prior to the addition. Acetonitrile (1 mL) and 1-bromohexadecane (1.0 mL, 1.0 g, 3.3 mmol) were added to the vial also by syringe. The reaction vial was placed in an aluminum heating block preheated to 70 °C, venting with a needle twice during the first 5 minutes to avoid over pressurization of the vial. The reaction was stirred for 5 h at 70 °C, then cooled to room temperature and stirred for 15 h. At the end of the stir time, the volatiles were removed from the vial using rotary evaporation. The resulting colorless, viscous oil was dissolved into ~2 mL of dichloromethane, layered with ~30 mL hexanes and placed into a -20 °C freezer. After 3 h in the freezer, the vial was swirled to help initiate the formation of a white solid, then was allowed to sit in the freezer for 1 day. The product was isolated via vacuum filtration as a fine white powder (0.870 g, 95.5 %); ^1H NMR ($CDCl_3$, 500 MHz): δ 3.22 (br, 4H), 2.60 (br, 8H), 2.51 (br, 4H), 1.55 (br, 4H), 1.45 (br, 4H), 1.32-1.19 (br m, 60H), 0.82 (t, 6H, $J_{H,H}$ = 5.0 Hz); $^{13}C\{^1H\}$ NMR ($CDCl_3$, 125.8 MHz): δ 31.92, 30.87 (br t), 29.71, 29.68, 29.65, 29.56, 29.40, 29.36, 29.08, 22.69, 21.89, 18.43 (t), 14.13, 12.59 (t), 6.35-6.25 (m, two signals); $^{31}P\{^1H\}$ NMR ($CDCl_3$, 202.4 MHz): δ 40.30; HRMS (ESI+): Found 328.3245, expected $C_{42}H_{90}P_2$ $[M-2Br^-]^{2+}$ 328.3250.

Preparation of ^{Cy}P2P-8,8-Br

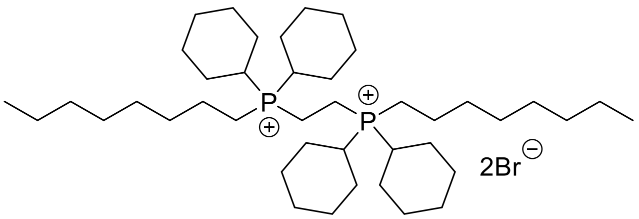

1,2-Bis(dicyclohexylphosphino)ethane (0.211 g, 0.5 mmol) was massed into a 40 mL reaction vial with pressure relieving septa cap equipped with a stir bar which was preliminarily flushed with argon for 15 minutes. After the addition of 1,2-bis(dicyclohexylphosphino)ethane, the reaction vial was flushed with argon for an additional 10 minutes. Acetonitrile (1 mL) and 1-bromooctane (0.18 mL, 0.20 g, 1.0 mmol) were added to the reaction vial via needle and syringe. The reaction vial was placed in an aluminum heating block which was preheated to 72 ^{o}C and vented by puncturing the cap with a needle twice to relieve pressure buildup. The reaction vial was stirred at 72 ^{o}C for 24 hours. Within 45 minutes, the reaction contents changed from a slurry to a clear solution and remained as a solution through the entire reaction time. At the end of 24 hours, the reaction vial was removed from heat and cooled to room temperature. All volatiles were removed by rotary evaporation. The resulting white gel was dissolved in (approx. 2 mL) dichloromethane layered with (approx. 30 mL) hexanes and place in a -20 ˚C freezer. After two days, the mother liquor was decanted from the oil that formed on the bottom of the vial. Residual solvent was again removed by rotary evaporation, resulting in the product as a white, glassy solid. (0.375 g, 94.6 %); ^{1}H NMR ($CDCl_3$, 500 MHz), δ 3.09 (d, *J* = 5.3 H, 4H) 3.06-2.93 (m, 4H) 2.58 (m, 4H) 2.07 – 1.66 (m, 20H) 1.56 – 1.38 (m, 24H) 1.30 – 1.13 (m, 20H) 0.79 (t, *J* = 6.8 Hz, 6H) ; ^{13}C{^{1}H} NMR ($CDCl_3$, 125.8 MHz) δ 31.72, 31.06 (t, *J* = 7.1 Hz) 30.68 (t, *J* = 49.06) 29.08, 29.03, 27.02, 26.01 (t, *J* = 6.2 Hz), 25.34, 22.72 (t, *J* = 2.2 Hz), 22.63, 16.51, 14.01, 11.83 ^{31}P{^{1}H} NMR ($CDCl_3$, 202.5 MHz*)*, δ 36.88. HRMS (ESI+): Found 324.2931, expected m/z $C_{42}H_{82}P_2$ [M-2Br]$^{2+}$ 324.2941.

Preparation of ^{Cy}P2P-9,9-Br

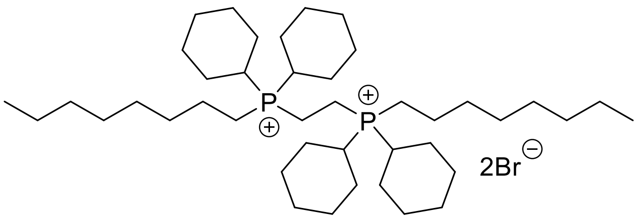

1,2-Bis(dicyclohexylphosphino)ethane (0.211 g, 0.5 mmol) was massed into a 40 mL reaction vial with pressure relieving septa cap equipped with a stir bar which was preliminarily flushed with argon for 15 minutes. After the addition of 1,2-bis(dicyclohexylphosphino)ethane, the reaction vial was flushed with argon for an additional 10 minutes. Acetonitrile (1 mL) and 1-bromononane (0.20 mL, 0.22 g, 1.0 mmol) were added to the reaction vial via needle and syringe. The reaction vial was placed in an aluminum heating block which was preheated to 72 ^{o}C and vented by loosening the cap twice to relieve pressure buildup. The reaction vial was stirred at 72 ^{o}C for 24 hours. Within 45 minutes, the reaction contents changed from a slurry to a clear solution and remained as a solution through the entire reaction time. At the end of 24 hours, the reaction vial was removed from heat and cooled to room temperature. All volatiles were removed by rotary evaporation. The resulting white powder was purified via trituration with diethyl ether (approx. 30 mL) and isolated by vacuum filtration, resulting in the product as a white solid. (0.387 g, 92.4 %); ^{1}H NMR ($CDCl_3$, 500 MHz), δ 3.19 (d, *J* = 5.5 H, 4H) 3.04 - 2.97 (m, 4H) 2.62 – 2.55 (m, 4H) 2.07 – 1.70 (m, 20H) 1.60 – 1.34 (m, 24H) 1.35 – 1.10 (m, 25H) 0.80 (t, *J* = 6.9 Hz, 6H) ; ^{13}C{^{1}H} NMR ($CDCl_3$, 125.8 MHz) δ 31.85, 31.07 (t, *J* = 6.3 Hz) 30.68 (t, *J* = 20.1) 29.34, 29.21, 29.16, 27.12, 26.10 (t, *J* = 6.3 Hz), 25.44, 22.74 (t, *J* = 2.5 Hz), 22.67, 16.53, 14.13, 11.87 ^{31}P{^{1}H} NMR ($CDCl_3$, 202.5 MHz), δ 36.87. HRMS (ESI+): Found 338.3095, expected m/z $C_{44}H_{86}P_2$ [M-2Br]$^{2+}$ 338.3097.

Preparation of ^{Cy}P2P-10,10-Br

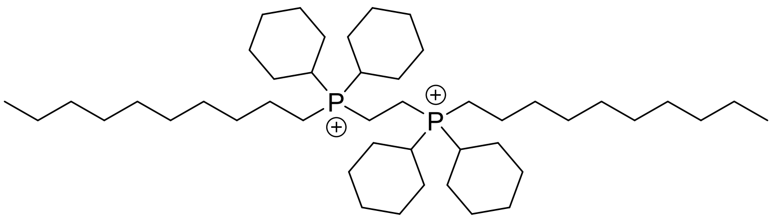

1,2-Bis(dicyclohexylphosphino)ethane (0.211 g, 0.5 mmol) was massed into a 40 mL reaction vial with pressure relieving septa cap equipped with a stir bar which was preliminarily flushed with argon for 15 minutes. After the addition of 1,2-bis(dicyclohexylphosphino)ethane, the reaction vial was flushed with argon for an additional 10 minutes. Acetonitrile (1 mL) and 1-bromodecane (0.22 mL, 0.23 g, 1.0 mmol) were added to the reaction vial via needle and syringe. The reaction vial was placed in an aluminum heating block which was preheated to 72 ^{o}C and vented by puncturing the cap with a needle twice to relieve pressure buildup. The reaction vial was stirred at 72 ^{o}C for 24 hours. Within 45 minutes, the reaction contents changed from a slurry to a clear solution and remained as a solution through the entire reaction time. At the end of 24 hours, the reaction vial was removed from heat and cooled to room temperature. All volatiles were removed by rotary evaporation. The resulting white gel was dissolved in (approx. 2 mL) chloroform-*d* layered with (approx. 30 mL) hexanes and place in a -20 ˚C freezer. The resulting white powder was isolated by vacuum filtration, resulting in the product as a white solid. (0.126 g, 29.2 %); ^{1}H NMR ($CDCl_3$, 500 MHz), δ 3.09 (d, *J* = 5.0 Hz, 4H) 3.02 – 3.00 (m, 4H) 2.61 – 2.57 (m, 4H) 2.04 – 1.74 (m, 18H) 1.56 – 1.45 (m, 22H) 1.28 – 1.20 (m, 26H) 0.81 (t, *J* = 5.0 Hz, 6H) ; ^{13}C{^{1}H} NMR ($CDCl_3$, 125.8 MHz) δ 31.82, 30.99 (t, *J* = 6.3 Hz) 30.60 (t, *J* = 21.4) 29.46, 29.33, 29.26, 29.10, 27.05, 26.03 (t, *J* = 6.3 Hz), 25.38, 22.65, 22.63, 16.38, 14.08, 11.68 ; ^{31}P{^{1}H} NMR ($CDCl_3$, 202.5 MHz*)*, δ 36.94. HRMS (ESI+): Found 352.3243, expected m/z $C_{46}H_{90}P_2$ [M-2Br]$^{2+}$ 352.3254.

Preparation of ^{Cy}P2P-11,11-Br

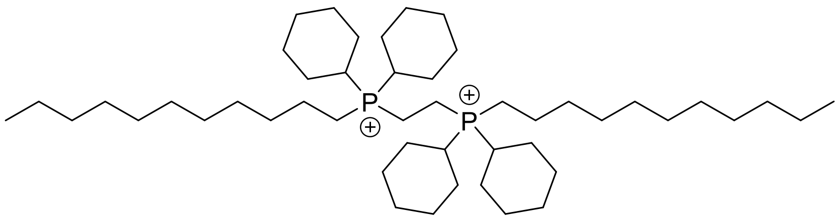

1,2-Bis(dicyclohexylphosphino)ethane (0.211 g, 0.5 mmol) was massed into a 40 mL reaction vial with pressure relieving septa cap equipped with a stir bar which was preliminarily flushed with argon for 15 minutes. After the addition of 1,2-bis(dicyclohexylphosphino)ethane, the reaction vial was flushed with argon for an additional 10 minutes. Acetonitrile (1 mL) and 1-bromoundecane (0.23 mL, 0.25 g, 1.0 mmol) were added to the reaction vial via needle and syringe. The reaction vial was placed in an aluminum heating block which was preheated to 72 ^{o}C vented by puncturing the cap with a needle twice to relieve pressure buildup. The reaction vial was stirred at 72 ^{o}C for 24 hours. Within 45 minutes, the reaction contents changed from a slurry to a clear solution and remained as a solution through the entire reaction time. At the end of 24 hours, the reaction vial was removed from heat and cooled to room temperature. All volatiles were removed by rotary evaporation. The resulting white gel was dissolved in (approx. 2 mL) dichloromethane layered with (approx. 30 mL) hexanes and place in a -20 ˚C freezer. After five days, the mother liquor was decanted from the oil that formed on the bottom of the vial. Residual solvent was again removed by rotary evaporation, resulting in the product as a white, glassy solid. (0.324 g, 72.5 %); ^{1}H NMR ($CDCl_3$, 400 MHz), δ 3.12 (d, *J* = 5.5 Hz, 4H) 3.06 - 2.96 (m, 4H) 2.62 – 2.54 (m, 4H) 2.04 – 1.73 (m, 20H) 1.59 – 1.40 (m, 24H) 1.30 – 1.13 (m, 32H) 0.80 (t, *J* = 6.8 Hz, 6H) ; ^{13}C{^{1}H} NMR ($CDCl_3$, 100.6 MHz) δ 31.91, 31.07 (t, *J* = 8.8 Hz) 30.67 (t, *J* = 25.1) 29.62, 29.56, 29.40, 29.33, 29.17, 27.12, 26.10 (t, *J* = 7.5 Hz), 25.43, 22.77, 22.71, 16.52, 14.16, 11.86 ^{31}P{^{1}H} NMR ($CDCl_3$, 162.0 MHz*)*, δ 36.87. HRMS (ESI+): Found 366.3384, expected m/z $C_{48}H_{94}P_2$ [M-2Br]$^{2+}$ 366.3410.

Preparation of ^{Cy}P2P-12,12-Br

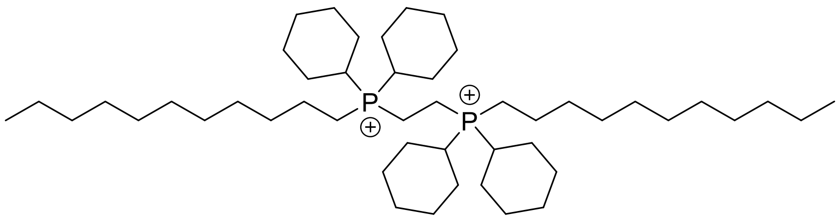

1,2-Bis(dicyclohexylphosphino)ethane (0.211 g, 0.5 mmol) was massed into a 40 mL reaction vial with pressure relieving septa cap equipped with a stir bar which was preliminarily flushed with argon for 15 minutes. After the addition of 1,2-bis(dicyclohexylphosphino)ethane, the reaction vial was flushed with argon for an additional 10 minutes. Acetonitrile (1 mL) and 1-bromododecane (0.25 mL, 0.26 g, 1.0 mmol) were added to the reaction vial via needle and syringe. The reaction vial was placed in an aluminum heating block which was preheated to 72 ^{o}C and vented by puncturing the cap with a needle twice to relieve pressure buildup. The reaction vial was stirred at 72 ^{o}C for 24 hours. Within 45 minutes, the reaction contents changed from a slurry to a clear solution and remained as a solution through the entire reaction time. At the end of 24 hours, the reaction vial was removed from heat and cooled to room temperature. All volatiles were removed by rotary evaporation. The resulting white gel was dissolved in (approx. 2 mL) dichloromethane layered with (approx. 30 mL) hexanes and place in a -20 ˚C freezer. After two days, the mother liquor was decanted from the oil that formed on the bottom of the vial. Residual solvent was again removed by rotary evaporation, resulting in the product as a white, glassy solid. (0.334 g, 72.5 %); ^{1}H NMR ($CDCl_3$, 400 MHz), δ 3.11 (d, *J* = 4 Hz, 4H) 3.07 - 2.97 (m, 4H) 2.62 – 2.55 (m, 4H) 2.05 – 1.74 (m, 20H) 1.60 – 1.40 (m, 24H) 1.32 – 1.16 (m, 36H) 0.82 (t, *J* = 4 Hz, 6H) ; ^{13}C{^{1}H} NMR ($CDCl_3$, 100.6 MHz) δ 31.94, 31.08 (t, *J* = 8.8 Hz) 30.68 (t, *J* = 25.1) 29.68, 29.64, 29.58, 29.41, 29.37, 29.18, 27.12, 26.11 (t, *J* = 6.0 Hz), 25.44, 22.74, 22.72, 16.30, 14.17, 11.64 ^{31}P{^{1}H} NMR ($CDCl_3$, 162.0 MHz), δ 36.88. HRMS (ESI+): Found 380.3553, expected m/z $C_{50}H_{98}P_2$ [M-2Br]$^{2+}$ 380.3567.

Preparation of ^{Cy}P2P-13,13-Br

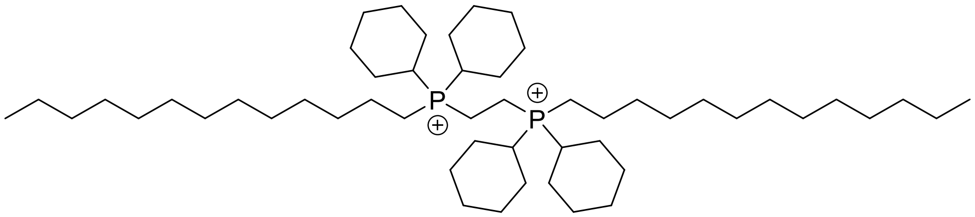

1,2-Bis(dicyclohexylphosphino)ethane (0.211 g, 0.5 mmol) was massed into a 40 mL reaction vial with pressure relieving septa cap equipped with a stir bar which was preliminarily flushed with argon for 15 minutes. After the addition of 1,2-bis(dicyclohexylphosphino)ethane, the reaction vial was flushed with argon for an additional 10 minutes. Acetonitrile (1 mL) and 1-bromotridecane (0.27 mL, 0.28 g, 1.0 mmol) were added to the reaction vial via needle and syringe. The reaction vial was placed in an aluminum heating block which was preheated to 72 ^{o}C and vented by puncturing the cap with a needle twice to relieve pressure buildup. The reaction vial was stirred at 72 ^{o}C for 24 hours. Within 45 minutes, the reaction contents changed from a slurry to a clear solution and remained as a solution through the entire reaction time. At the end of 24 hours, the reaction vial was removed from heat and cooled to room temperature. All volatiles were removed by rotary evaporation. The resulting white gel was washed with diethyl ether. Residual solvent was again removed by rotary evaporation, resulting in the product as a white, glassy solid. (0.401 g, 84.6 %); ^{1}H NMR ($CDCl_3$, 500 MHz), δ 3.18 (d, *J* = 5.0 Hz, 4H) 3.11 – 3.02 (m, 4H) 2.67 – 2.62 (m, 4H) 2.10 – 1.74 (m, 26H) 1.62 – 1.48 (m, 24H) 1.34 – 1.24 (m, 42H) 0.87 (t, *J* = 5.0 Hz, 6H) ; ^{13}C{^{1}H} NMR ($CDCl_3$, 125.8 MHz) δ 32.04, 31.16 (t, *J* = 7.5 Hz) 30.78 (t, *J* = 20.2) 29.78, 29.76, 29.67, 29.50, 29.47, 29.27, 27.21, 26.20 (t, *J* = 6.3 Hz), 25.53, 22.86, 22.81, 16.63, 14.25, 11.82 ^{31}P{^{1}H} NMR ($CDCl_3$, 202.5 MHz*)*, δ 36.88. HRMS (ESI+): Found 380.3553, expected m/z $C_{50}H_{98}P_2$ $[M\text{-}2Br]^{2+}$ 380.3567.HRMS (ESI+): Found 394.3693, expected m/z $C_{52}H_{102}P_2$ $[M\text{-}2Br]^{2+}$ 394.3723.

Preparation of ^{Cy}P2P-14,14-Br

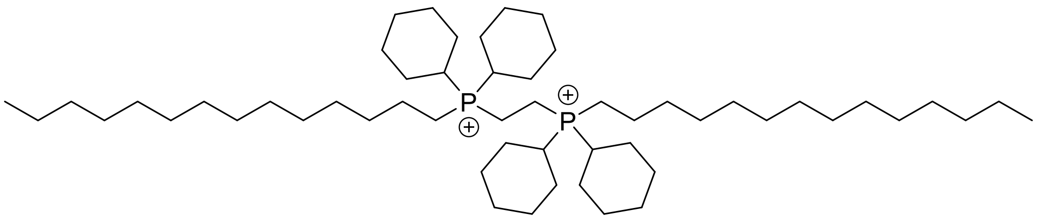

1,2-Bis(dicyclohexylphosphino)ethane (0.211 g, 0.5 mmol) was massed into a 40 mL reaction vial with pressure relieving septa cap equipped with a stir bar which was preliminarily flushed with argon for 15 minutes. After the addition of 1,2-bis(dicyclohexylphosphino)ethane, the reaction vial was flushed with argon for an additional 10 minutes. Acetonitrile (1 mL) and 1-bromotetradecane (0.31 mL, 0.29 g, 1.0 mmol) were added to the reaction vial via needle and syringe. The reaction vial was placed in an aluminum heating block which was preheated to 72 ^{o}C and vented by puncturing the cap with a needle twice to relieve pressure buildup. The reaction vial was stirred at 72 ^{o}C for 24 hours. Within 45 minutes, the reaction contents changed from a slurry to a clear solution and remained as a solution through the entire reaction time. At the end of 24 hours, the reaction vial was removed from heat and cooled to room temperature. All volatiles were removed by rotary evaporation. The resulting white gel was dissolved in (approx. 2 mL) dichloromethane layered with (approx. 30 mL) hexanes and place in a -20 ˚C freezer. After two days, the mother liquor was decanted from the oil that formed on the bottom of the vial. Residual solvent was again removed by rotary evaporation, resulting in the product as a white, glassy solid. (0.379 g, 77.6 %); ^{1}H NMR ($CDCl_3$, 500 MHz), δ 3.12 (d, *J* = 4.0, 4H) 3.06 – 2.98 (m, 4H) 2.62 – 2.56 (m, 4H) 2.05 – 1.75 (m, 20H) 1.57 – 1.43 (m, 24H) 1.30 – 1.20 (m, 46H) 0.82 (t, *J* = 4.0, 6H) ; ^{13}C{^{1}H} NMR ($CDCl_3$, 125.8 MHz) δ 31.92, 31.03 (t, *J* = 8.8 Hz) 30.65 (t, *J* = 25.1) 29.68, 29.66, 29.65, 29.55, 29.38, 29.36, 29.14, 27.09, 26.07 (t, *J* = 6.0 Hz), 25.41, 22.72, 22.69, 16.47, 14.11, 11.80 ^{31}P{^{1}H} NMR ($CDCl_3$, 202.5 MHz*)*, δ 36.89. HRMS (ESI+): Found 408.3862, expected m/z $C_{54}H_{106}P_2$ $[M\text{-}2Br]^{2+}$ 408.3880.

Preparation of ^{Cy}P2P-16,16-Br

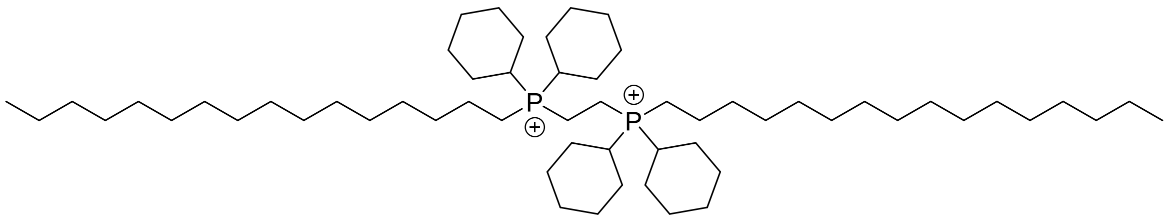

1,2-Bis(dicyclohexylphosphino)ethane (0.211 g, 0.5 mmol) was massed into a 40 mL reaction vial with pressure relieving septa cap equipped with a stir bar which was preliminarily flushed with argon for 15 minutes. After the addition of 1,2-bis(dicyclohexylphosphino)ethane, the reaction vial was flushed with argon for an additional 10 minutes. Acetonitrile (1 mL) and 1-bromohexadecane (0.32 mL, 0.32 g, 1.0 mmol) were added to the reaction vial via needle and syringe. The reaction vial was placed in an aluminum heating block which was preheated to 72 ^{o}C and vented by puncturing the cap with a needle twice to relieve pressure buildup. The reaction vial was stirred at 72 ^{o}C for 24 hours. Within 45 minutes, the reaction contents changed from a slurry to a clear solution and remained as a solution through the entire reaction time. At the end of 24 hours, the reaction vial was removed from heat and cooled to room temperature. All volatiles were removed by rotary evaporation. The resulting white gel was dissolved in (approx. 2 mL) dichloromethane layered with (approx. 30 mL) hexanes and place in a -20 ˚C freezer. After five days, the mother liquor was decanted from the oil that formed on the bottom of the vial. Residual solvent was again removed by rotary evaporation. Finally, the white, glassy solid was washed with diethyl ether and isolated by vacuum filtration. (0.377 g, 73.0 %); ^{1}H NMR ($CDCl_3$, 400 MHz), δ 3.10 (d, *J* = 4.0 Hz, 4H) 3.03 – 2.96 (m, 4H) 2.60 – 2.54 (m, 4H) 2.03 – 1.72 (m, 20H) 1.55 – 1.43 (m, 22H) 1.26 – 1.17 (m, 52H) 0.79 (t, *J* = 4.0 Hz, 6H) ; ^{13}C{^{1}H} NMR ($CDCl_3$, 100.6 MHz) δ 31.85, 30.96 (t, *J* = 6.0 Hz) 30.57 (t, *J* = 16.1 Hz) 29.63, 29.61, 29.59, 29.57, 29.47, 29.30, 29.28, 29.06, 26.99, 25.99 (t, *J* = 5.0 Hz), 25.33, 22.64, 22.61, 16.40, 14.05, 11.75 ^{31}P{^{1}H} NMR ($CDCl_3$, 162.0 MHz), δ 36.87. HRMS (ESI+): Found 436.418, expected m/z $C_{58}H_{114}P_2$ $[M-2Br]^{2+}$ 436.419.

II. NMR Spectroscopic Data: ^{1}H, ^{31}P, and ^{13}C NMR

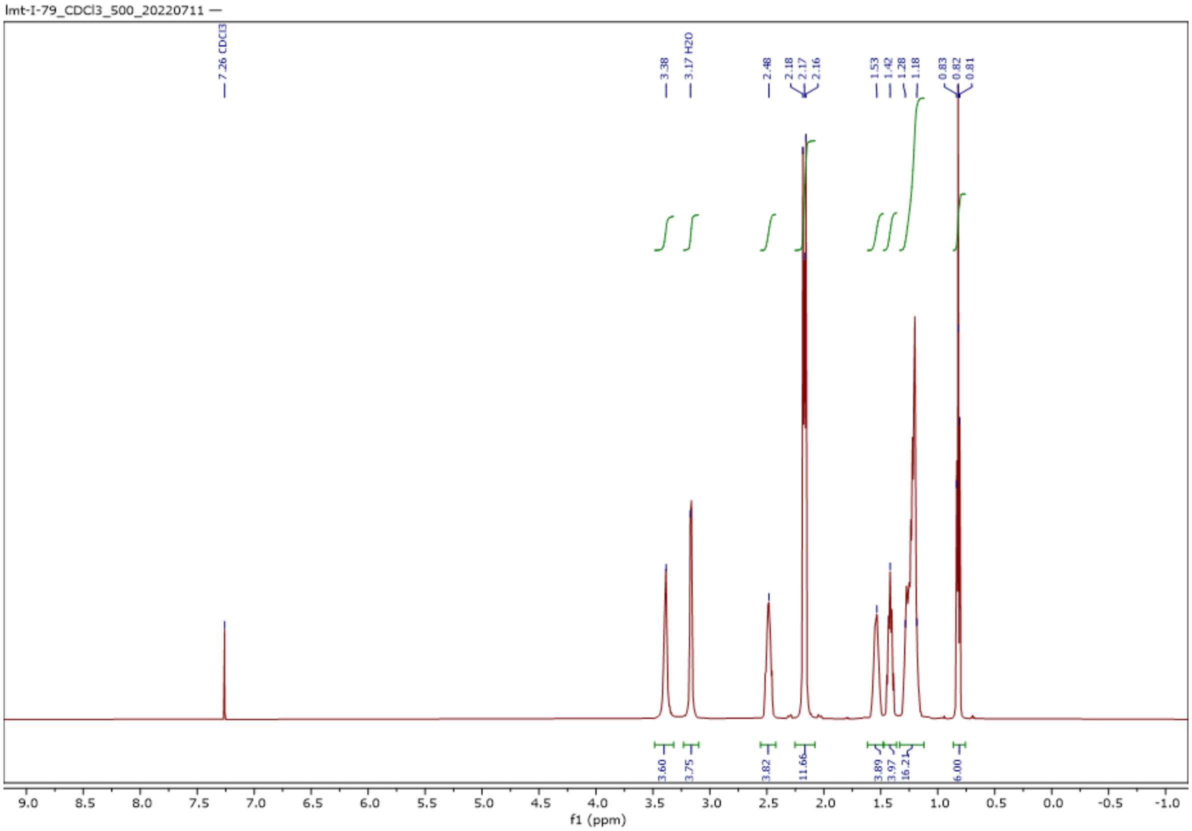

Figure S1. ^{1}H NMR of **^{Me}P2P-8,8-Br** in $CDCl_3$

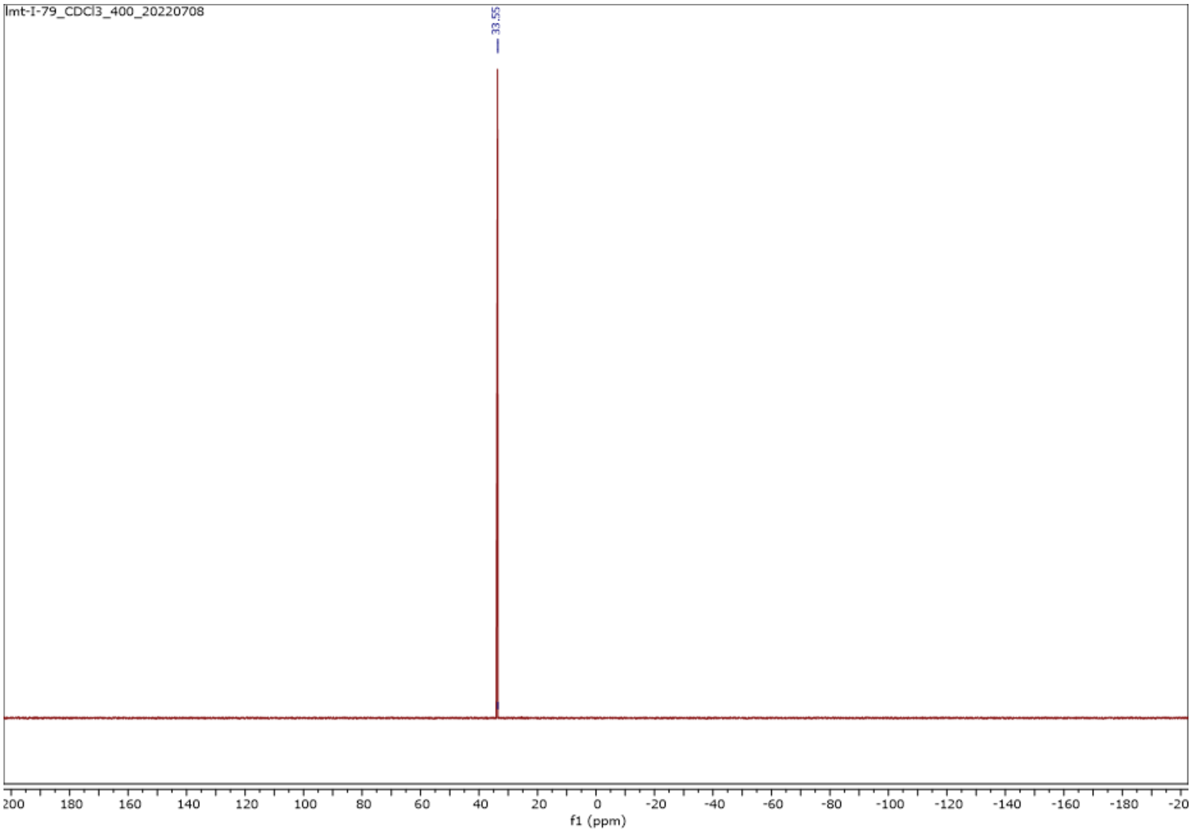

Figure S2. ^{31}P NMR of **^{Me}P2P-8,8-Br** in $CDCl_3$

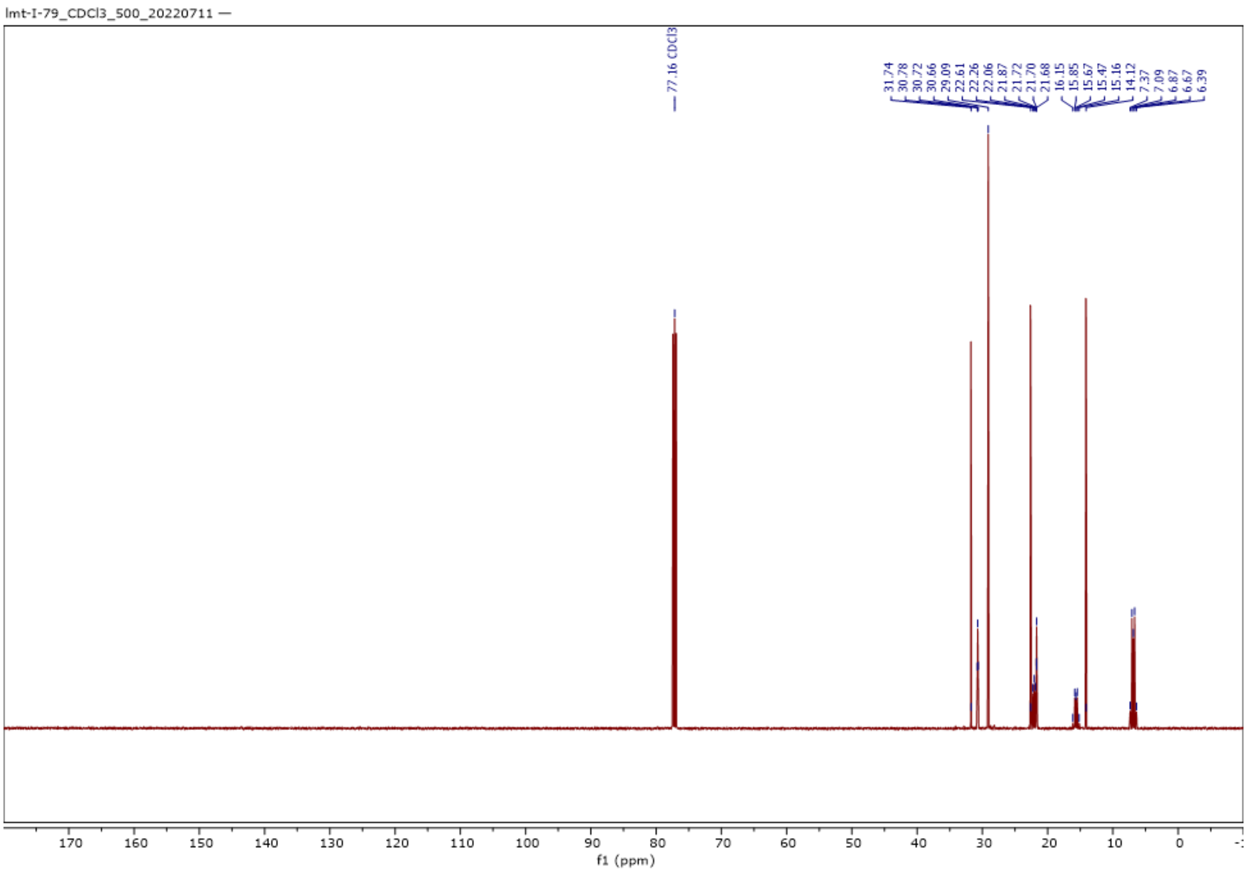

Figure S3. ^{13}C NMR of **^{Me}P2P-8,8-Br** in $CDCl_3$

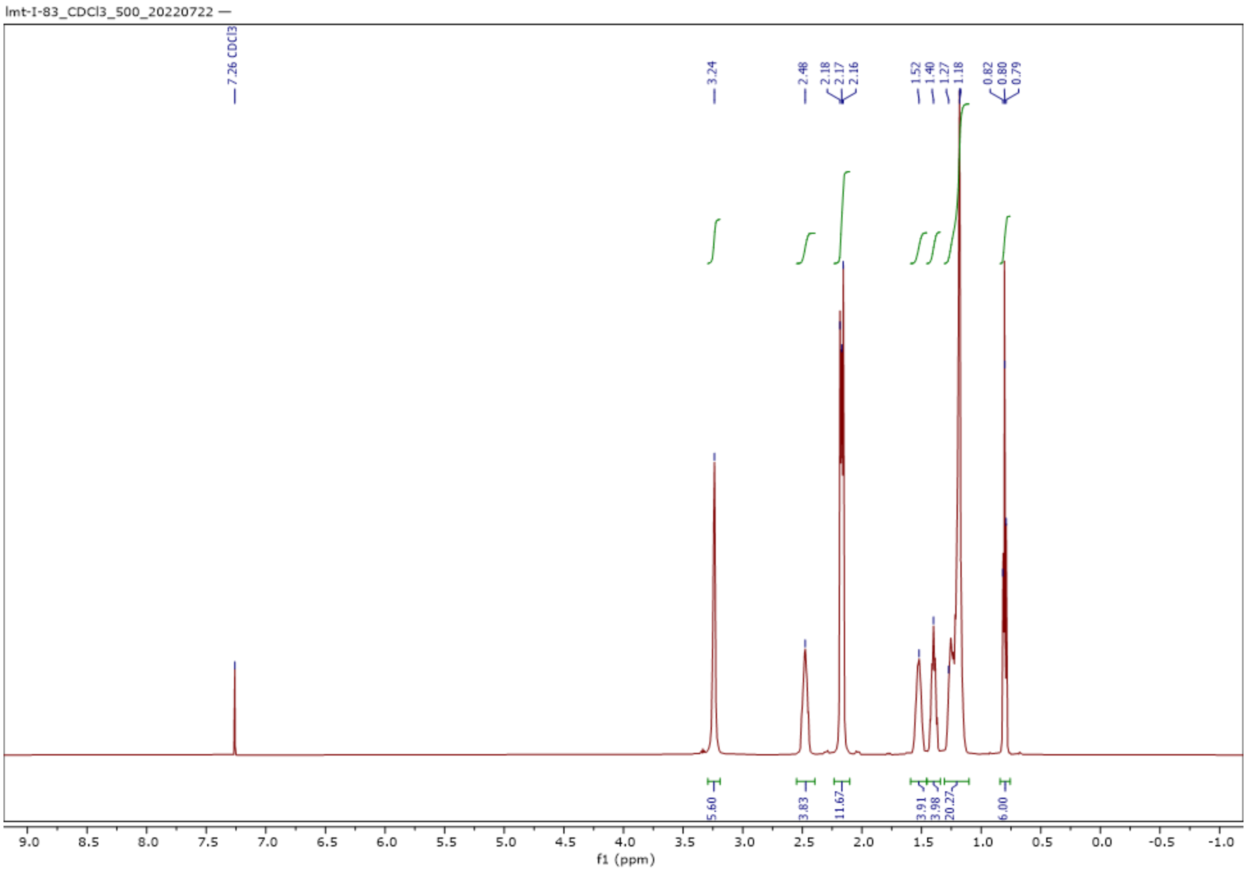

Figure S4. ^{1}H NMR of **^{Me}P2P-9,9-Br** in $CDCl_3$

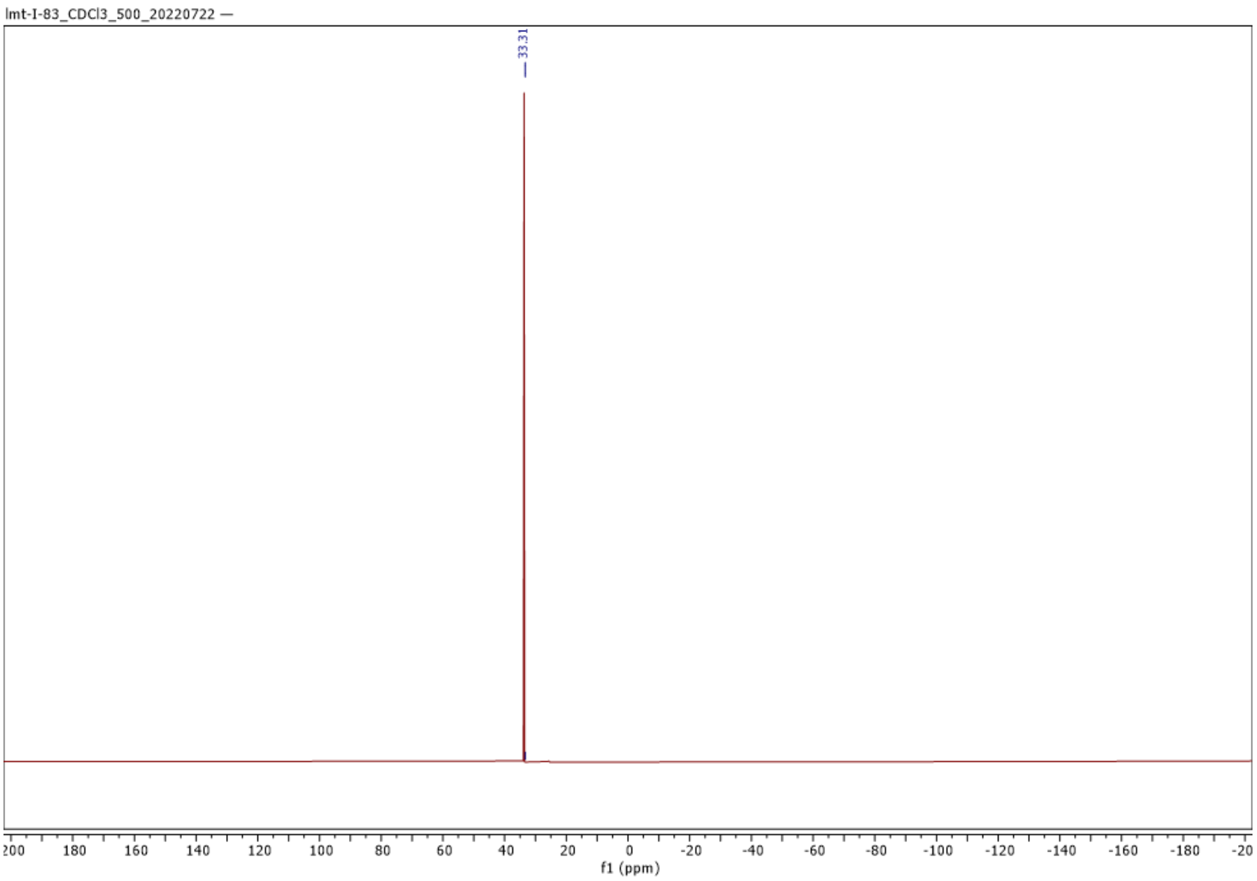

Figure S5. ^{31}P NMR of **^{Me}P2P-9,9-Br** in $CDCl_3$

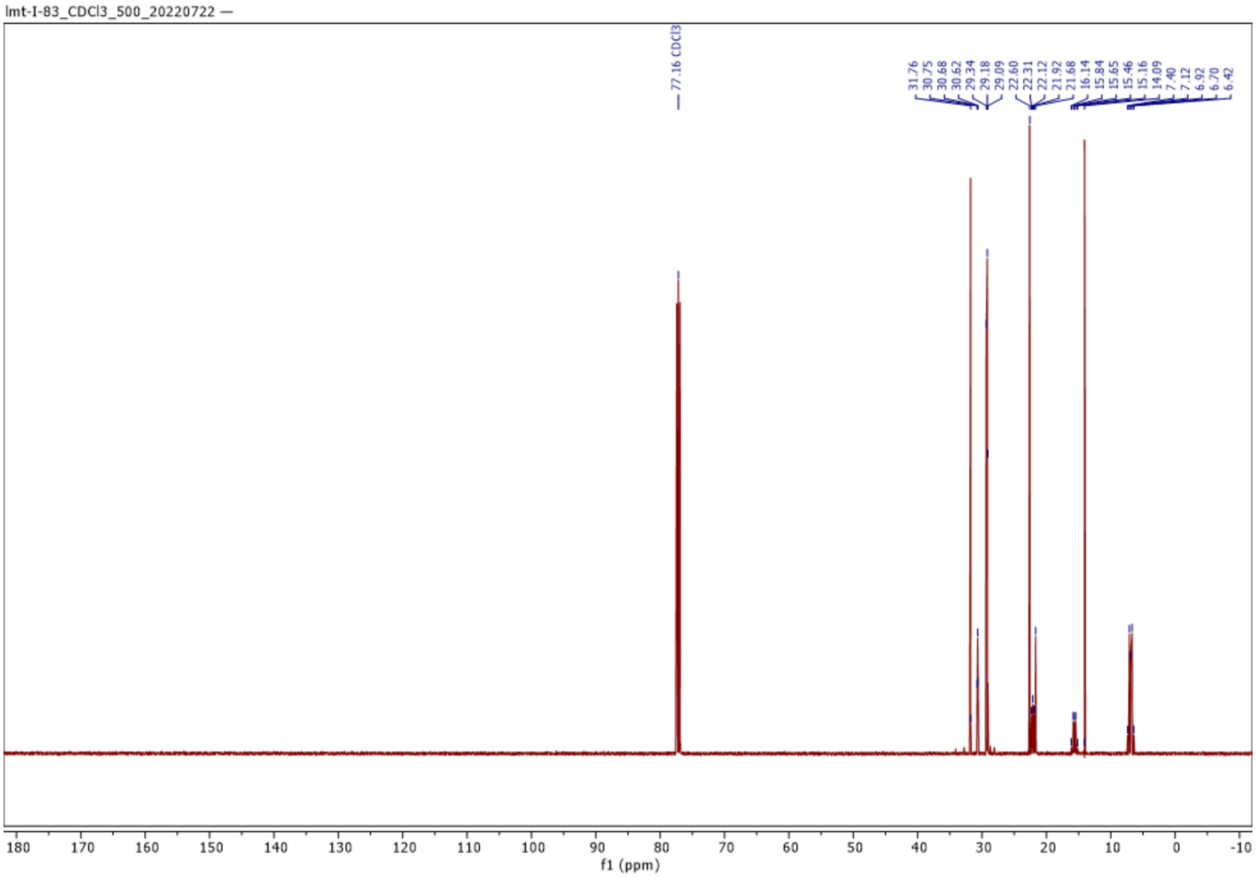

Figure S6. ^{13}C NMR of **^{Me}P2P-9,9-Br** in $CDCl_3$

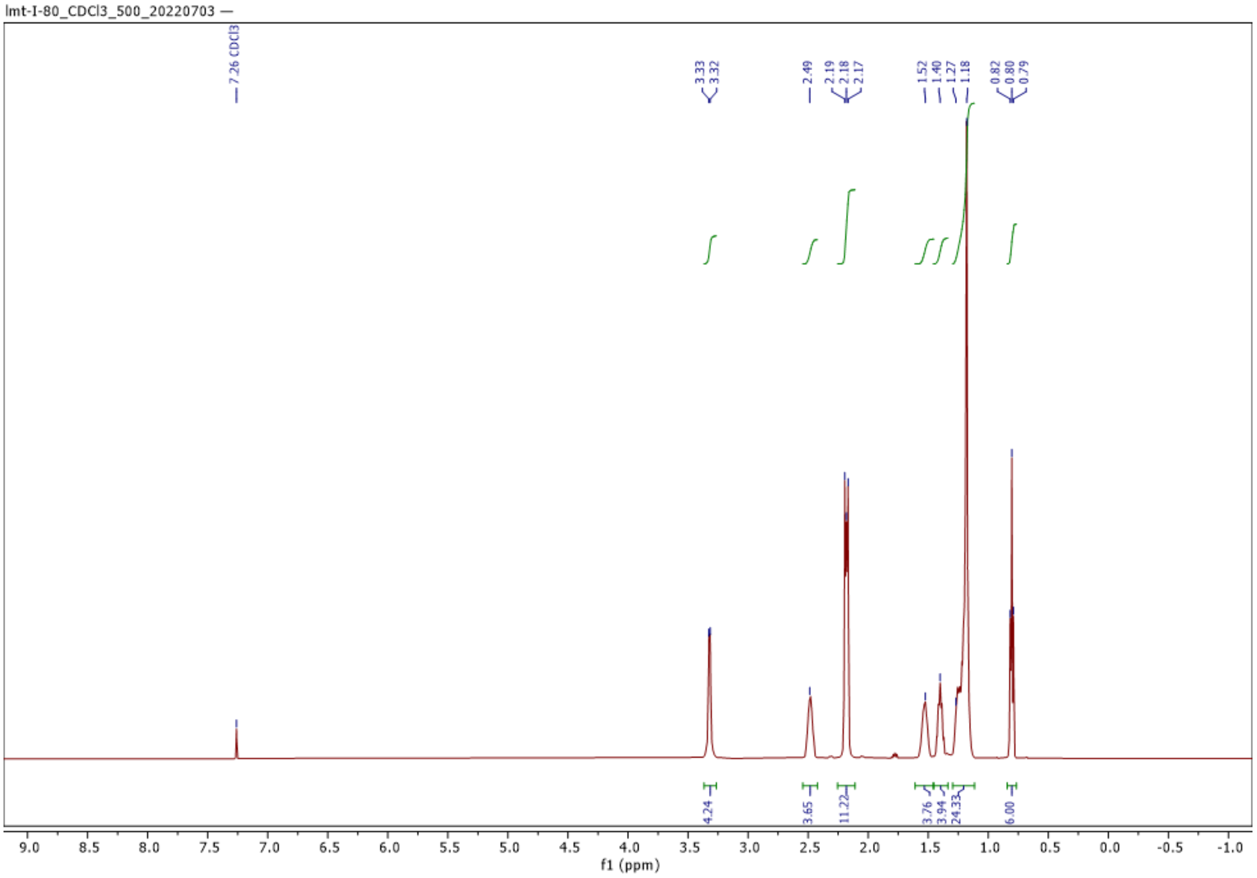

Figure S7. ^{1}H NMR of ^{Me}**P2P-10,10-Br** in $CDCl_3$

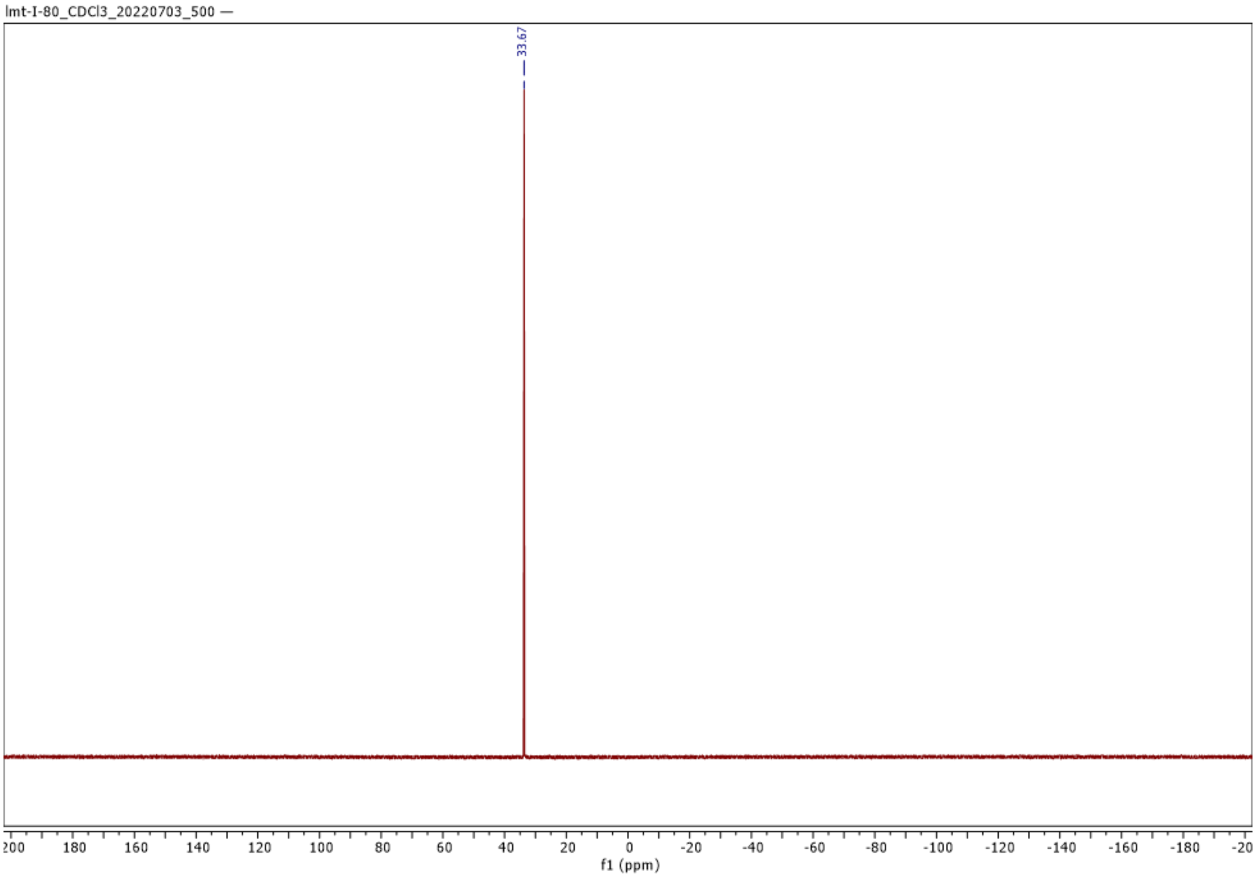

Figure S8. ^{31}P NMR of ^{Me}**P2P-10,10-Br** in $CDCl_3$

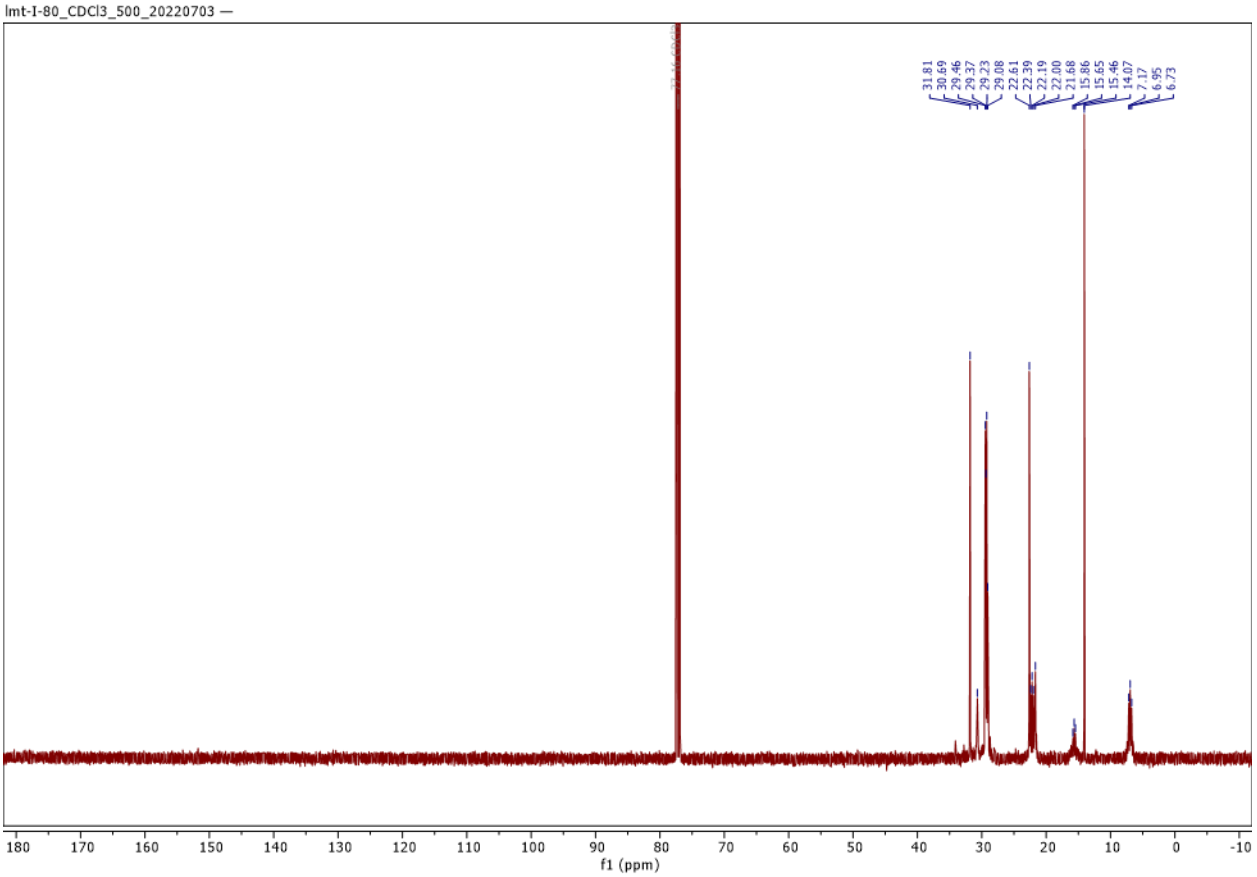

Figure S9. ^{13}C NMR of **^{Me}P2P-10,10-Br** in $CDCl_3$

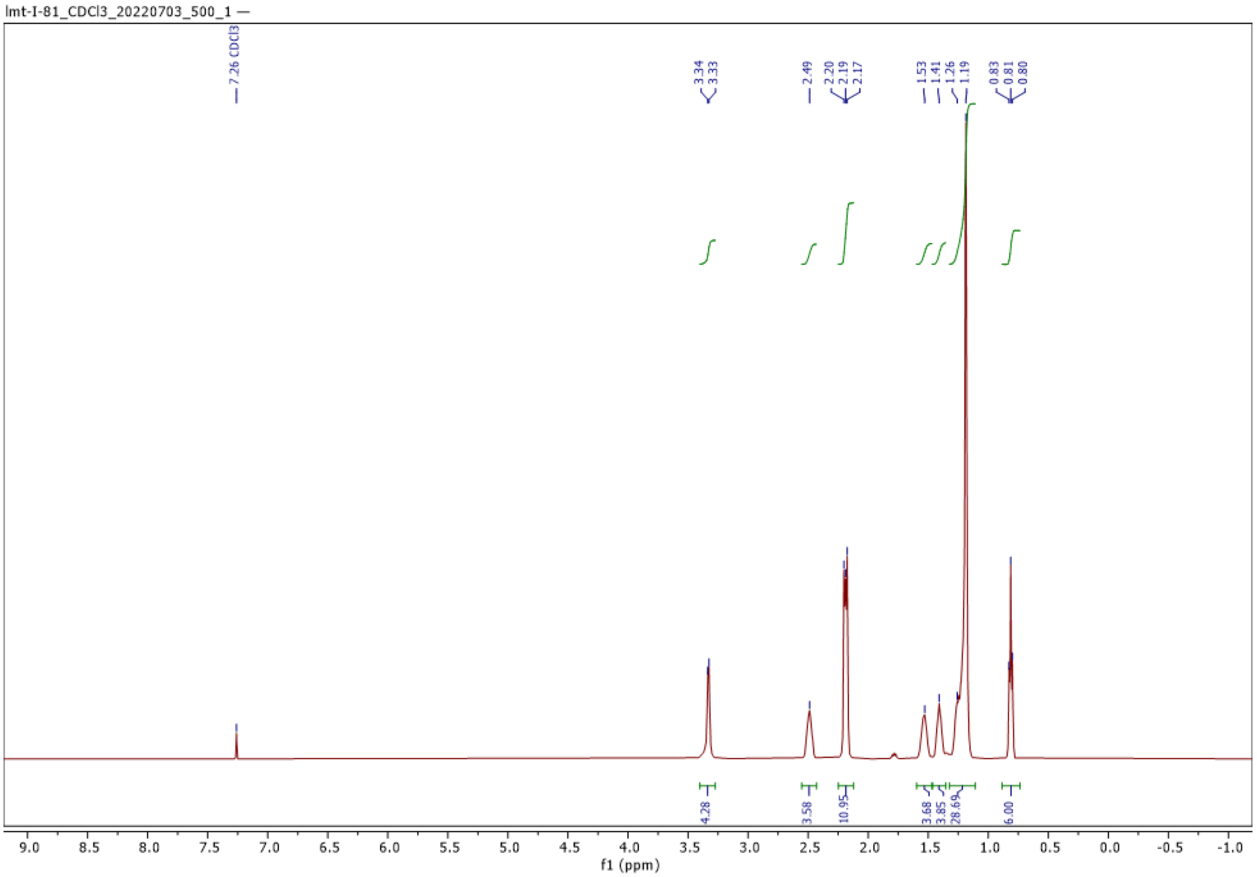

Figure S10. ^{1}H NMR of **^{Me}P2P-11,11-Br** in $CDCl_3$

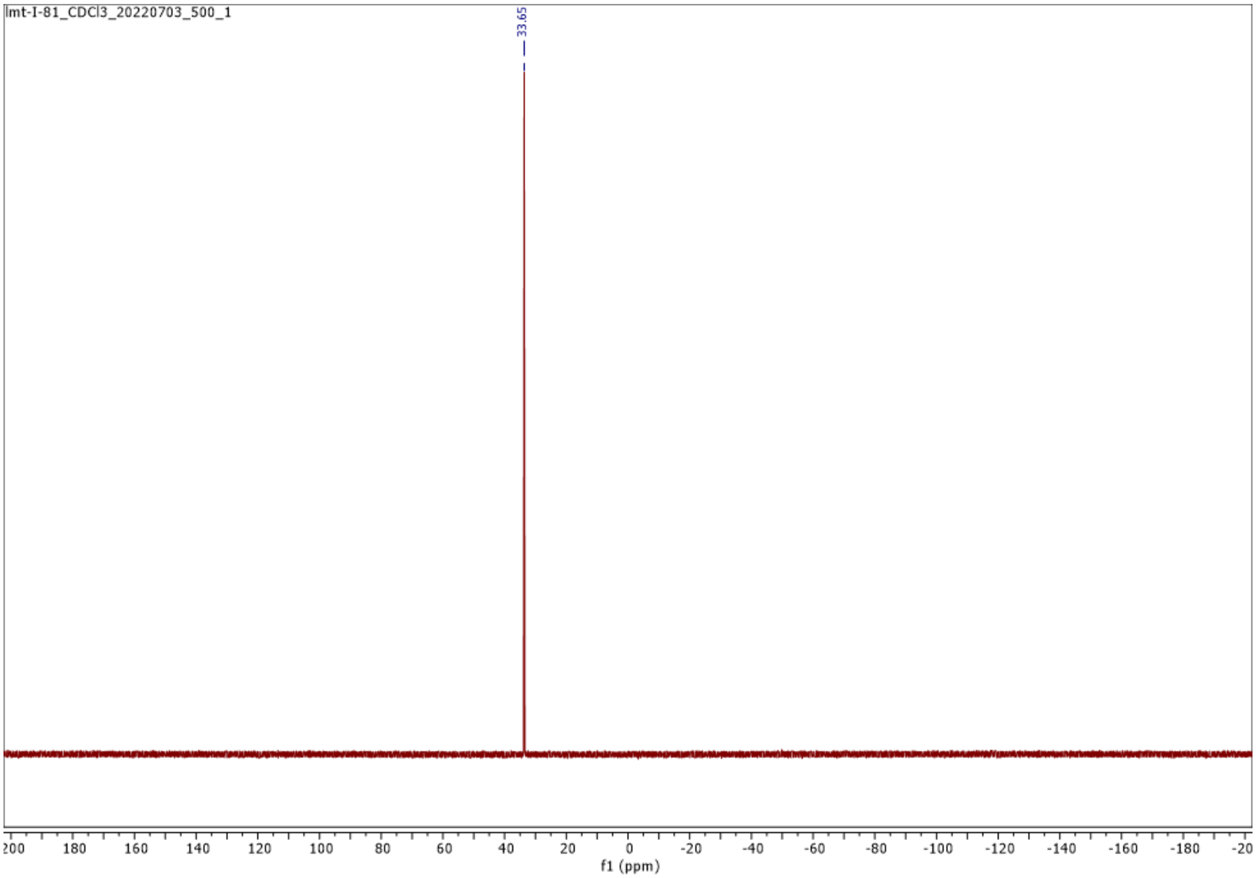

Figure S11. ^{31}P NMR of **^{Me}P2P-11,11-Br** in $CDCl_3$

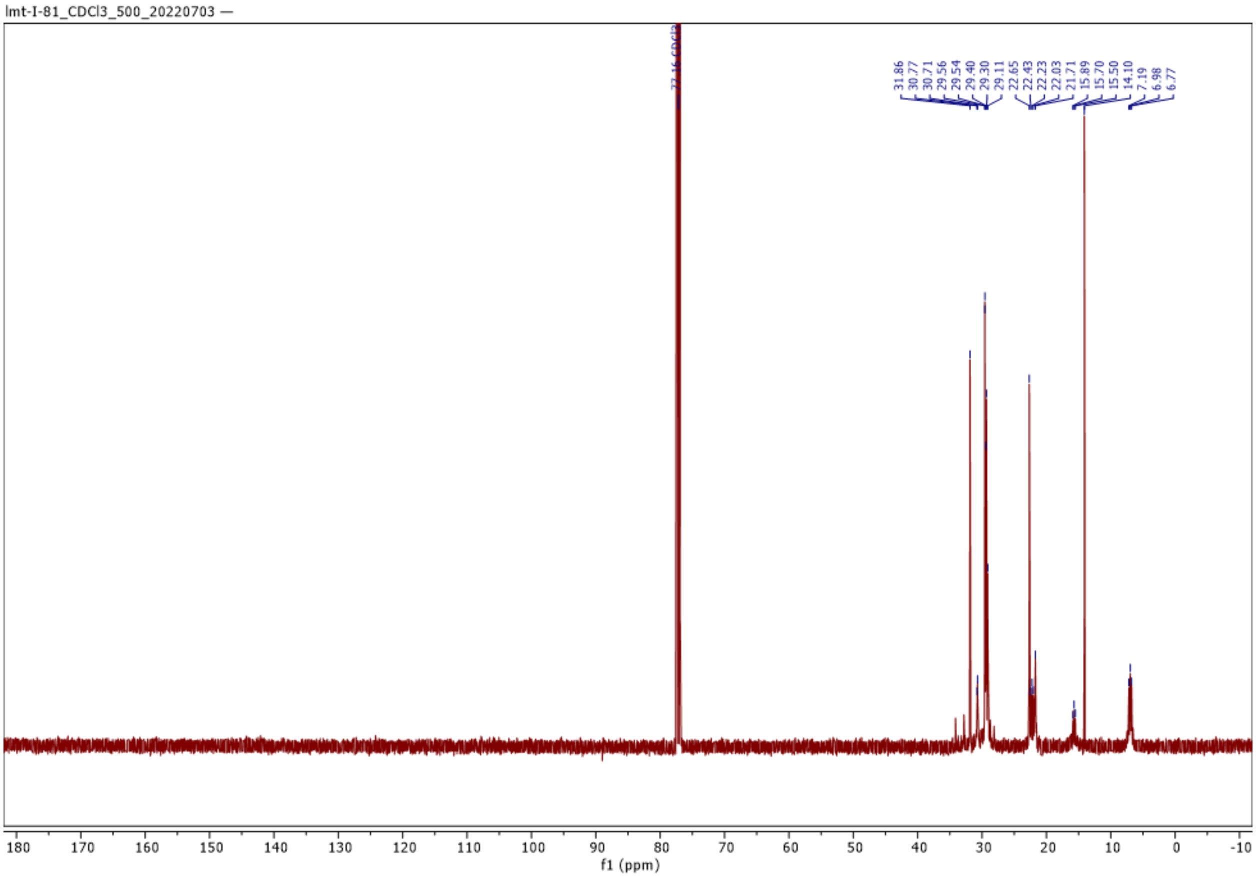

Figure S12. ^{13}C NMR of **^{Me}P2P-11,11-Br** in $CDCl_3$

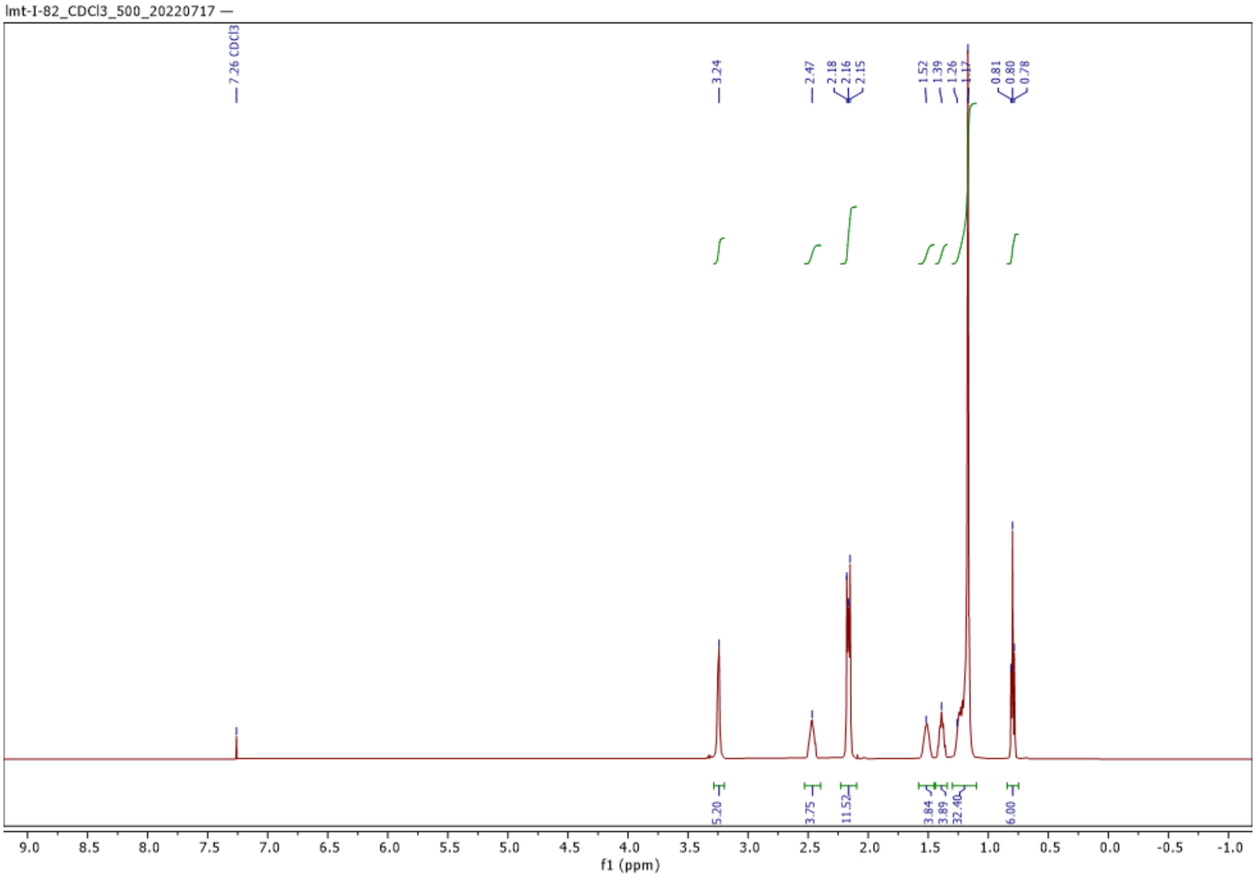

Figure S13. ^{1}H NMR of **^{Me}P2P-12,12-Br** in $CDCl_3$

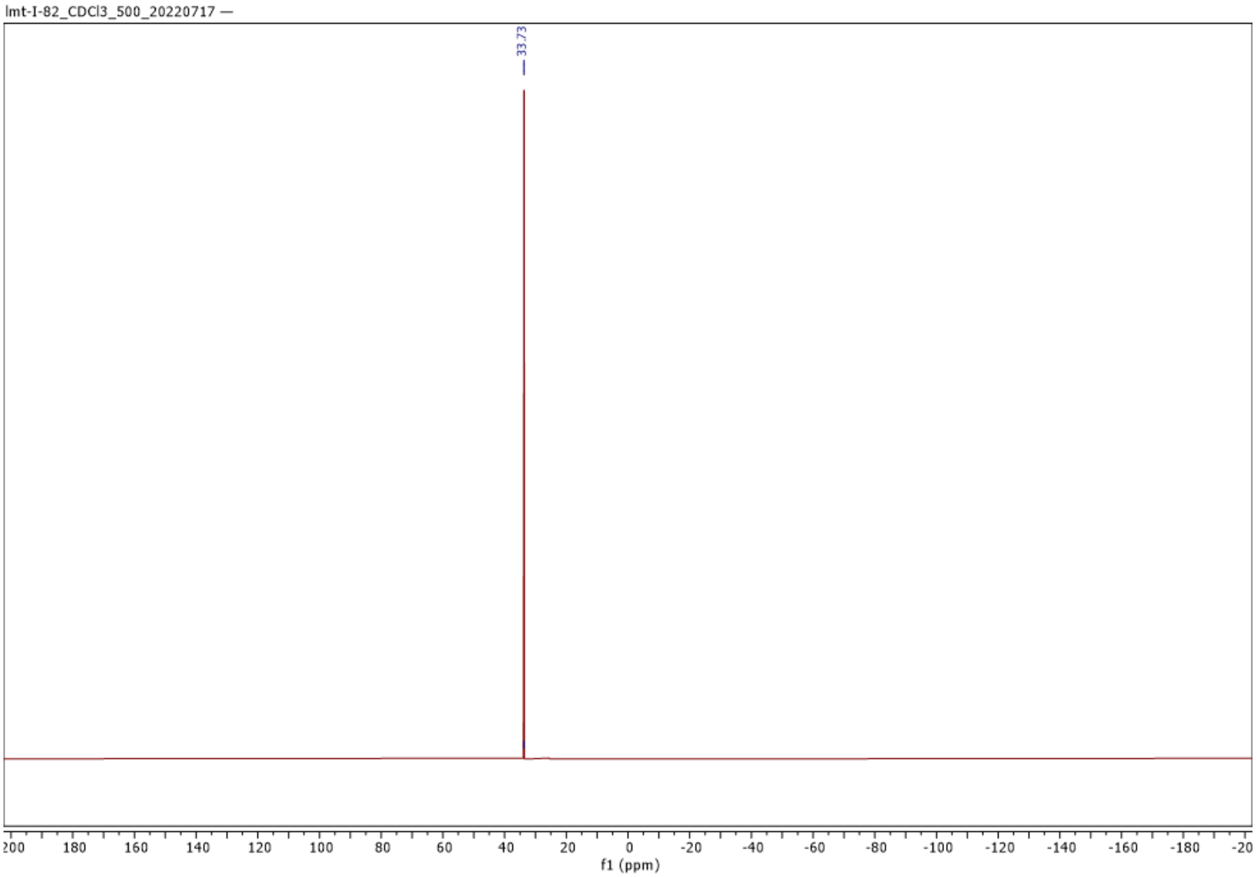

Figure S14. ^{31}P NMR of **^{Me}P2P-12,12-Br** in $CDCl_3$

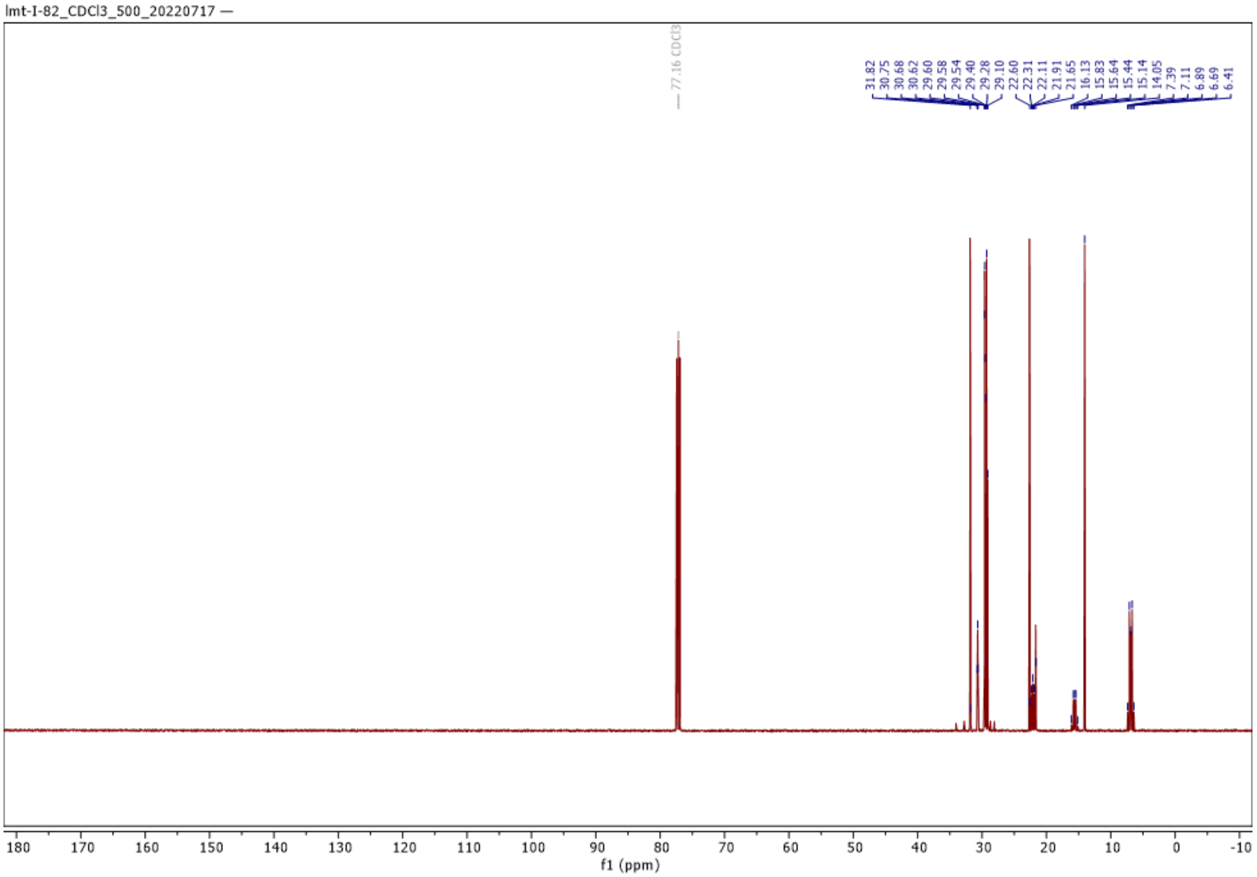

Figure S15. ^{13}C NMR of **^{Me}P2P-12,12-Br** in $CDCl_3$

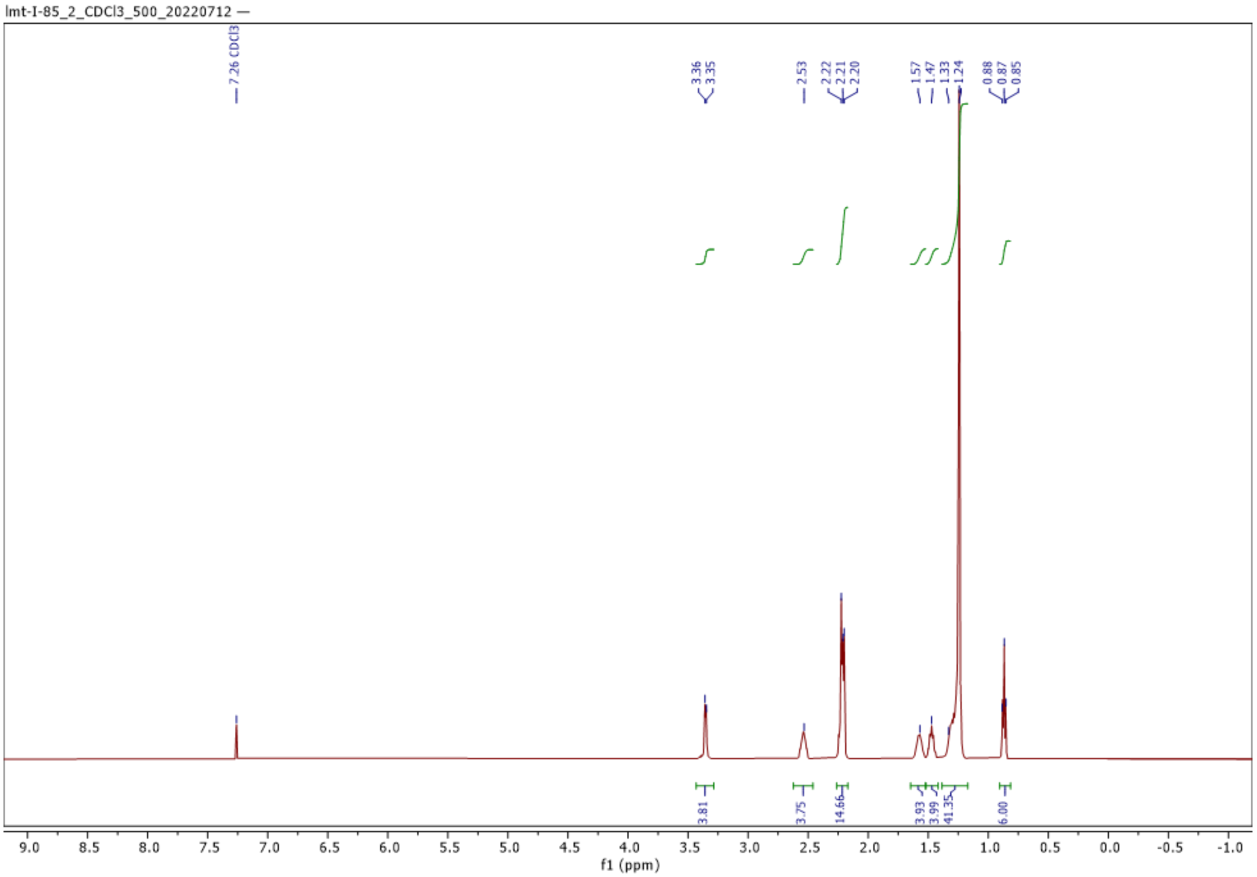

Figure S16. ^{1}H NMR of **^{Me}P2P-14,14-Br** in $CDCl_3$

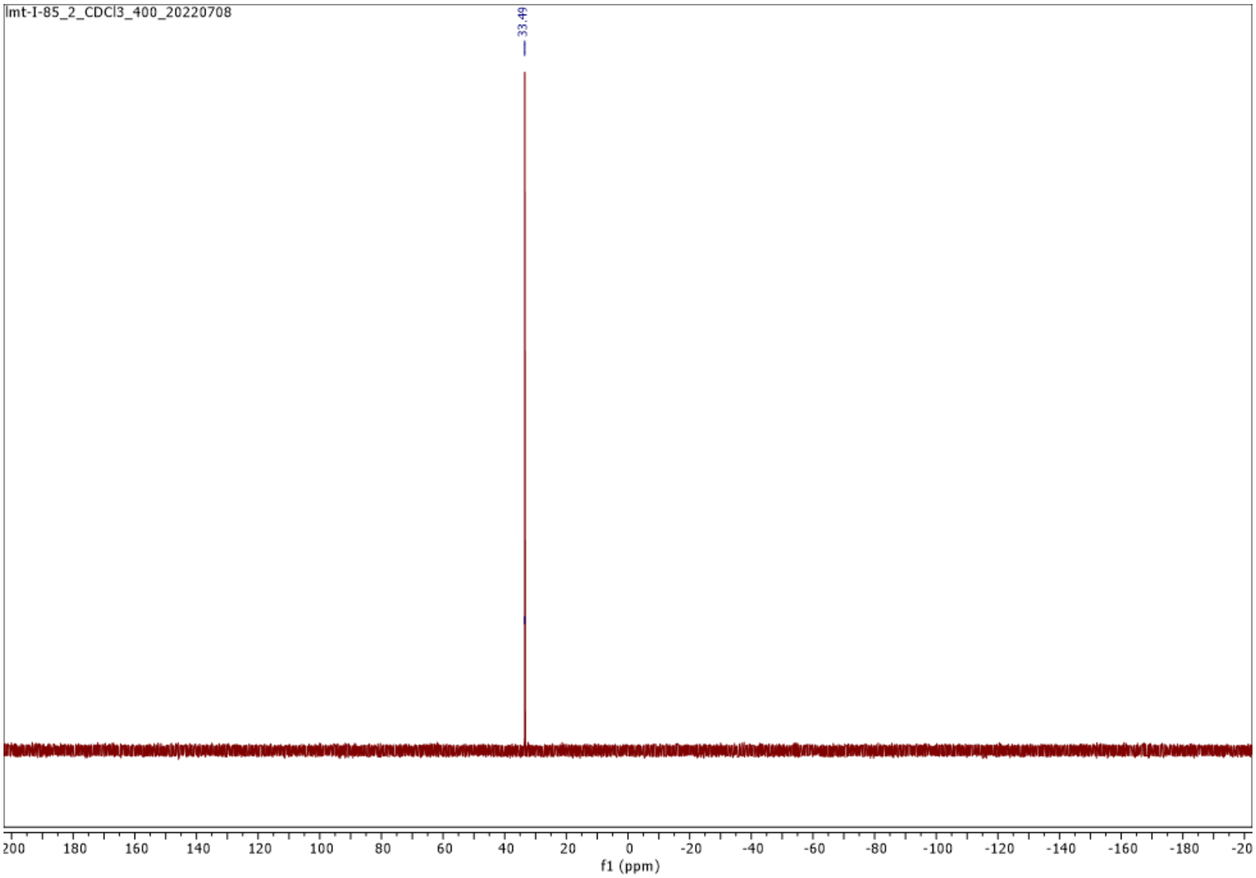

Figure S17. ^{31}P NMR of ^{Me}**P2P-14,14-Br** in $CDCl_3$

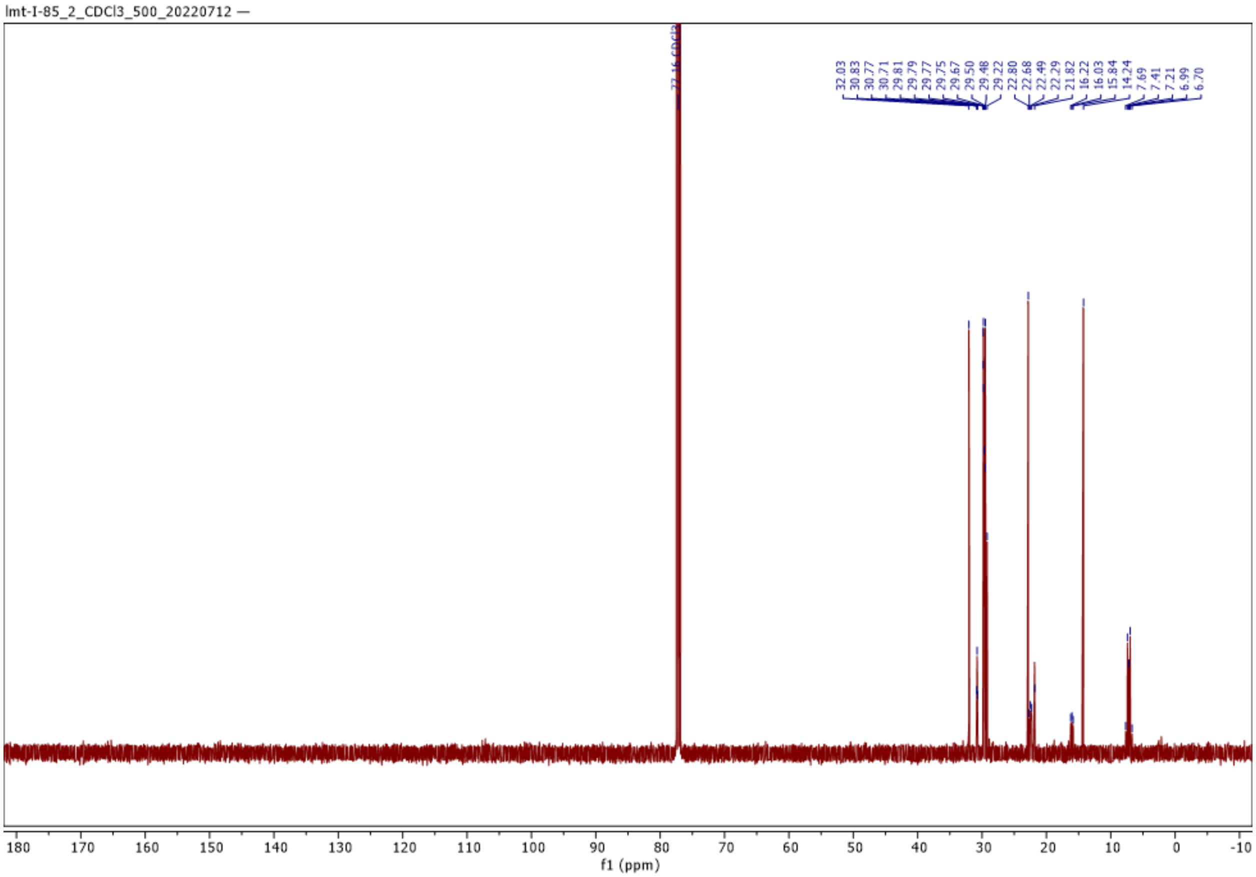

Figure S18. ^{13}C NMR of ^{Me}**P2P-14,14-Br** in $CDCl_3$

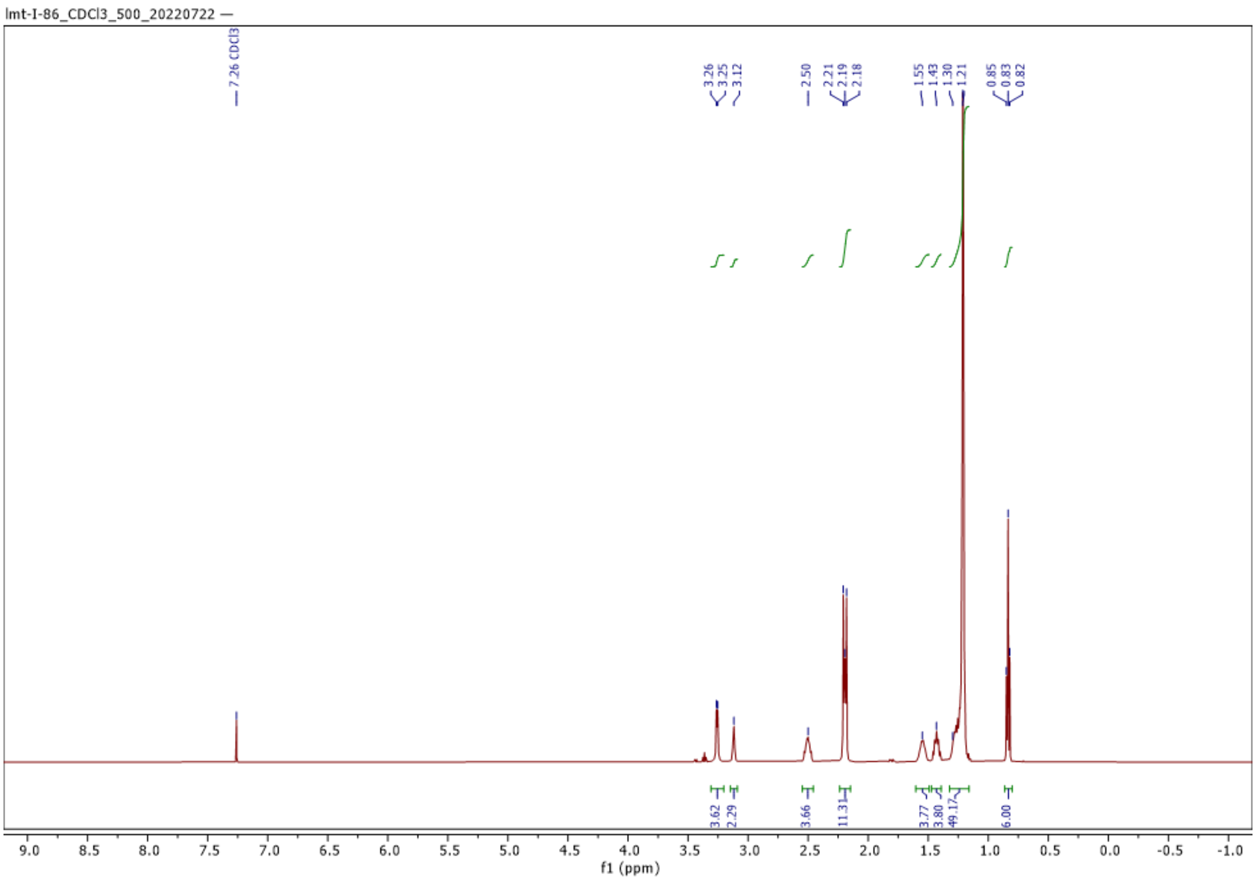

Figure S19. ^{1}H NMR of $^{\mathbf{Me}}$**P2P-16,16-Br** in $CDCl_3$

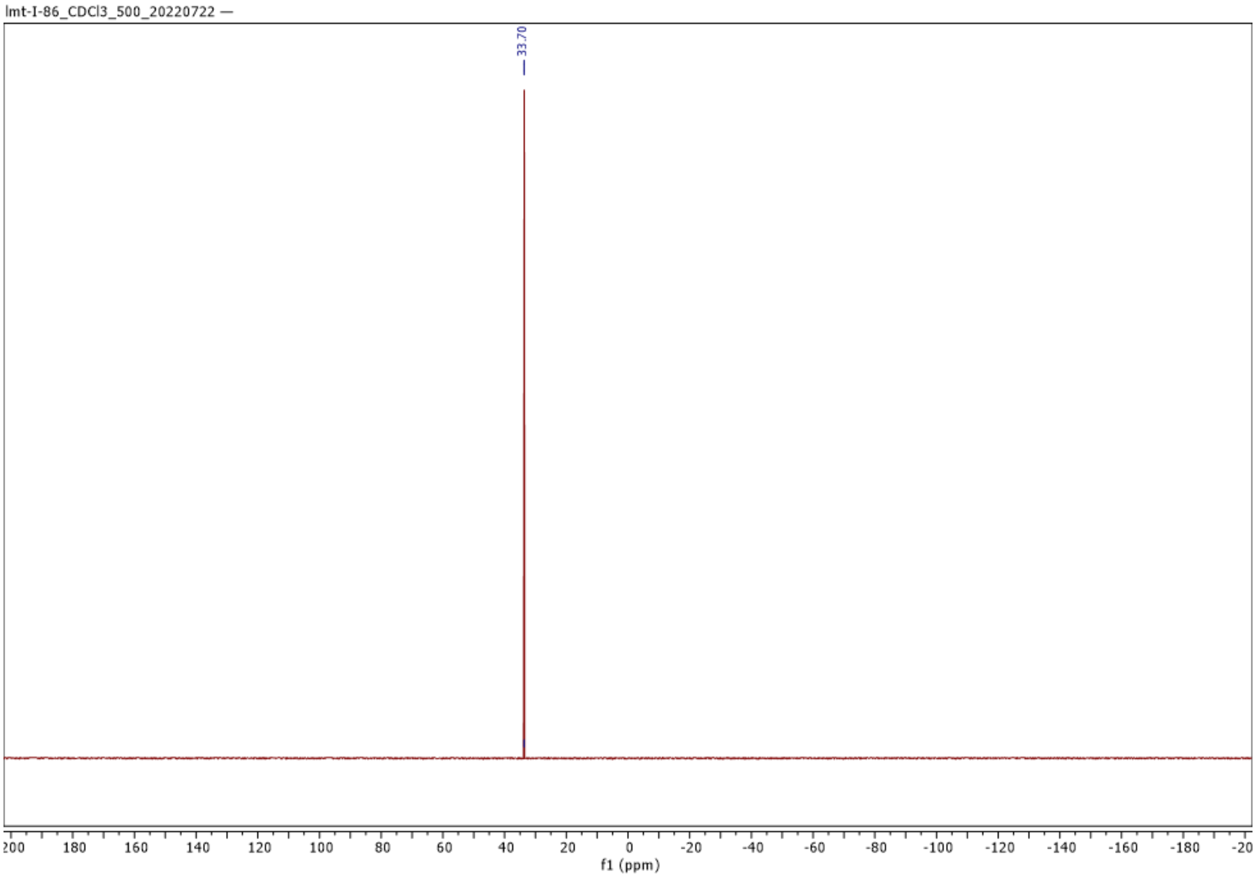

Figure S20. ^{31}P NMR of $^{\mathbf{Me}}$**P2P-16,16-Br** in $CDCl_3$

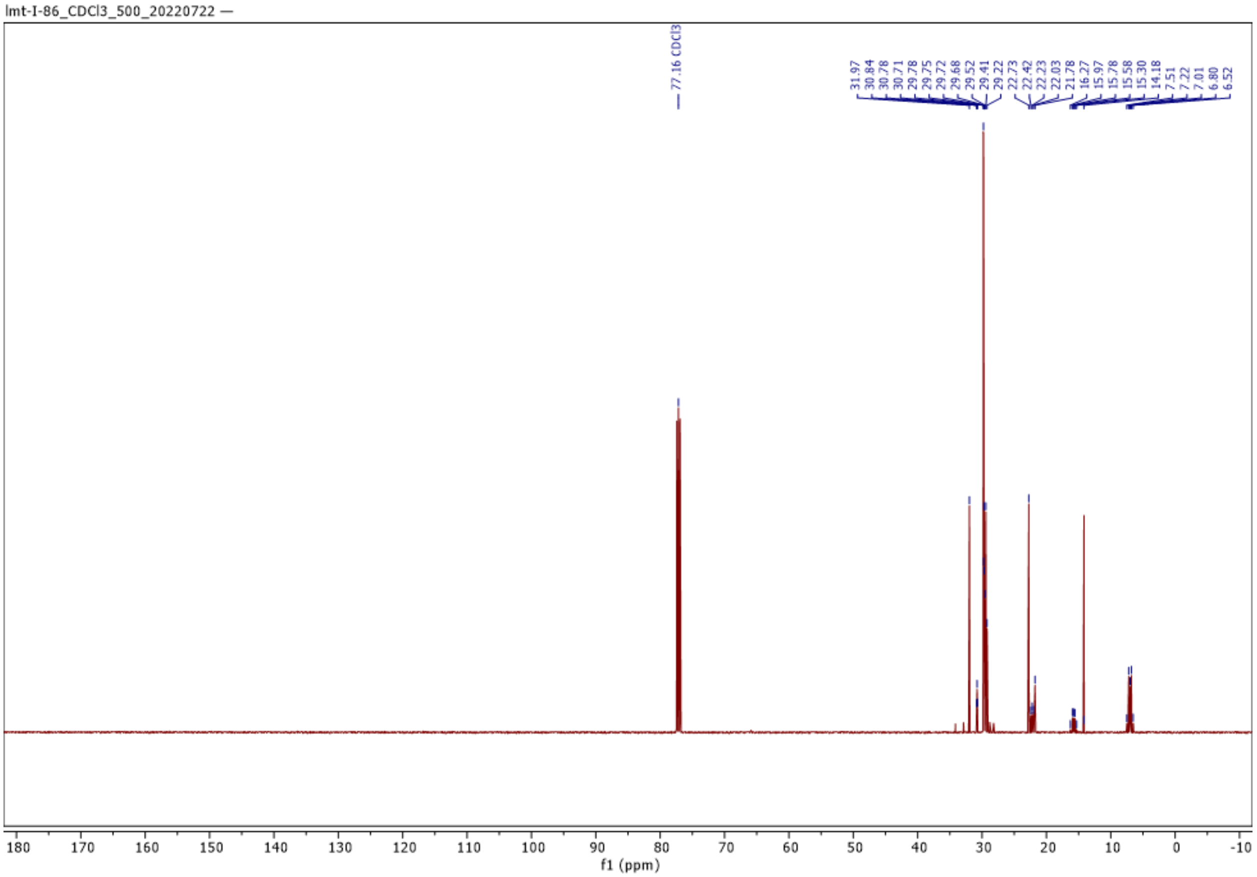

Figure S21. ^{13}C NMR of $^{\mathbf{Me}}$**P2P-16,16-Br** in $CDCl_3$

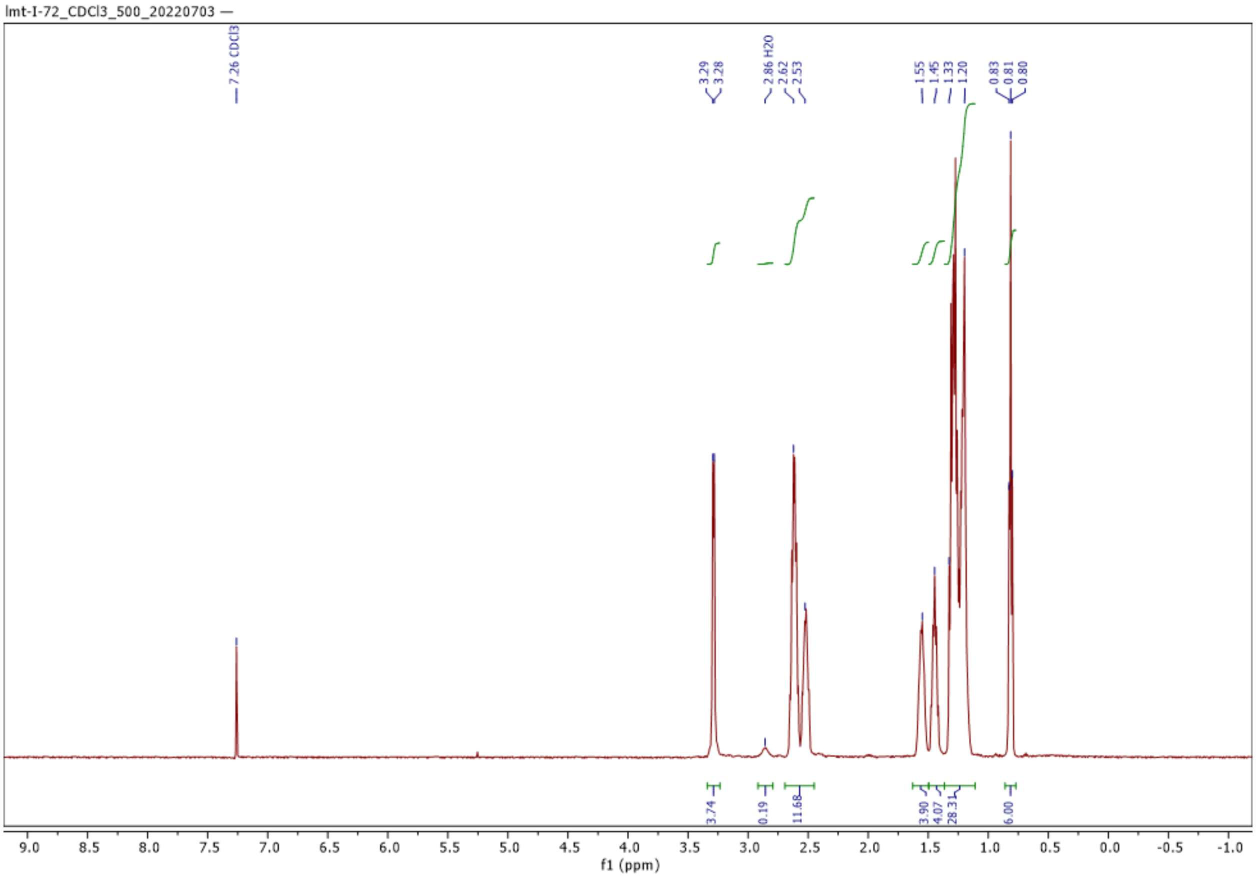

Figure S22. ^{1}H NMR of $^{\mathbf{Et}}$**P2P-8,8-Br** in $CDCl_3$

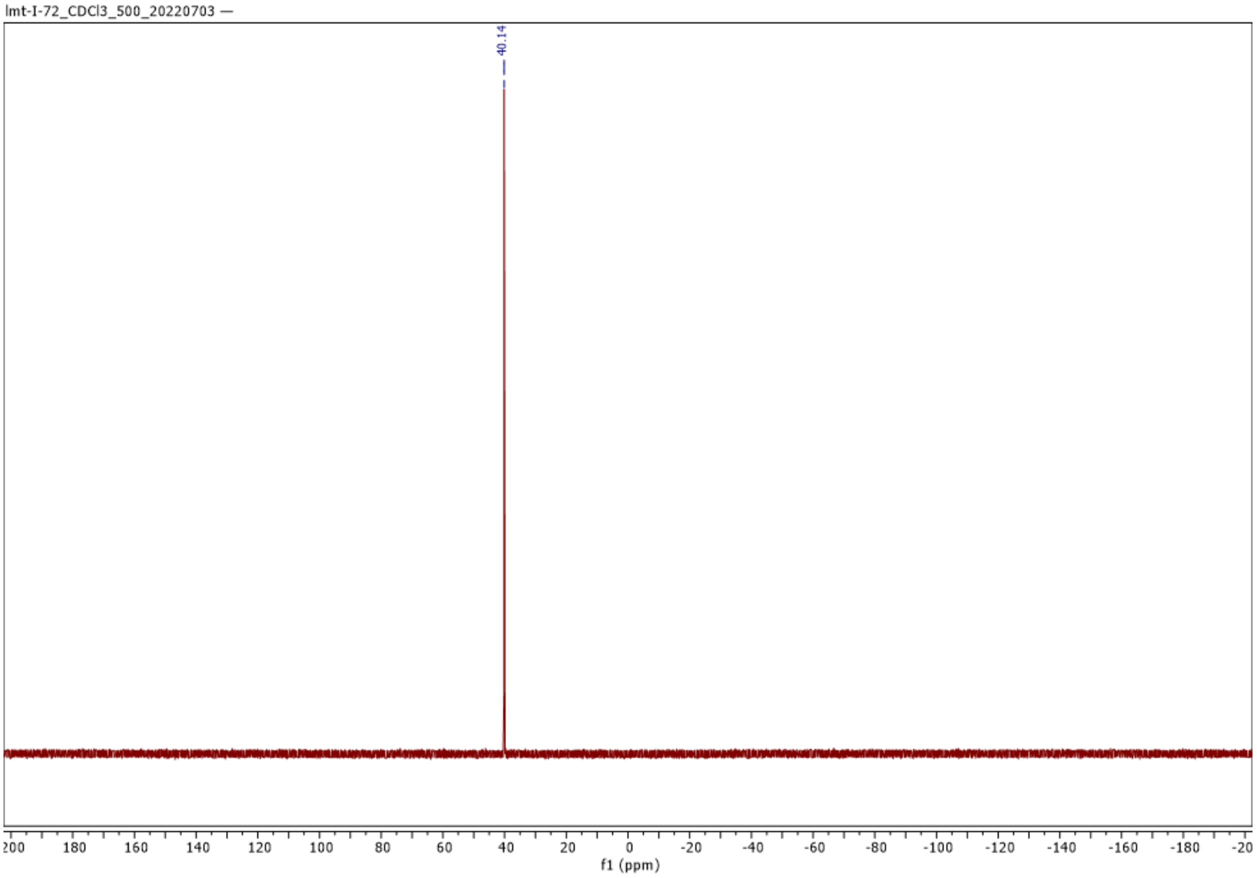

Figure S23. ^{31}P NMR of **^{Et}P2P-8,8-Br** in $CDCl_3$

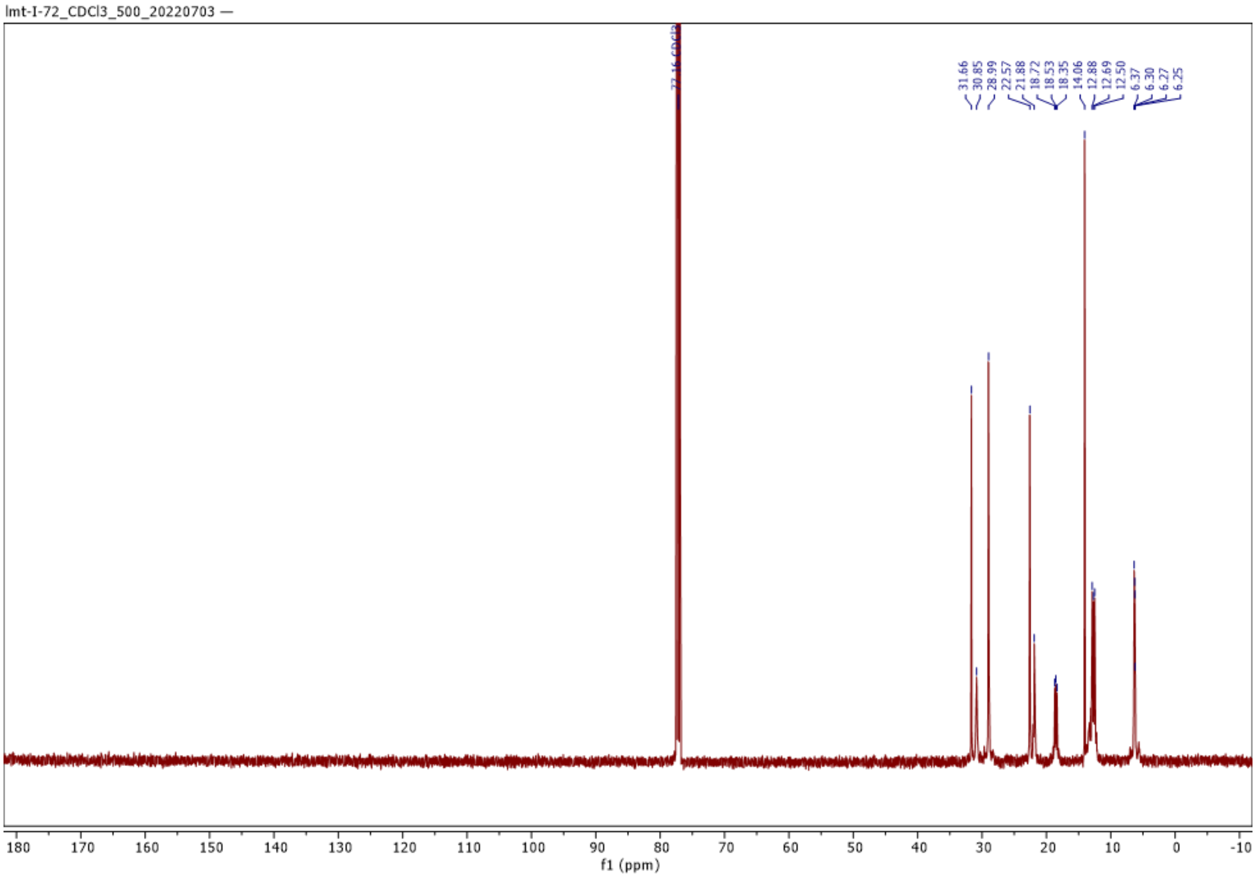

Figure S24. ^{13}C NMR of **^{Et}P2P-8,8-Br** in $CDCl_3$

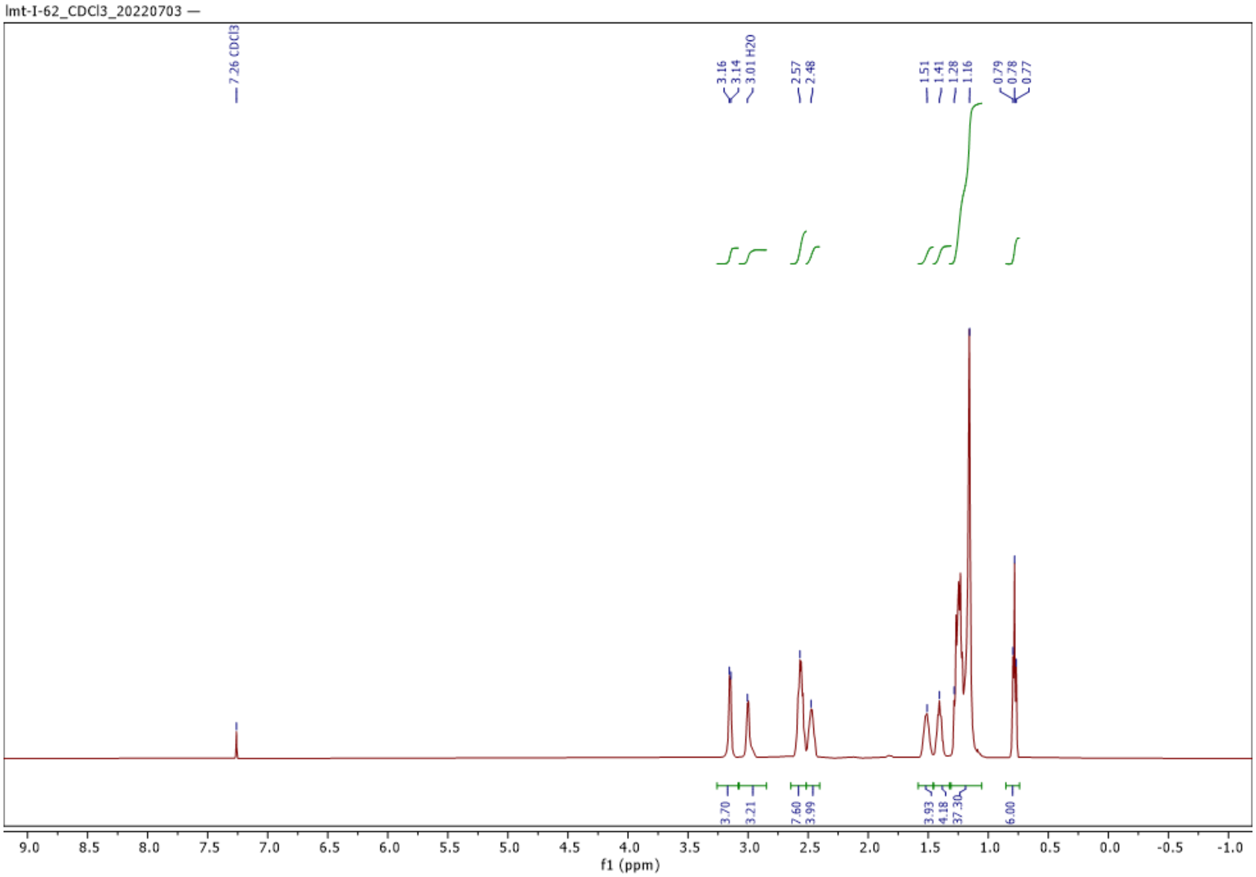

Figure S25. ^{1}H NMR of **^{Et}P2P-10,10-Br** in $CDCl_3$

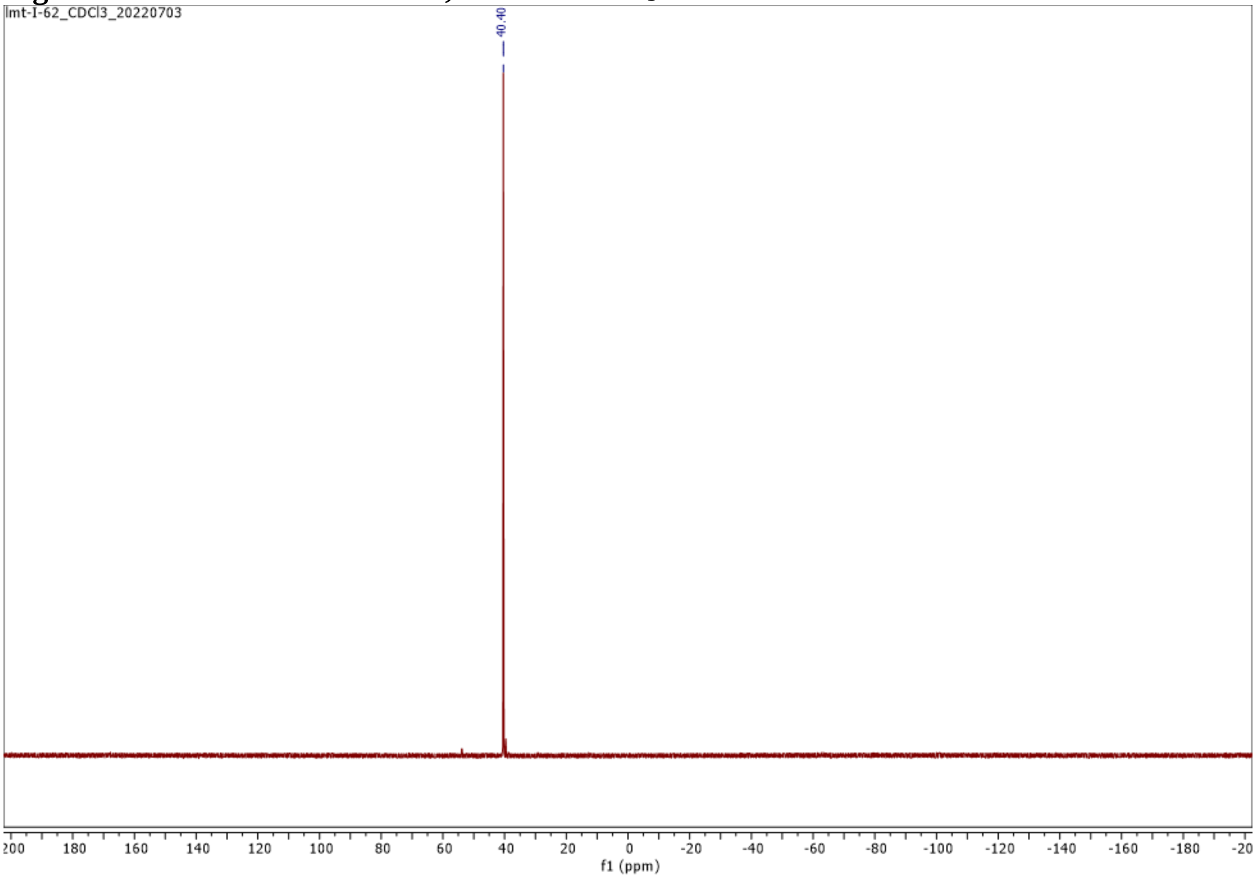

Figure S26. ^{31}P NMR of **^{Et}P2P-10,10-Br** in $CDCl_3$

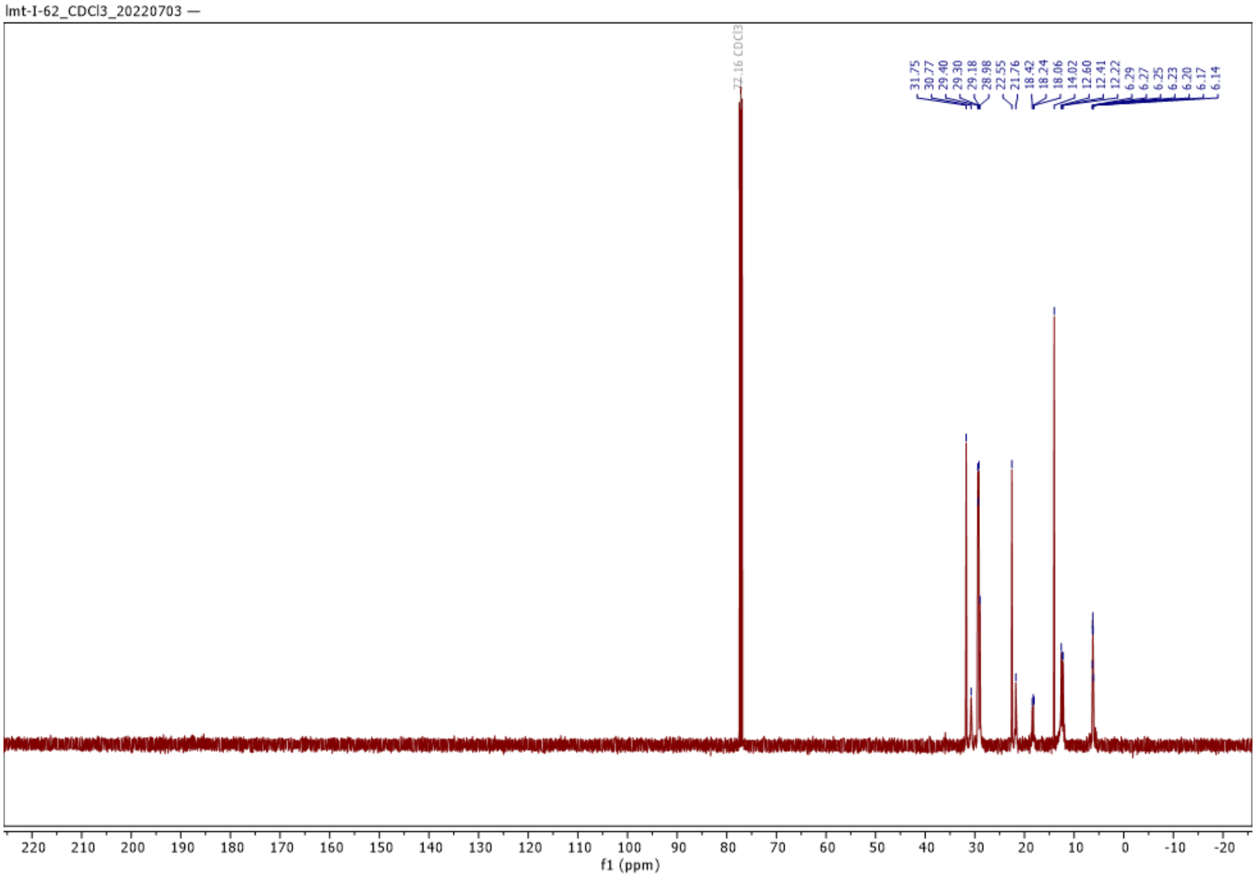

Figure S27. ^{13}C NMR of ^{Et}**P2P-10,10-Br** in $CDCl_3$

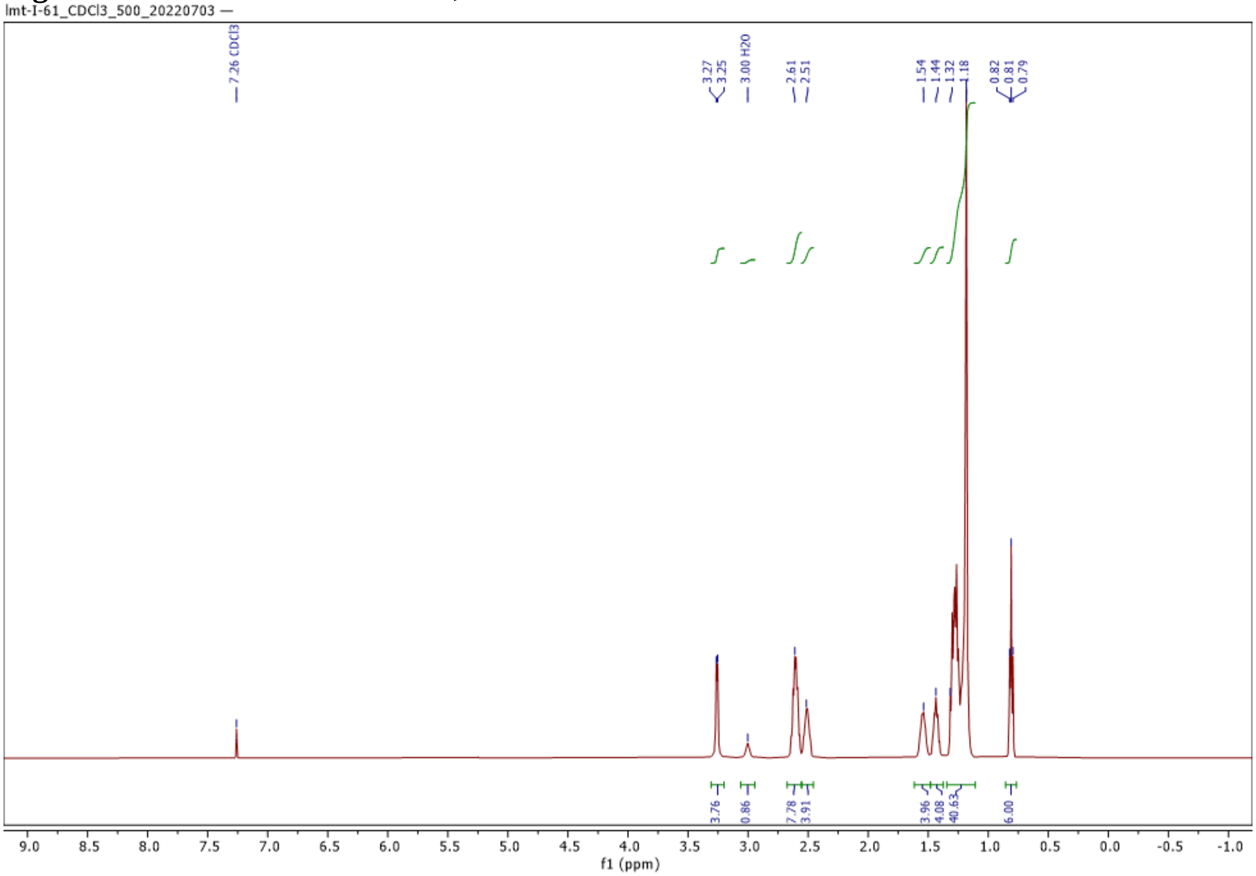

Figure S28. ^{1}H NMR of ^{Et}**P2P-11,11-Br** in $CDCl_3$

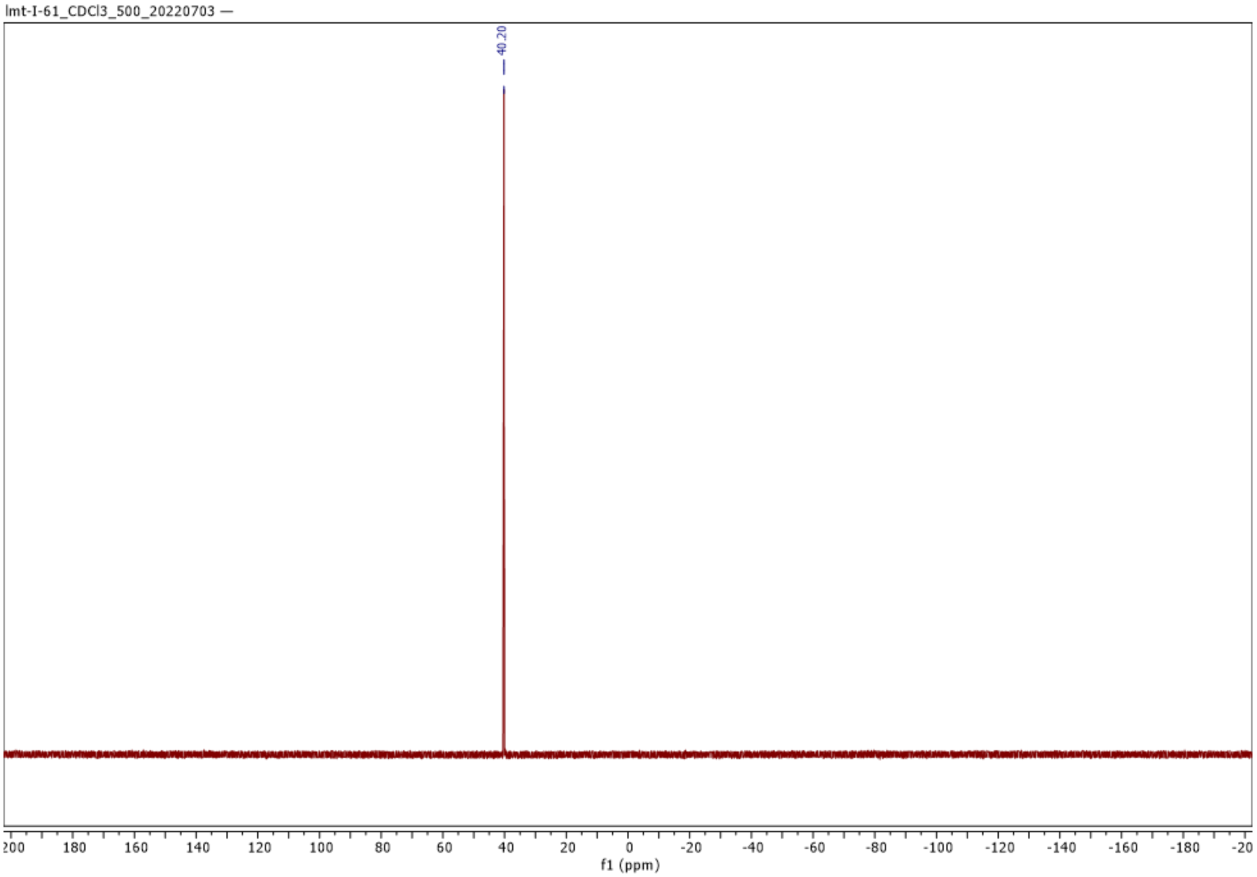

Figure S29. ^{31}P NMR of $^{\mathbf{Et}}$**P2P-11,11-Br** in $CDCl_3$

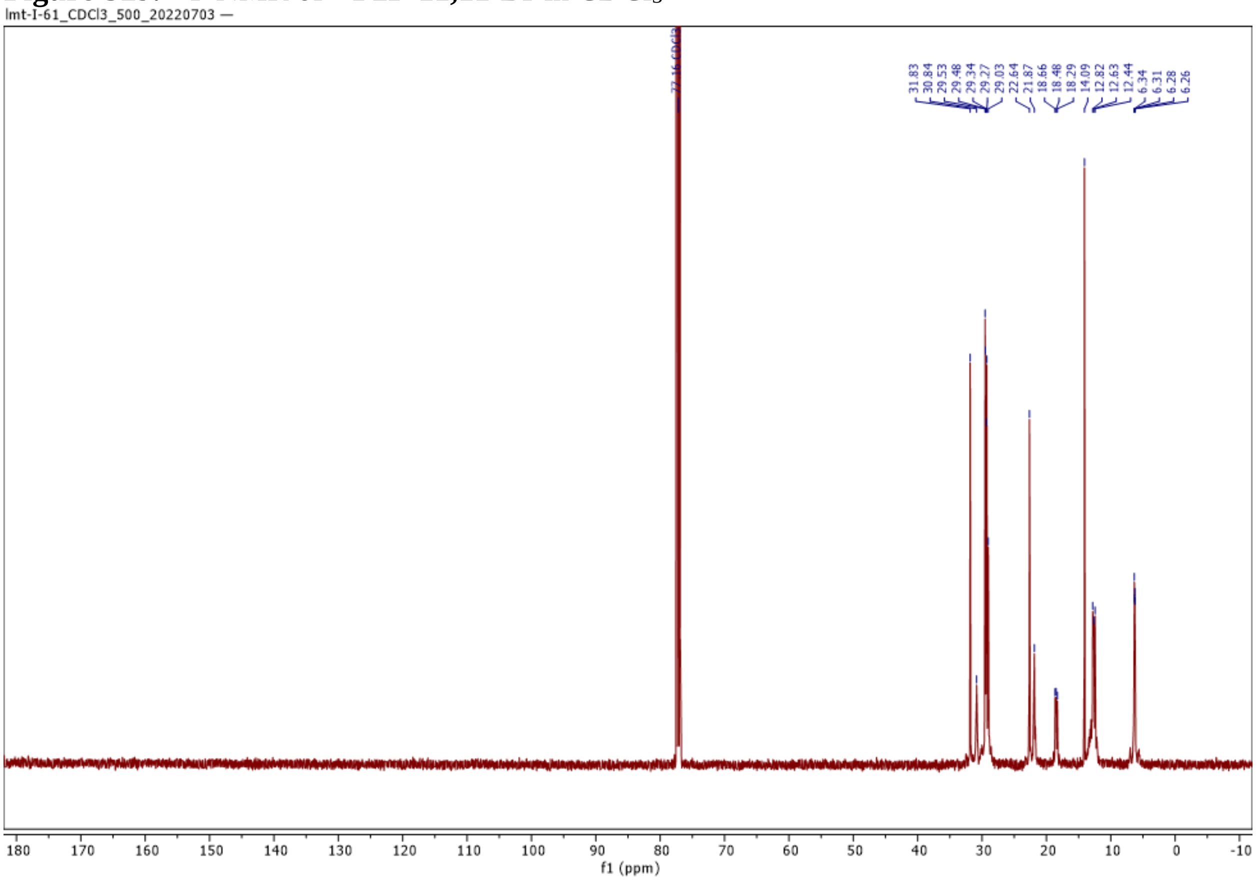

Figure S30. ^{13}C NMR of $^{\mathbf{Et}}$**P2P-11,11-Br** in $CDCl_3$

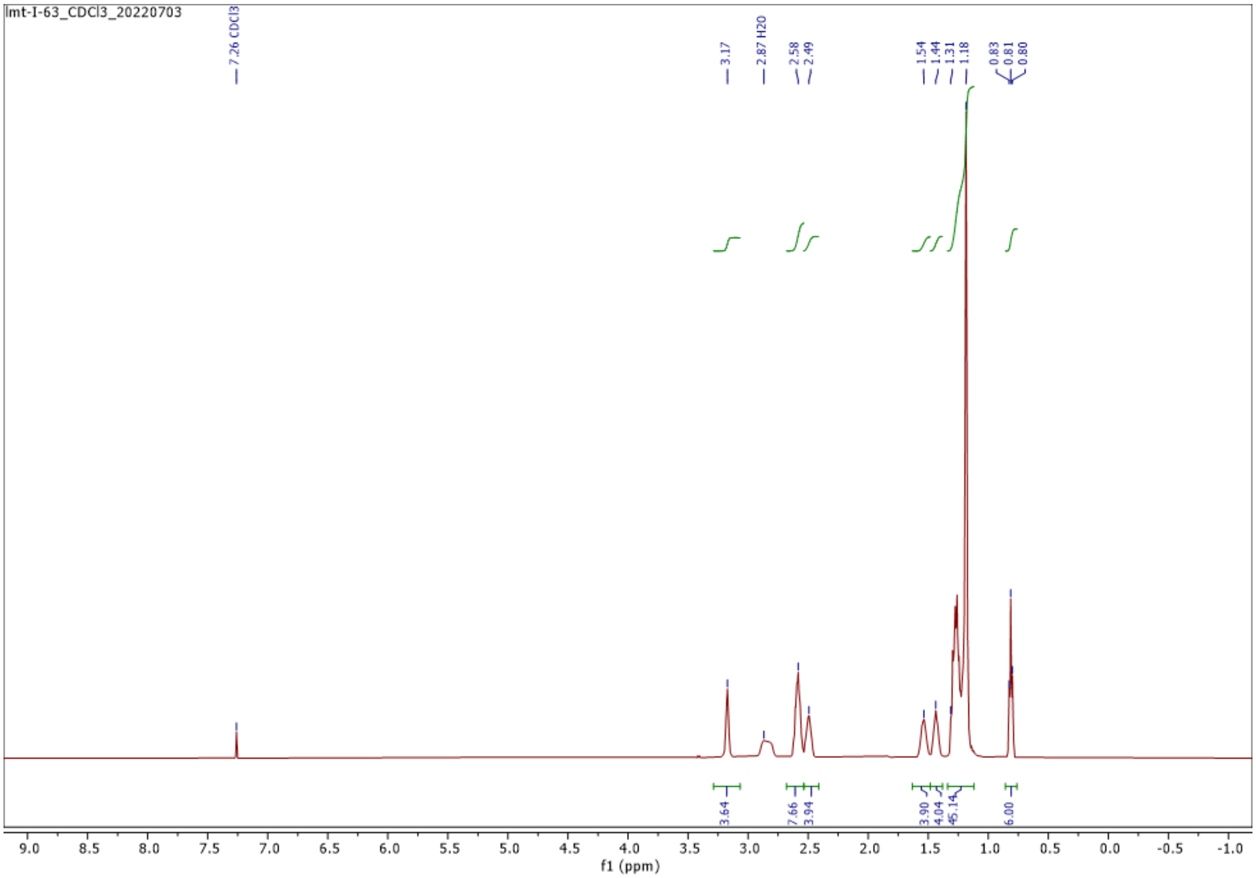

Figure S31. ^{1}H NMR of ^{Et}**P2P-12,12-Br** in $CDCl_3$

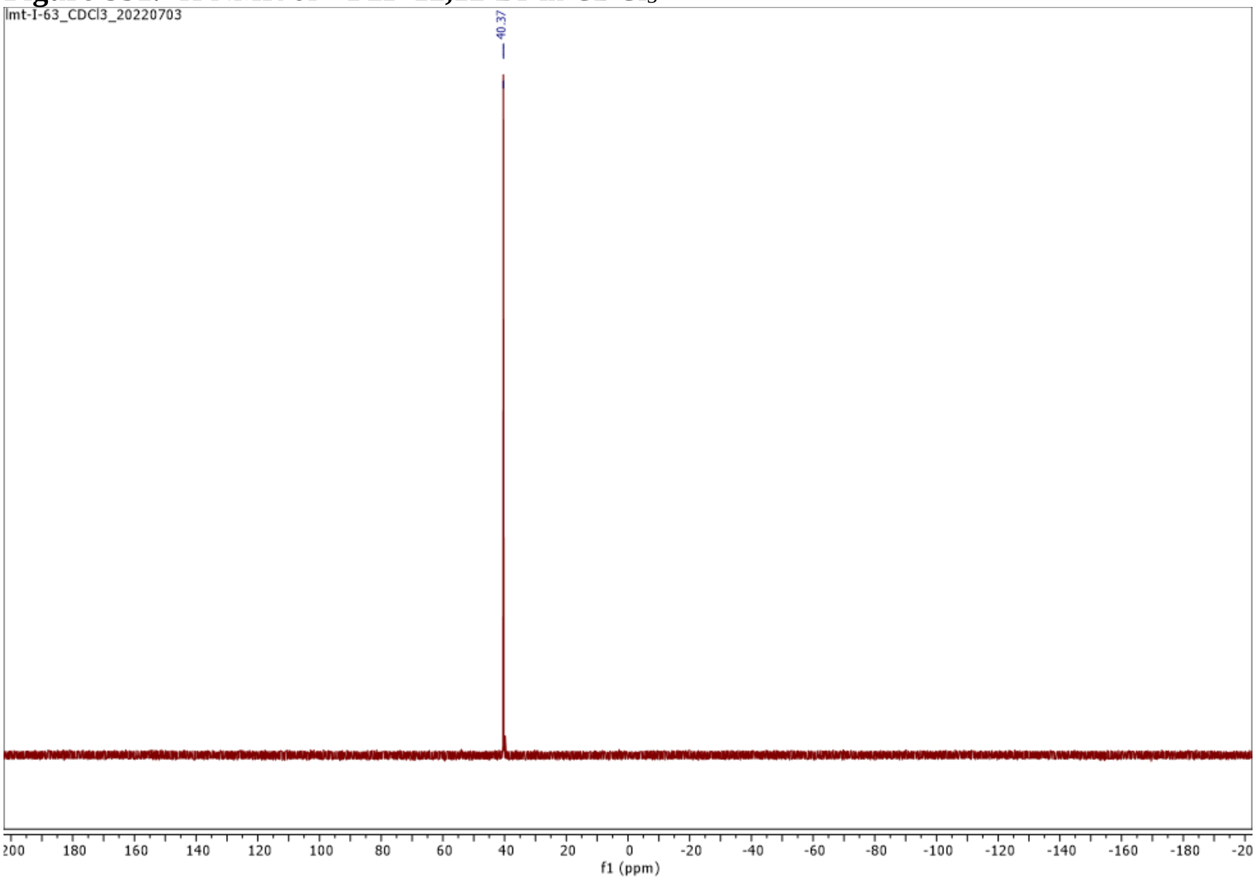

Figure S32. ^{31}P NMR of ^{Et}**P2P-12,12-Br** in $CDCl_3$

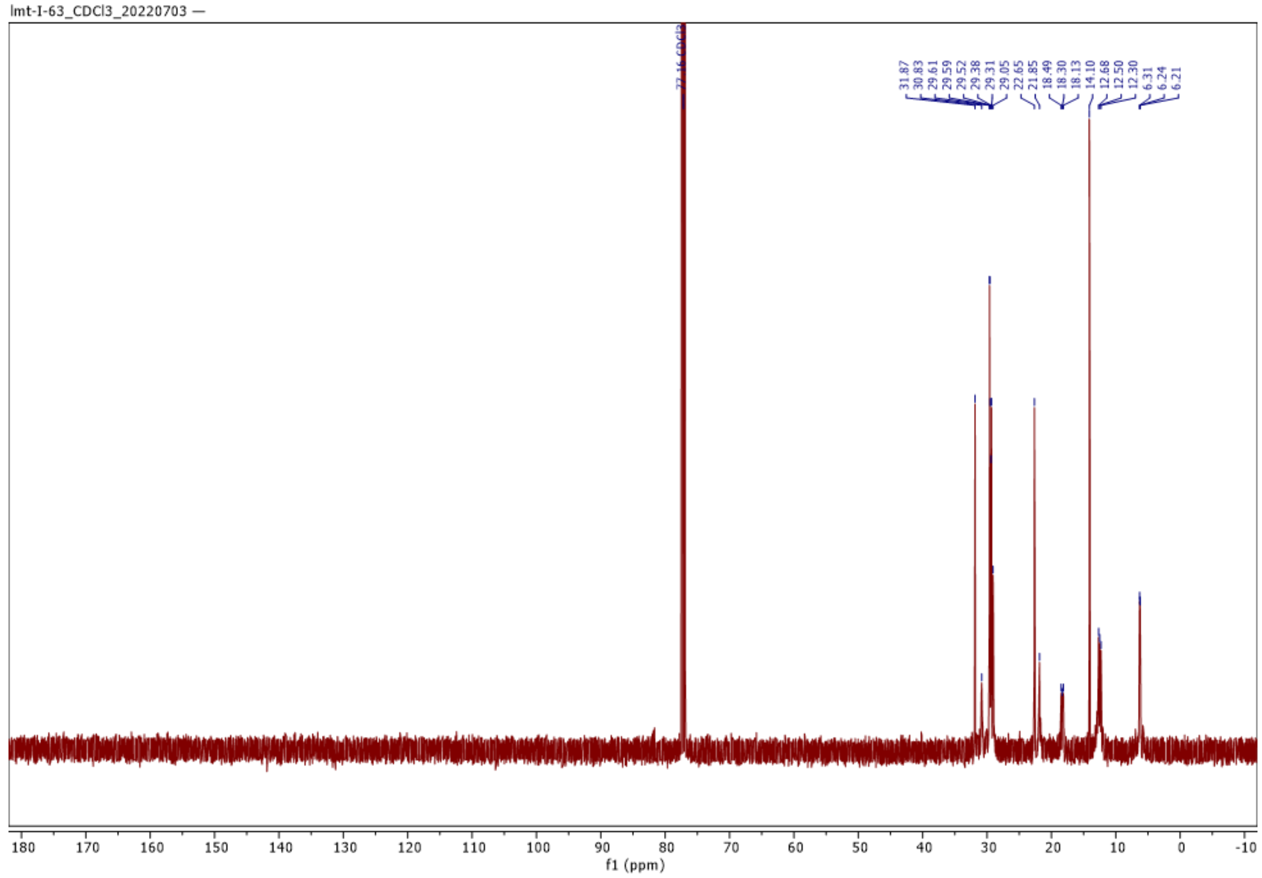

Figure S33. ^{13}C NMR of **^{Et}P2P-12,12-Br** in $CDCl_3$

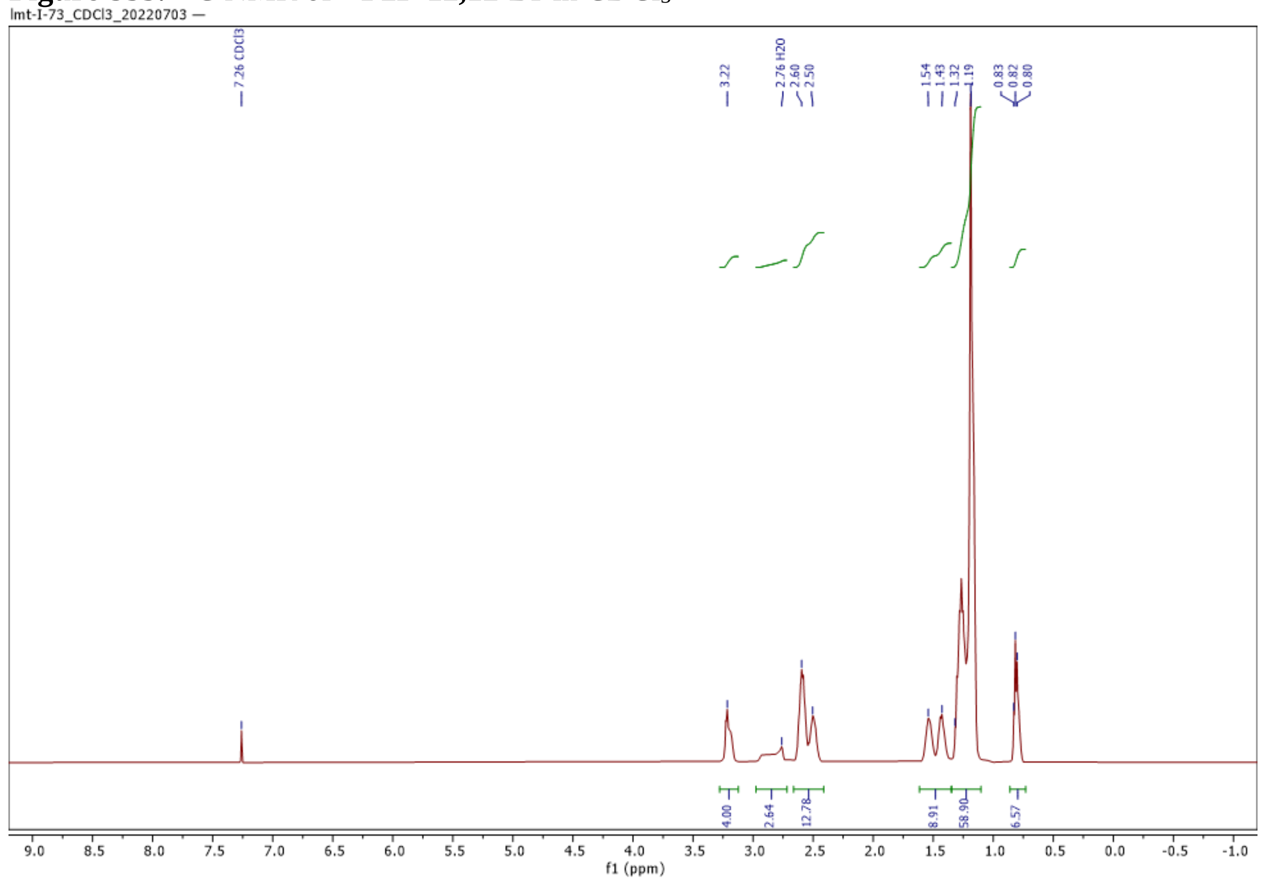

Figure S34. ^{1}H NMR of **^{Et}P2P-14,14-Br** in $CDCl_3$

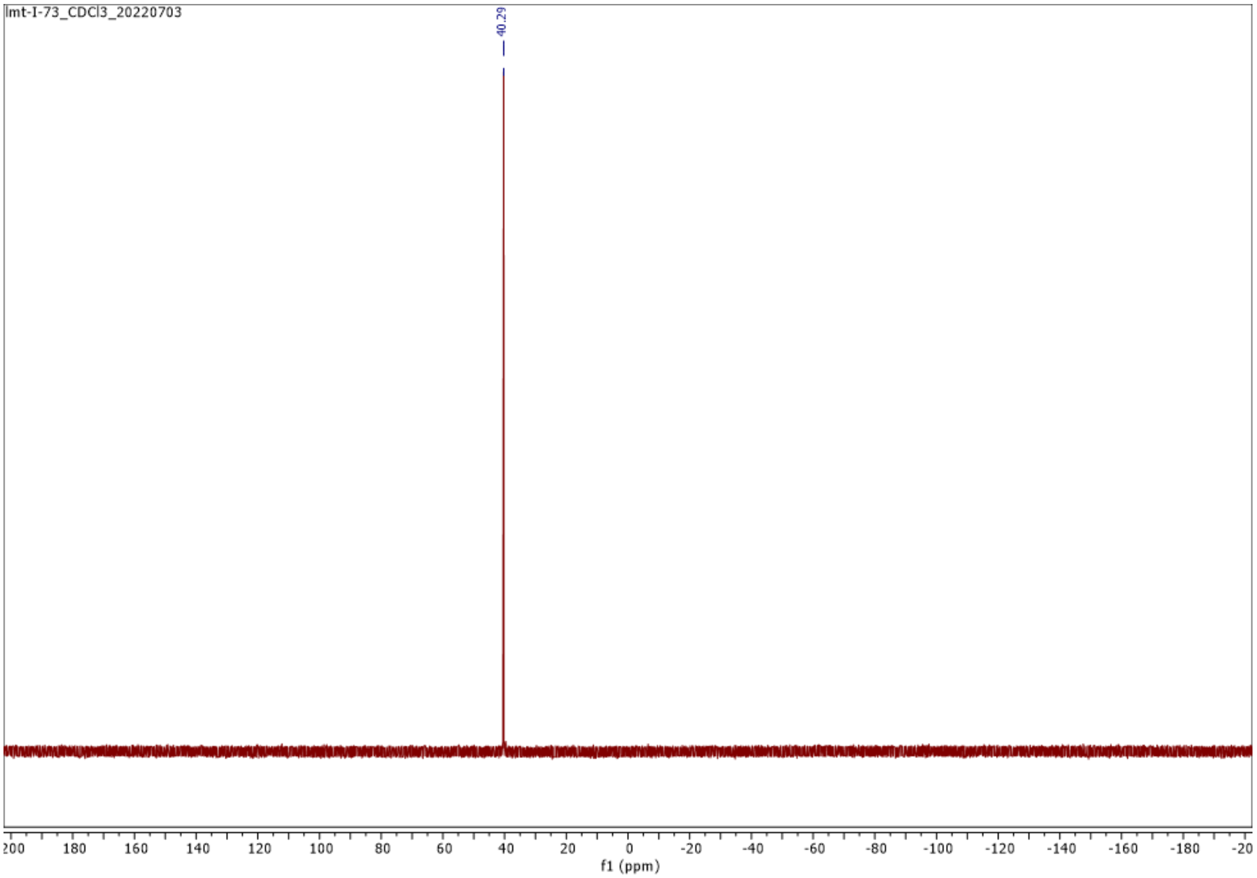

Figure S35. ^{31}P NMR of ^{Et}**P2P-14,14-Br** in $CDCl_3$

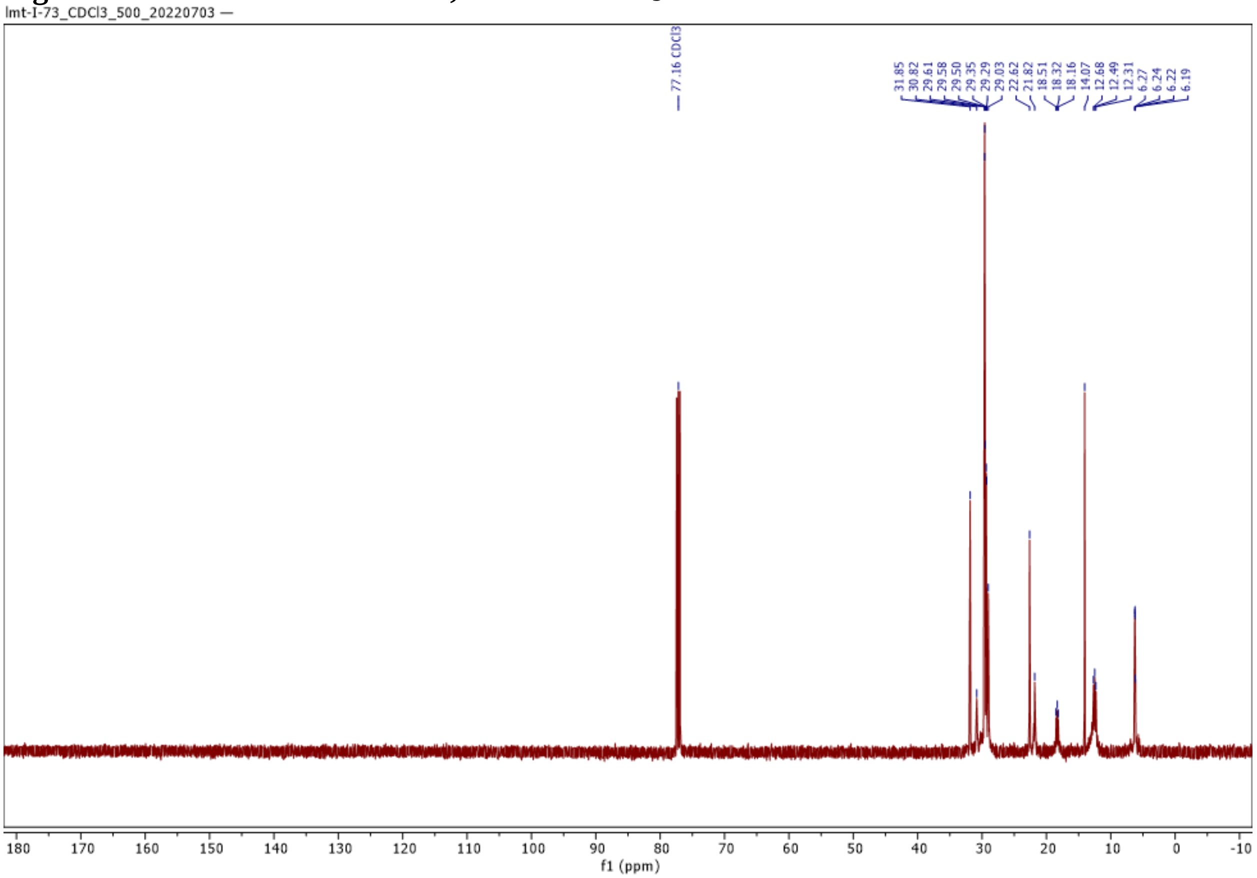

Figure S36. ^{13}C NMR of ^{Et}**P2P-14,14-Br** in $CDCl_3$

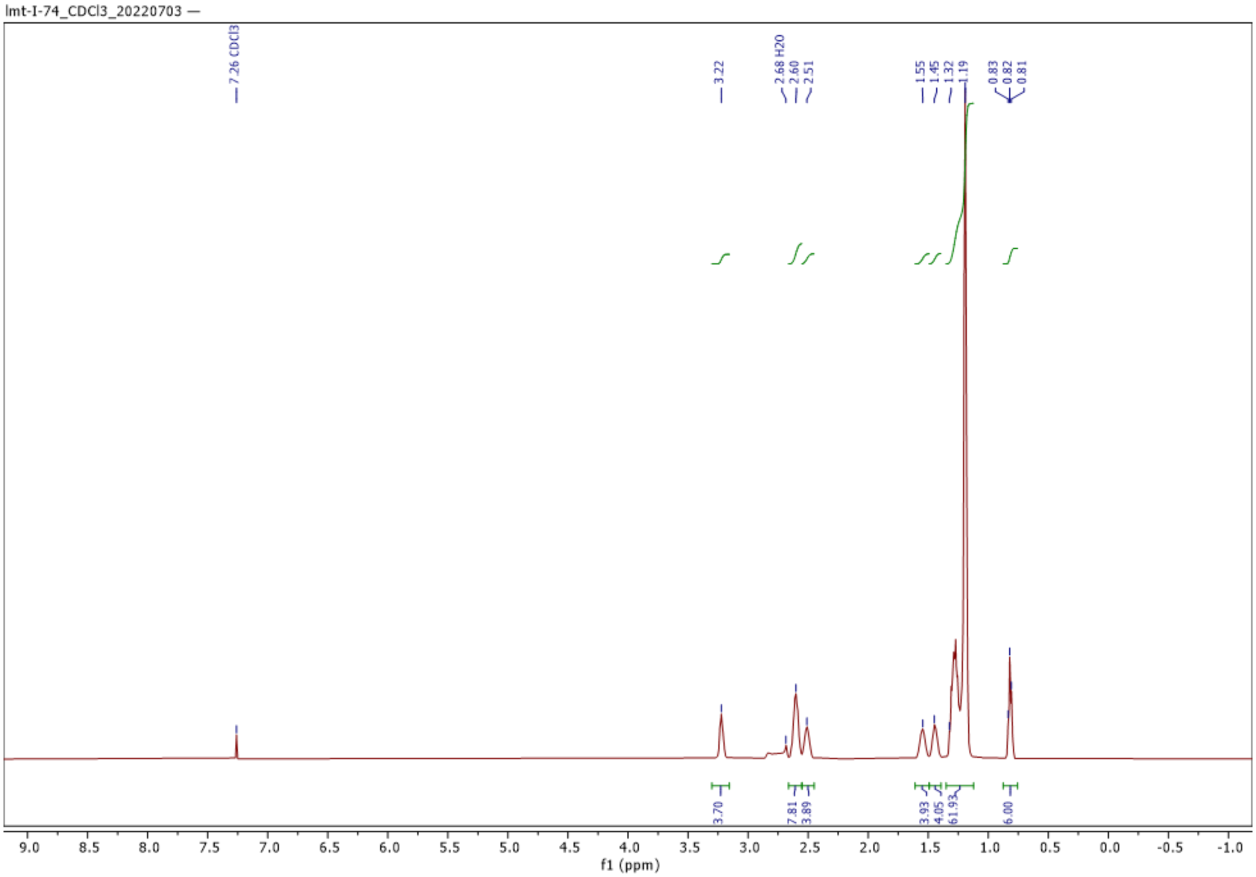

Figure S37. ^{1}H NMR of ^{Et}**P2P-16,16-Br** in $CDCl_3$

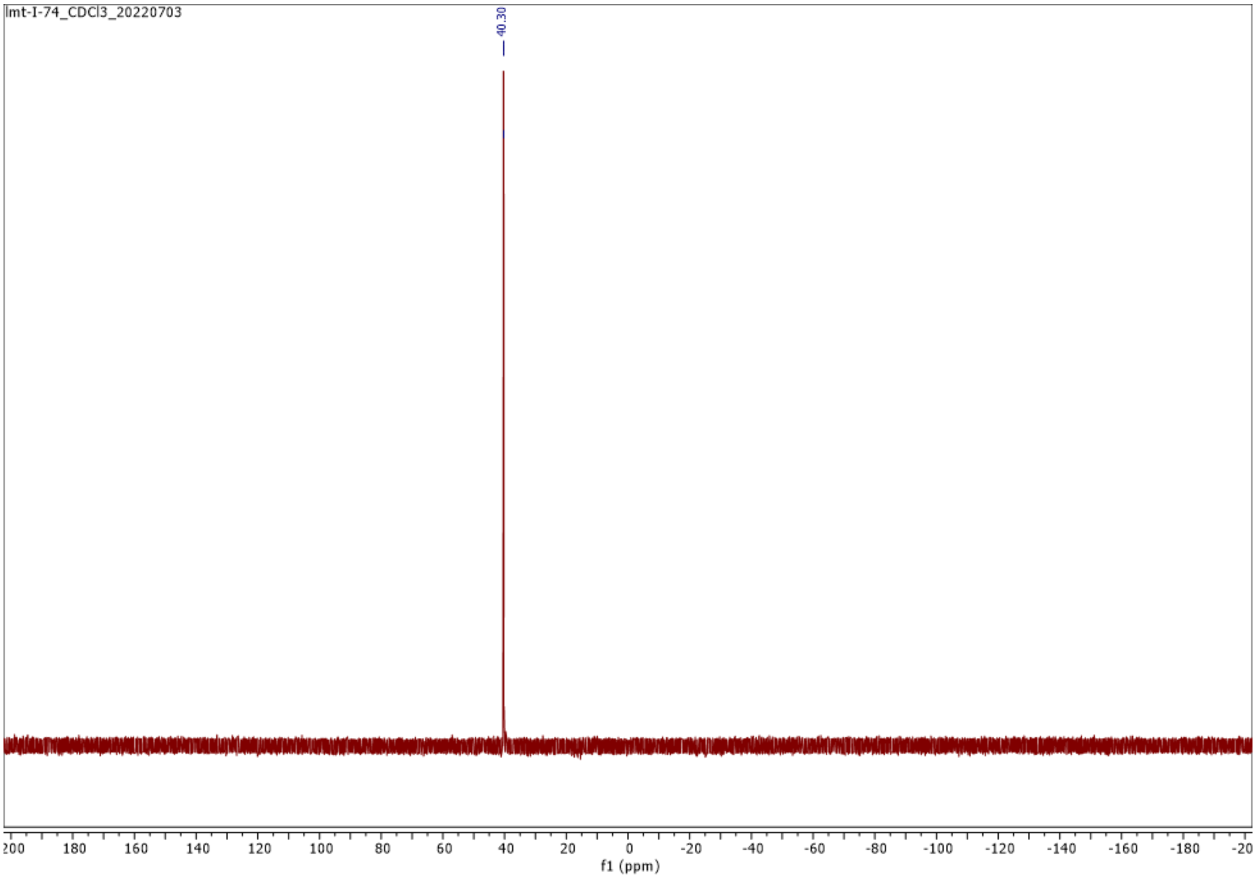

Figure S38. ^{31}P NMR of ^{Et}**P2P-16,16-Br** in $CDCl_3$

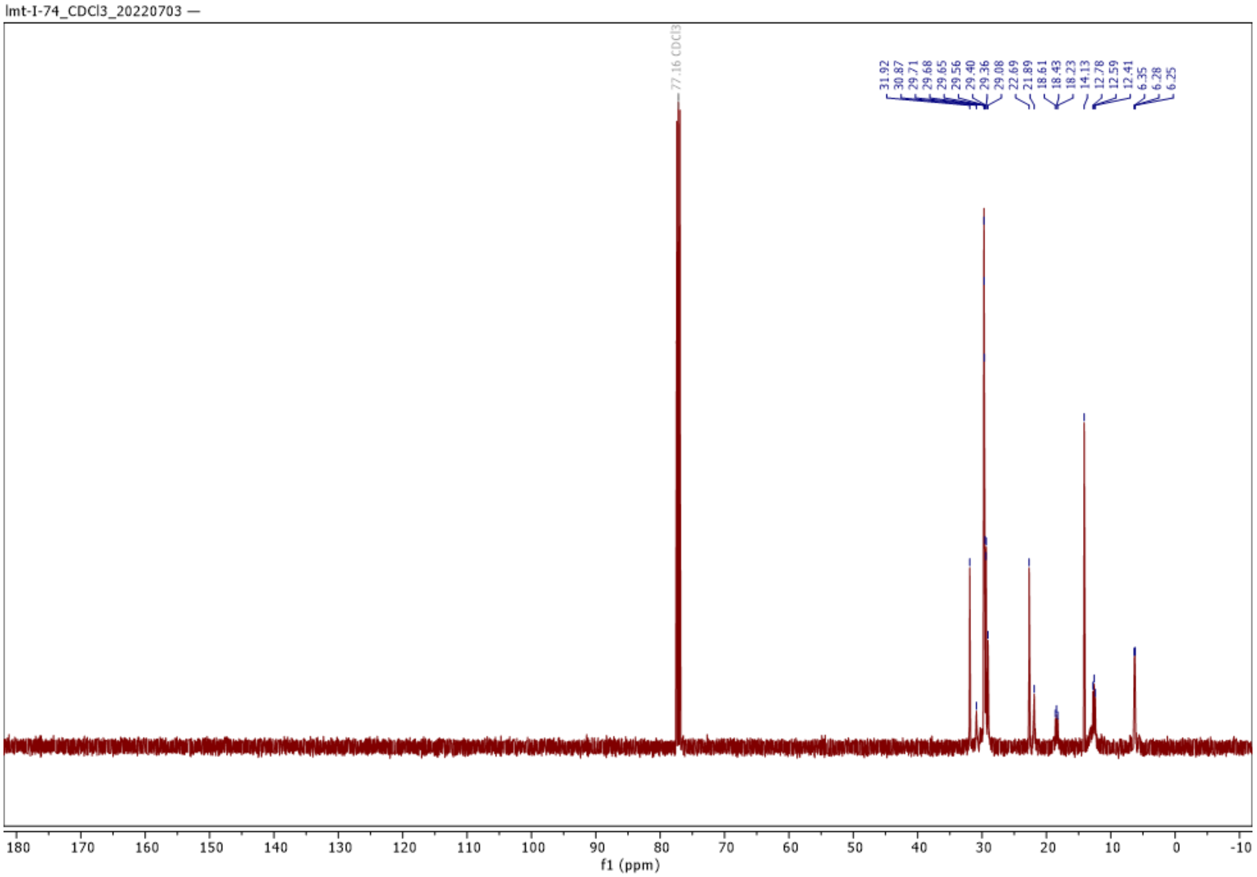

Figure S39. ^{13}C NMR of $^{\mathbf{Et}}$**P2P-16,16-Br** in $CDCl_3$

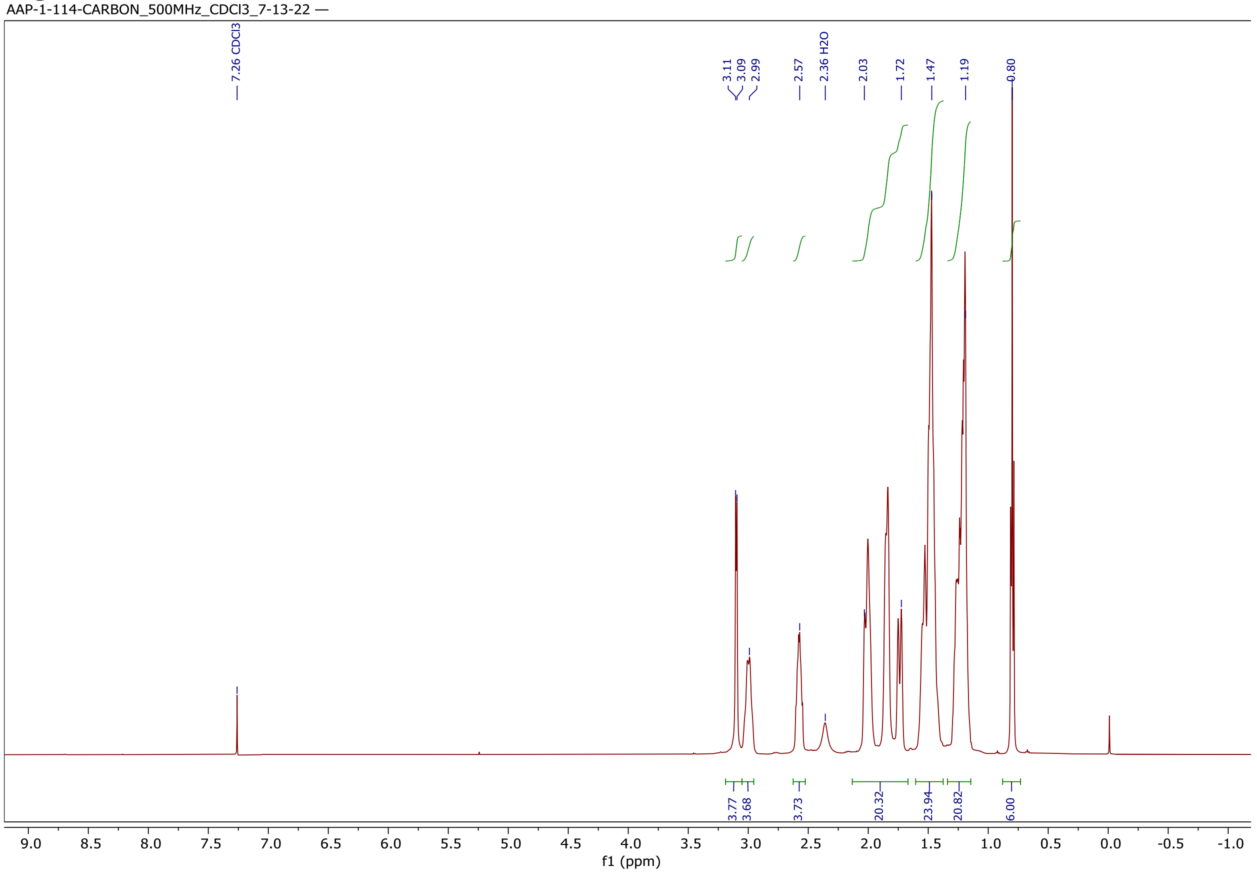

Figure S40. ^{1}H NMR of $^{\mathbf{Cy}}$**P2P-8,8-Br** in $CDCl_3$

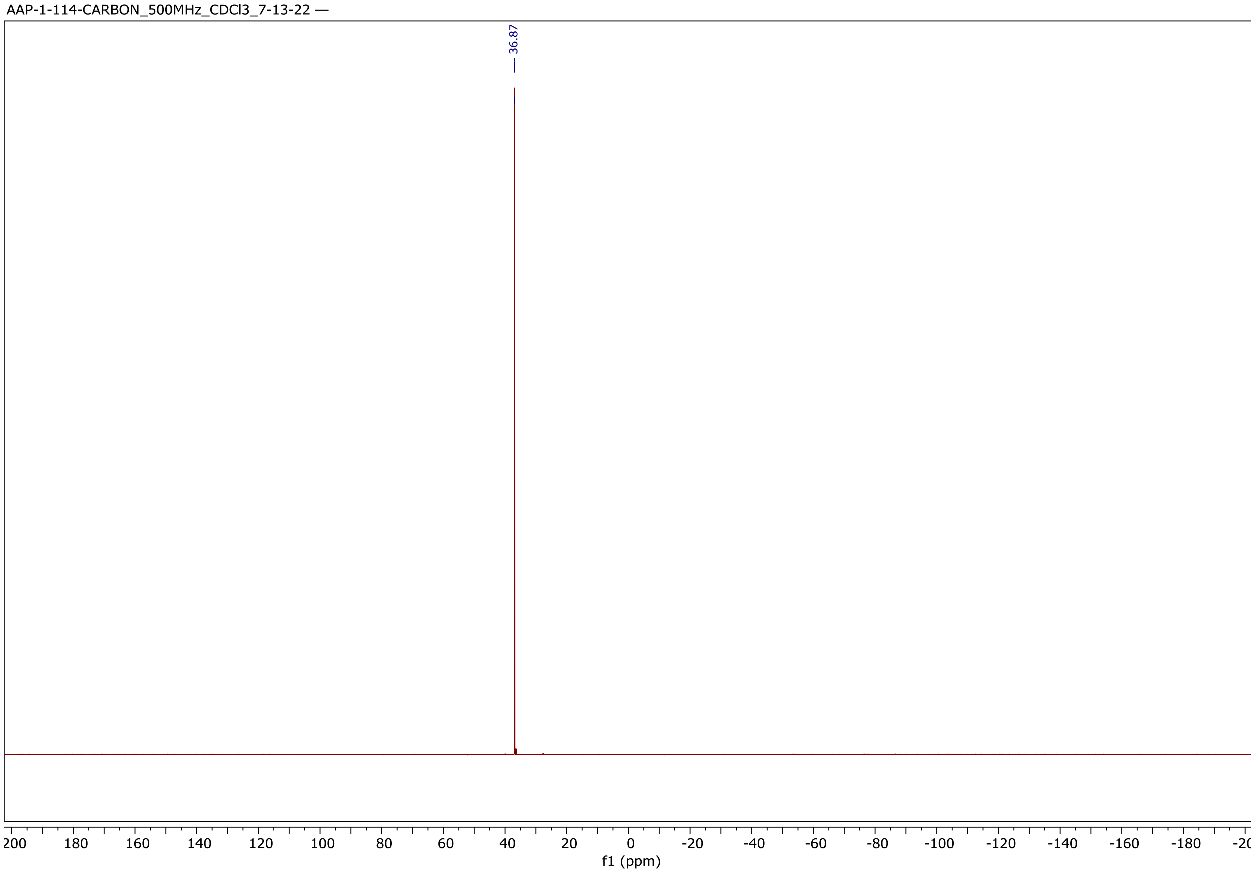

Figure S41. ^{31}P NMR of **^{Cy}P2P-8,8-Br** in $CDCl_3$

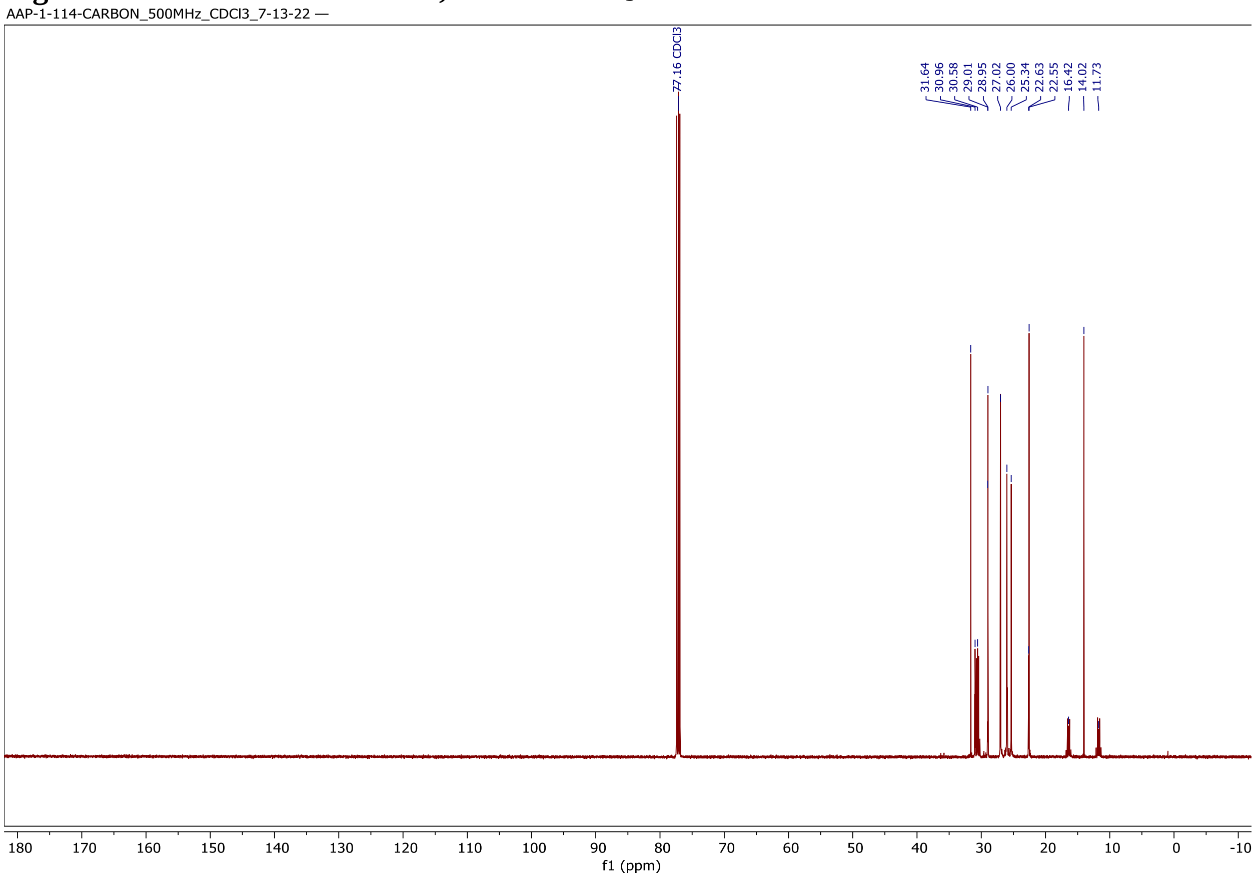

Figure S42. ^{13}C NMR of **^{Cy}P2P-8,8-Br** in $CDCl_3$

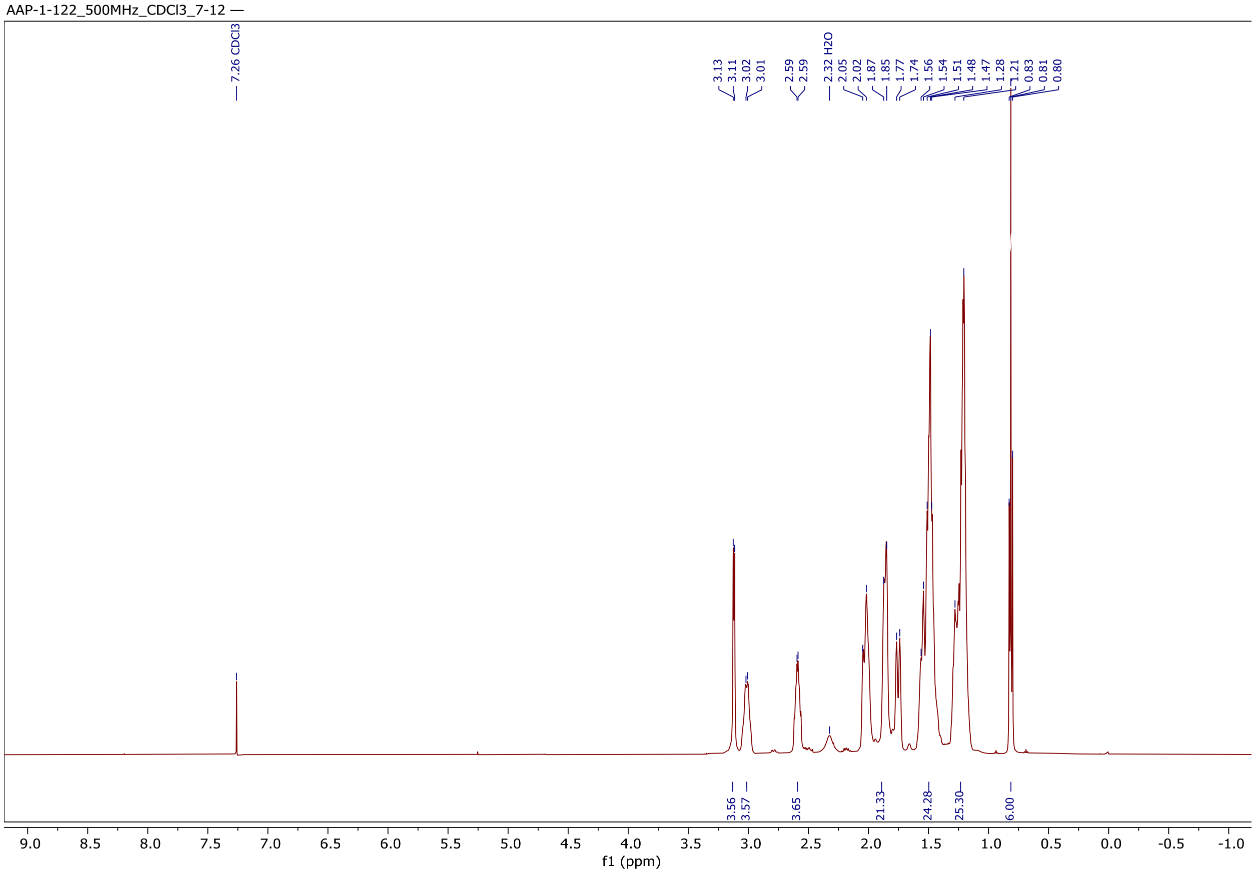

Figure S43. ^{1}H NMR of **^{Cy}P2P-9,9-Br** in $CDCl_3$

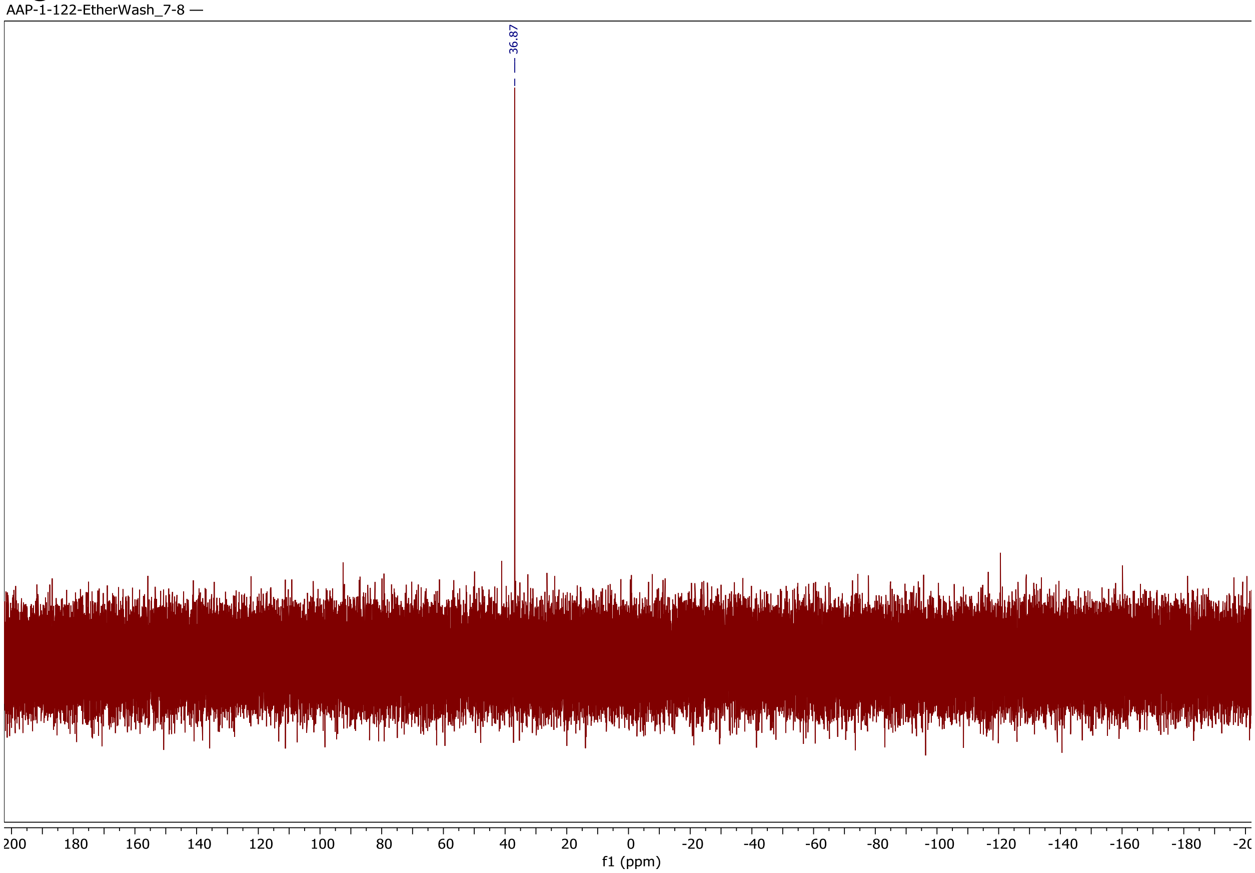

Figure S44. ^{31}P NMR of **^{Cy}P2P-9,9-Br** in $CDCl_3$

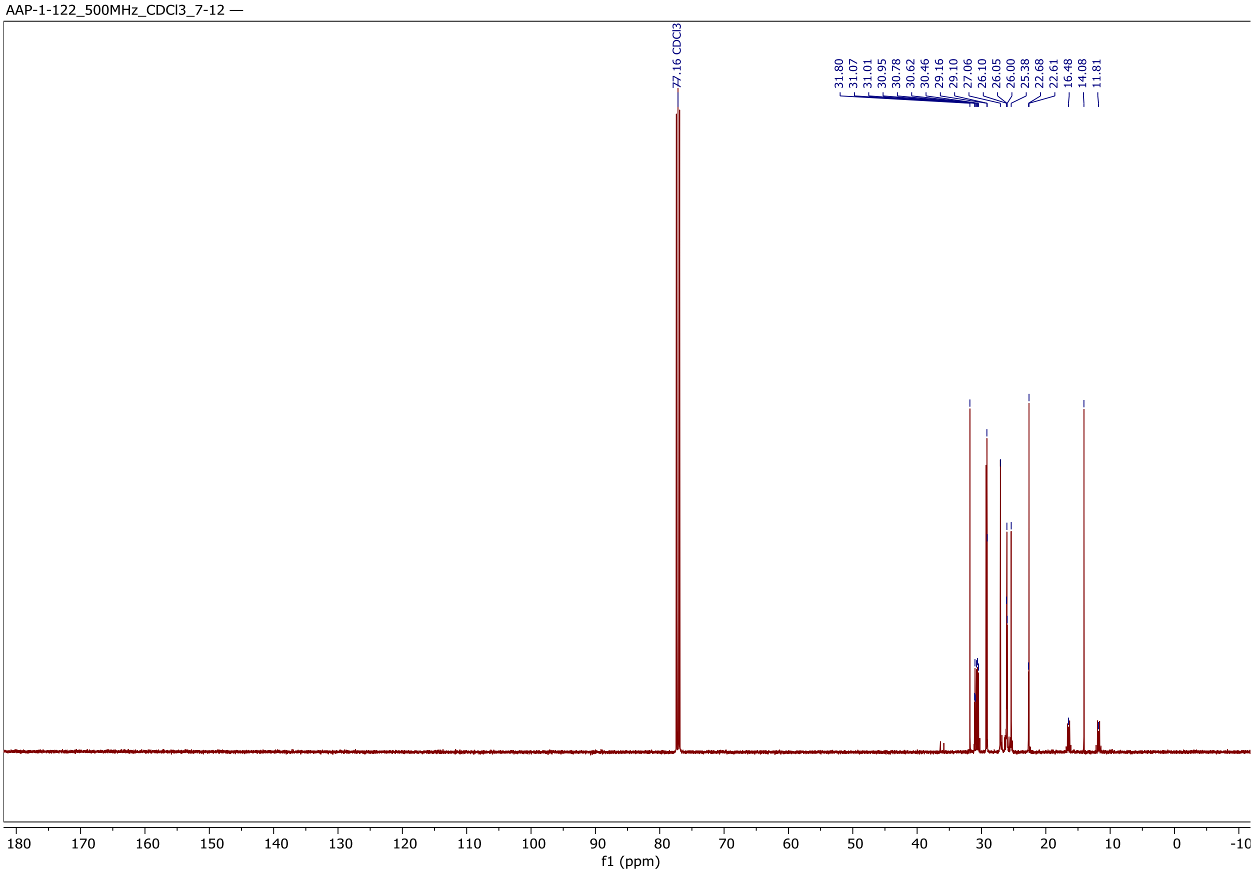

Figure S45. ^{13}C NMR of ^{Cy}**P2P-9,9-Br** in $CDCl_3$

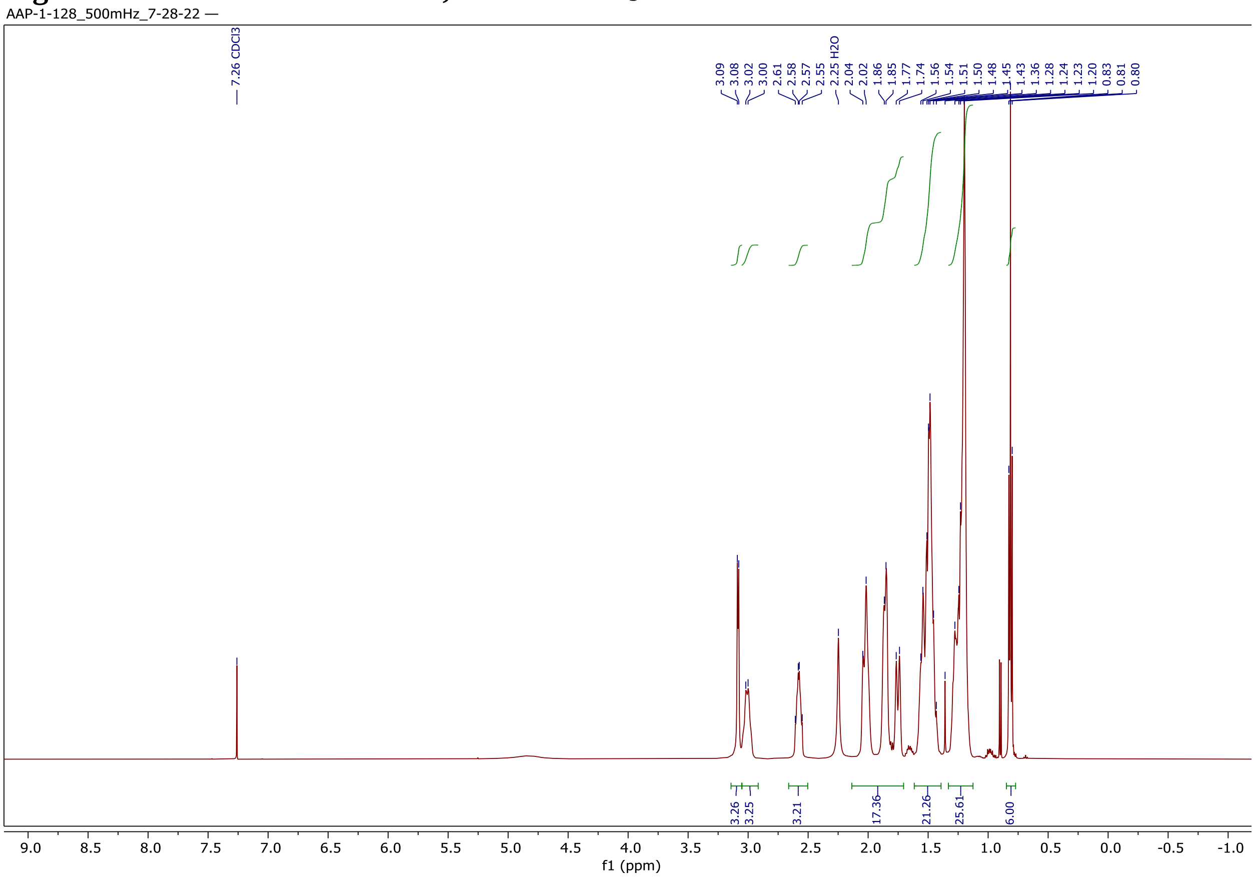

Figure S46. ^{1}H NMR of ^{Cy}**P2P-10,10-Br** in $CDCl_3$

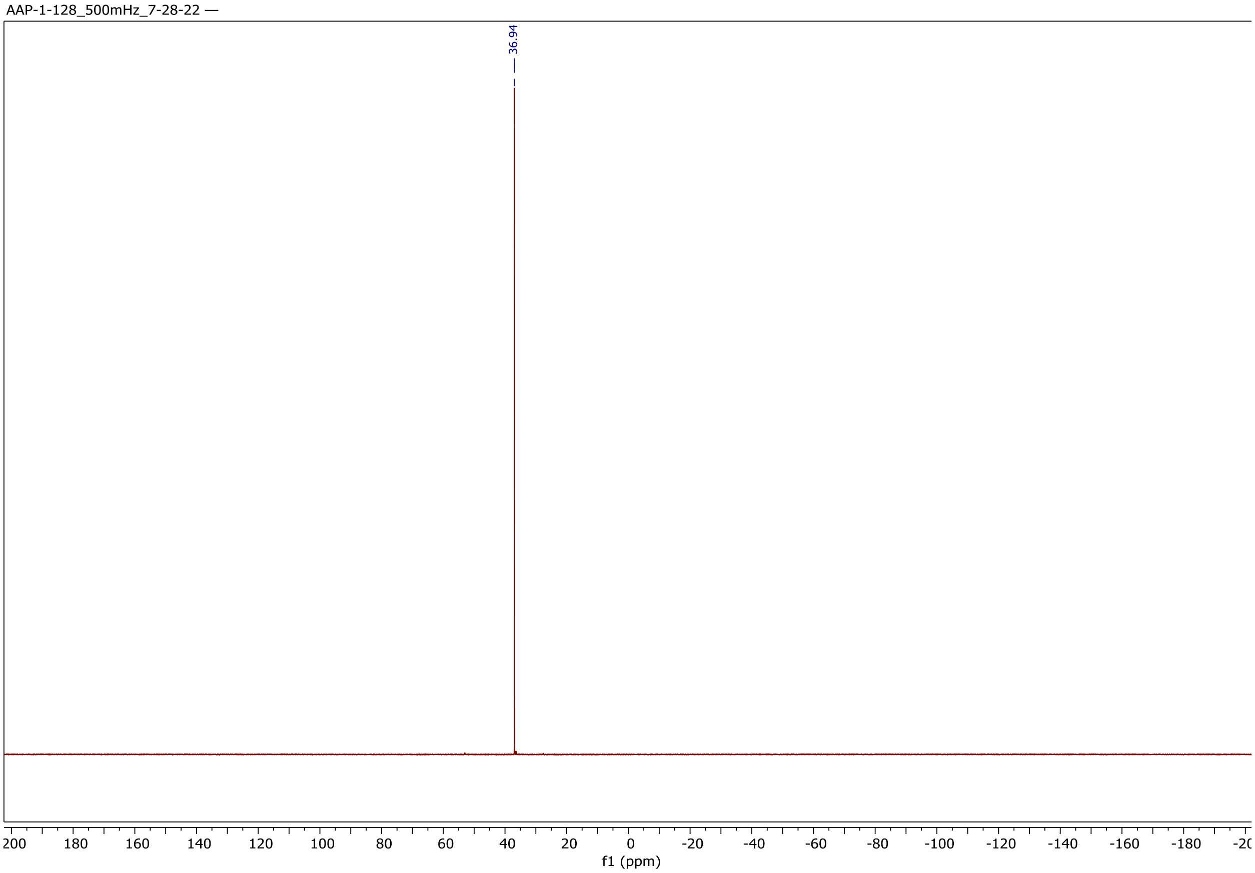

Figure S47. ^{31}P NMR of ^{Cy}**P2P-10,10-Br** in $CDCl_3$

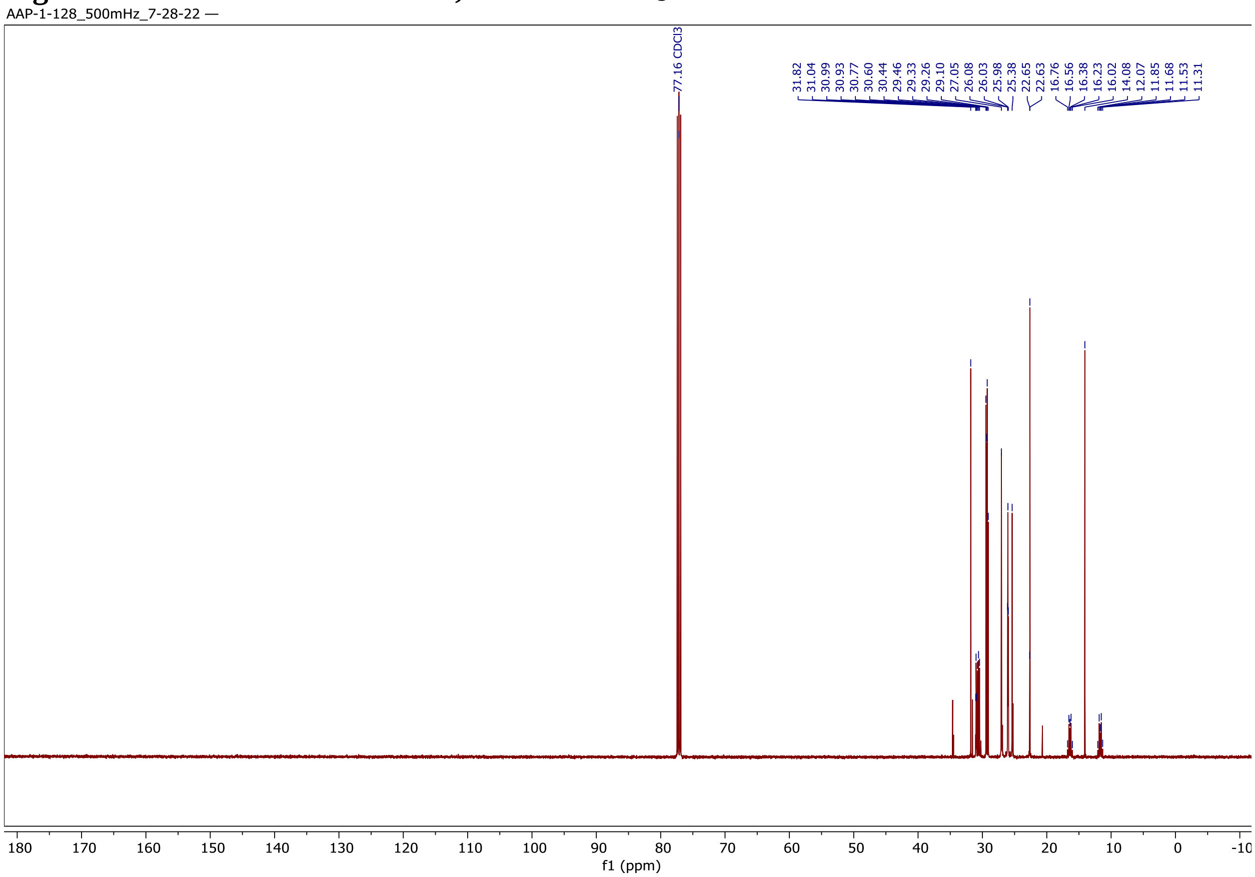

Figure S48. ^{13}C NMR of ^{Cy}**P2P-10,10-Br** in $CDCl_3$

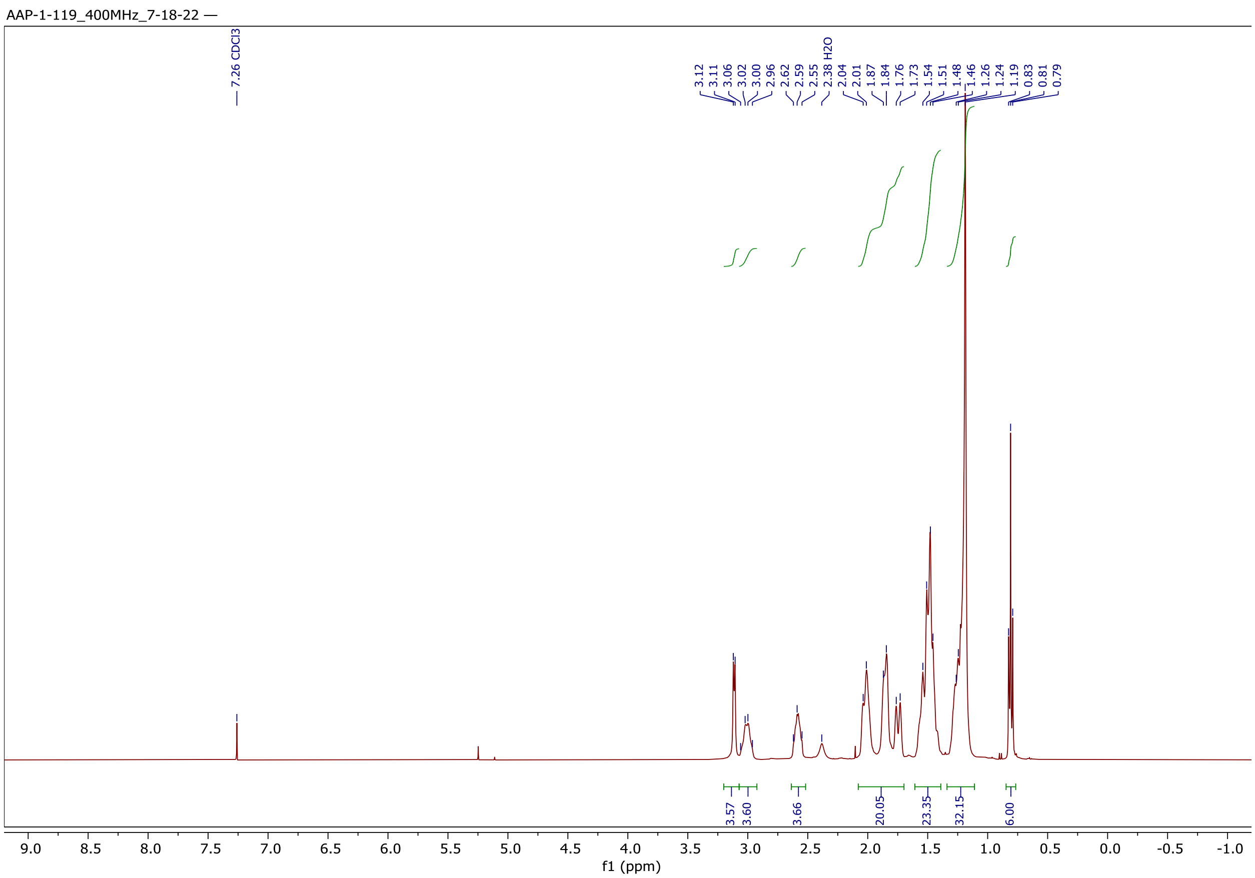

Figure S49. ^{1}H NMR of ^{Cy}**P2P-11,11-Br** in $CDCl_3$

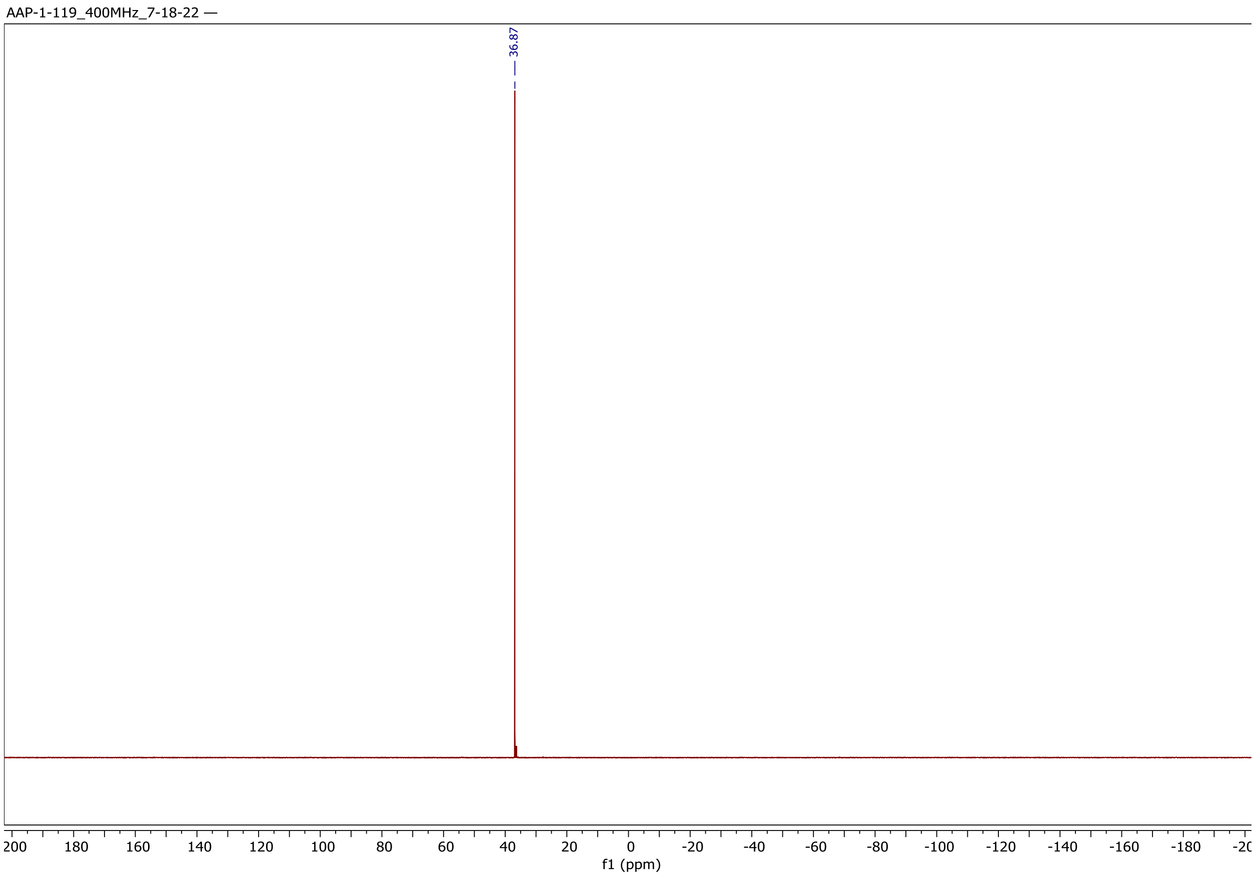

Figure S50. ^{1}P NMR of ^{Cy}**P2P-11,11-Br** in $CDCl_3$

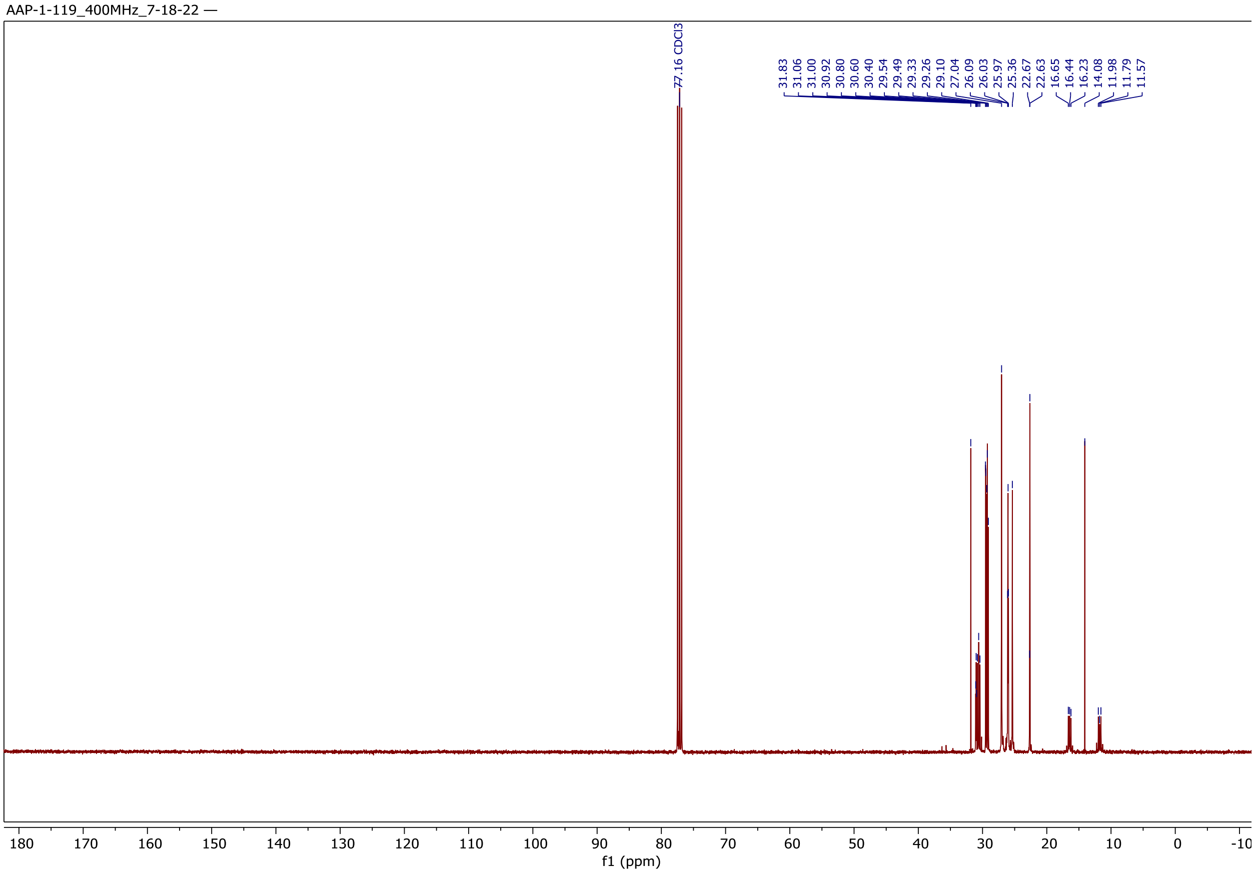

Figure S51. ^{13}C NMR of ^{Cy}**P2P-11,11-Br** in $CDCl_3$

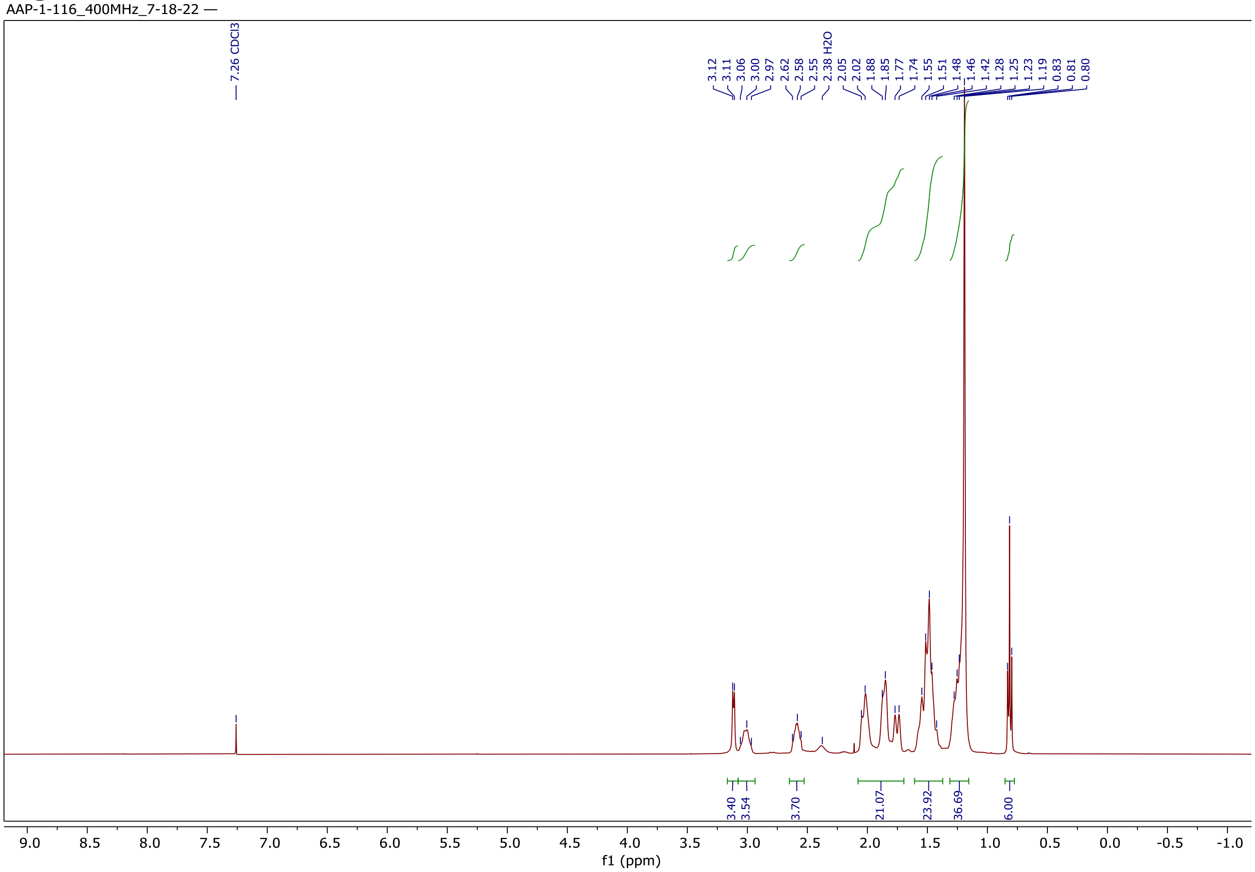

Figure S52. ^{1}H NMR of ^{Cy}**P2P-12,12-Br** in $CDCl_3$

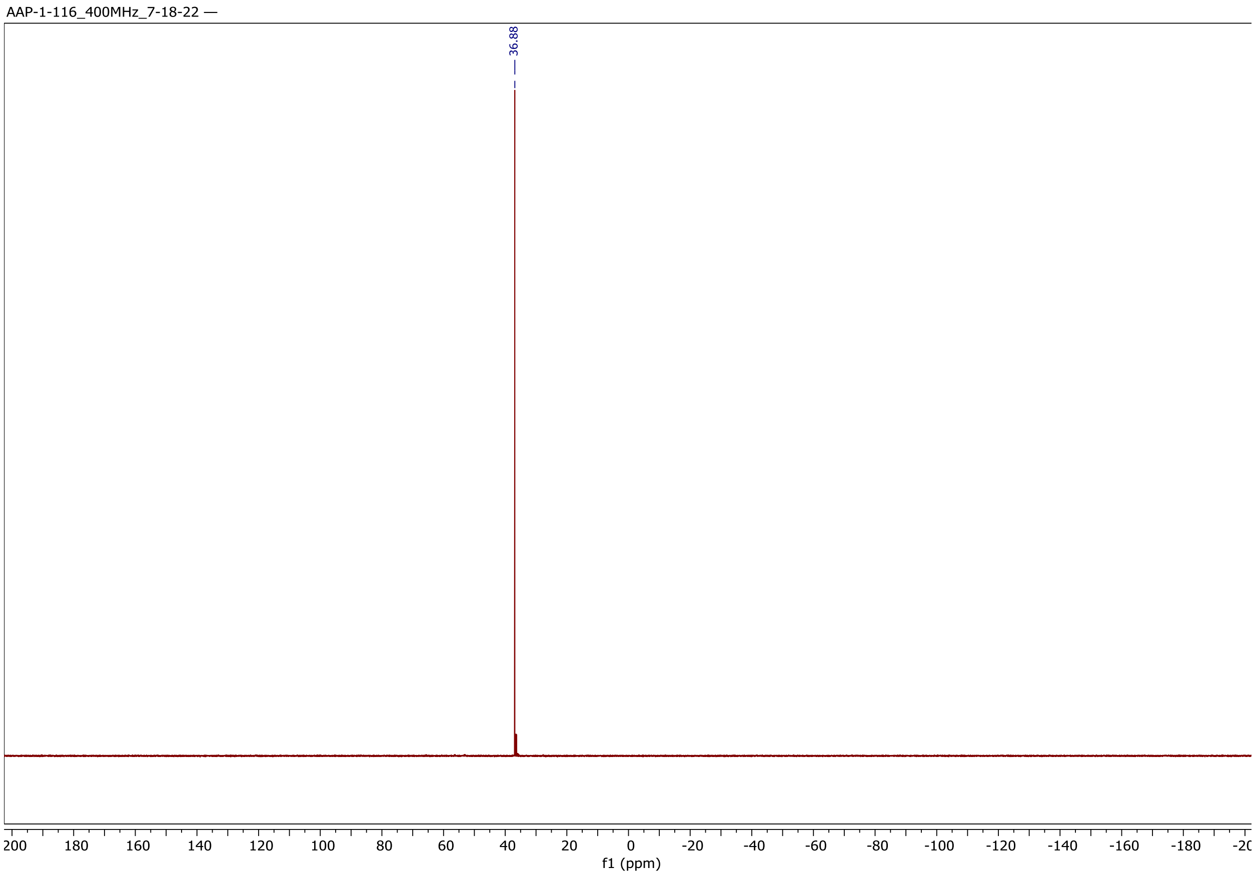

Figure S53. ^{31}P NMR of **^{Cy}P2P-12,12-Br** in $CDCl_3$

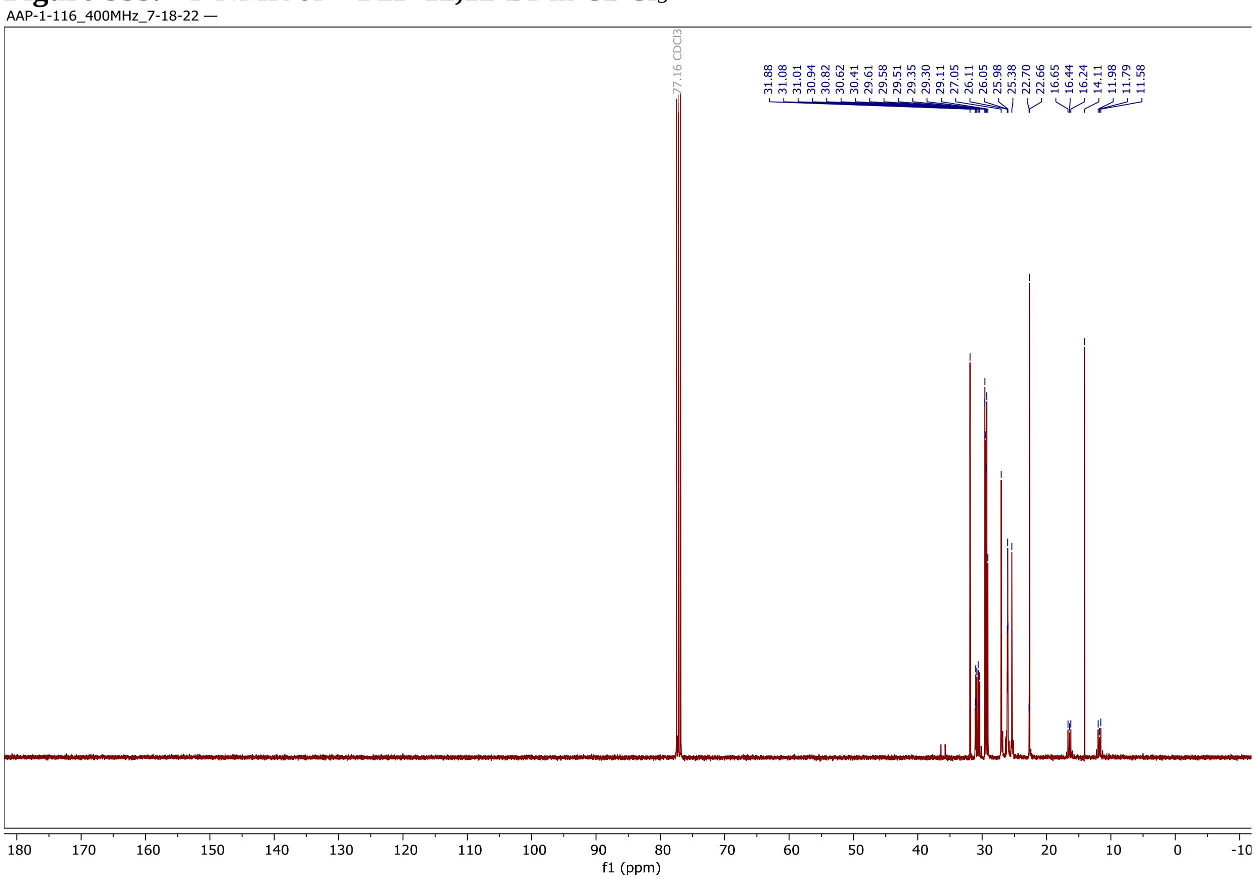

Figure S54. ^{13}C NMR of **^{Cy}P2P-12,12-Br** in $CDCl_3$

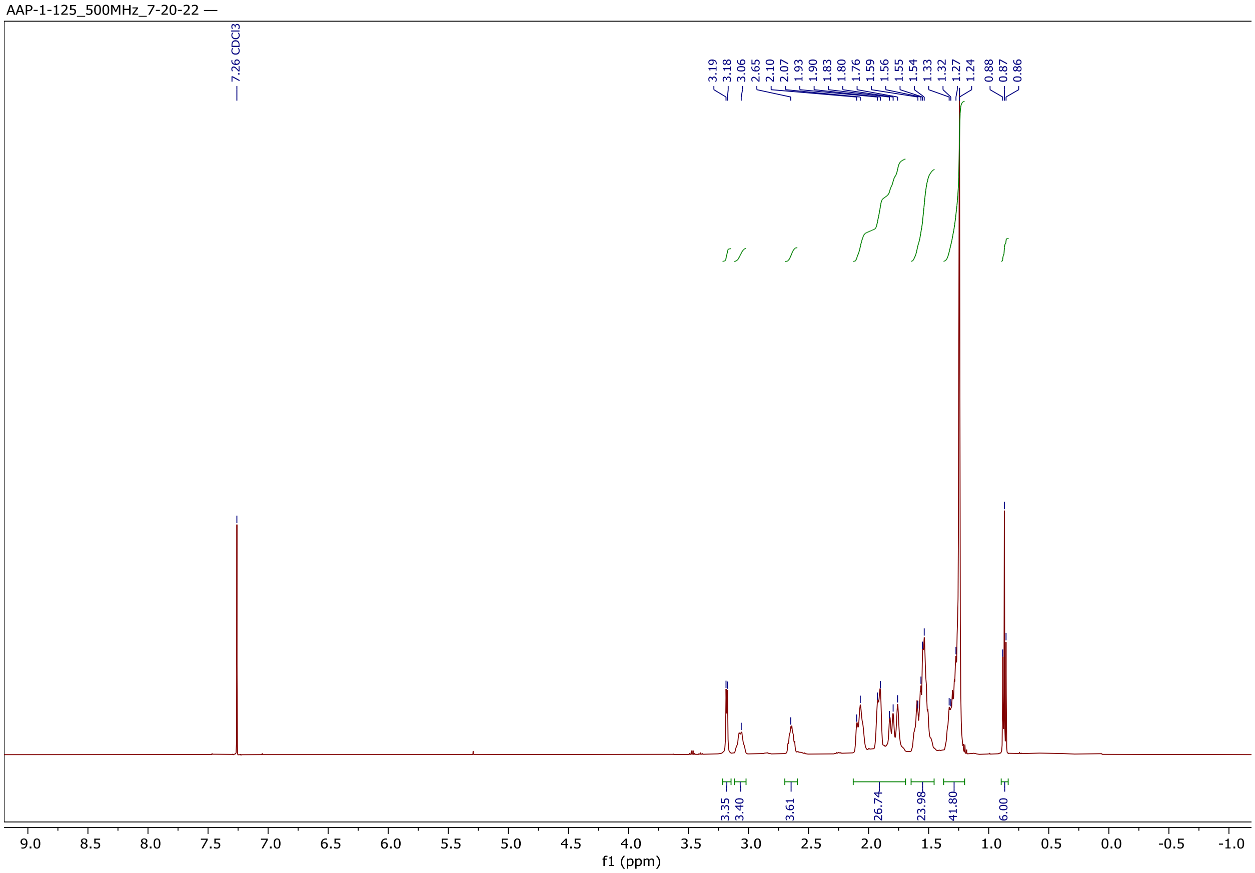

Figure S55. ^{1}H NMR of **^{Cy}P2P-13,13-Br** in $CDCl_3$

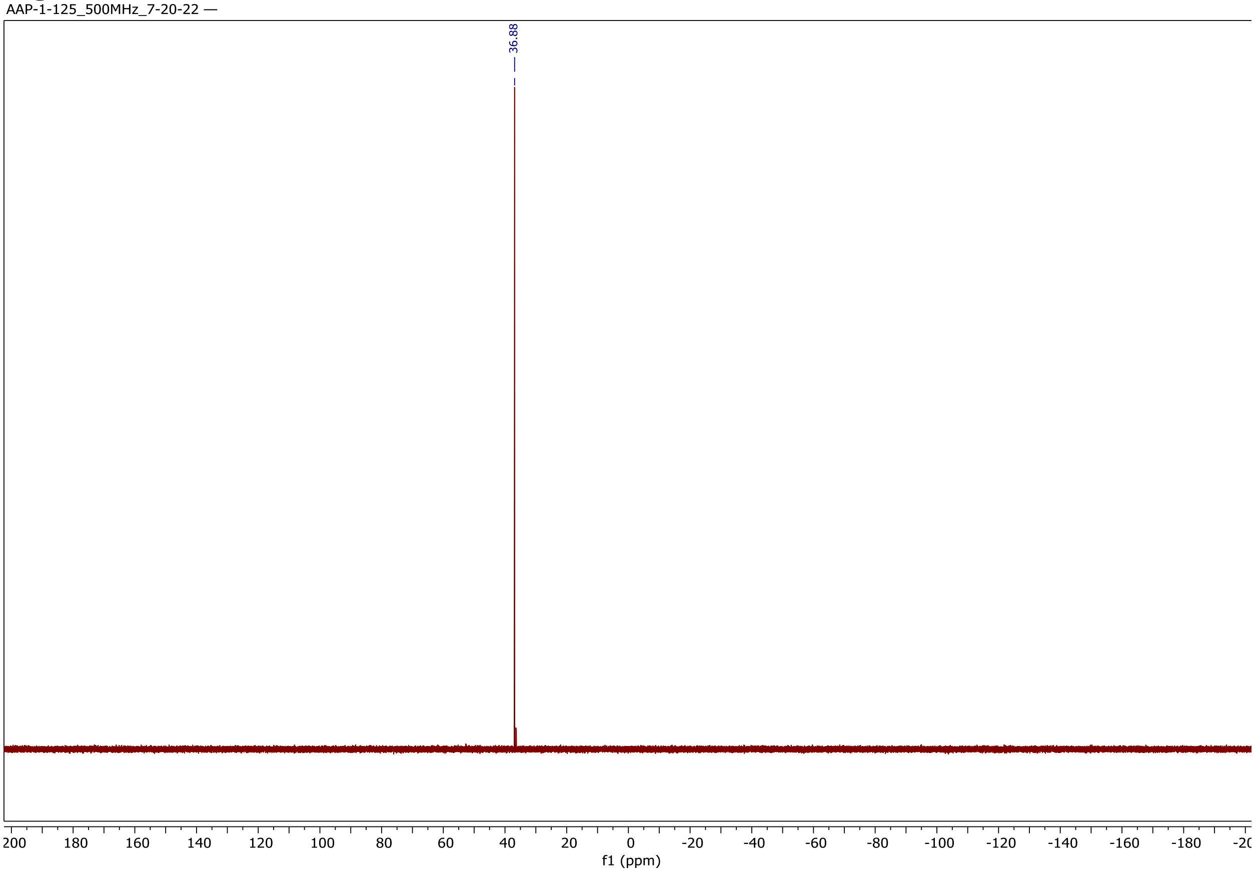

Figure S56. ^{31}P NMR of **^{Cy}P2P-13,13-Br** in $CDCl_3$

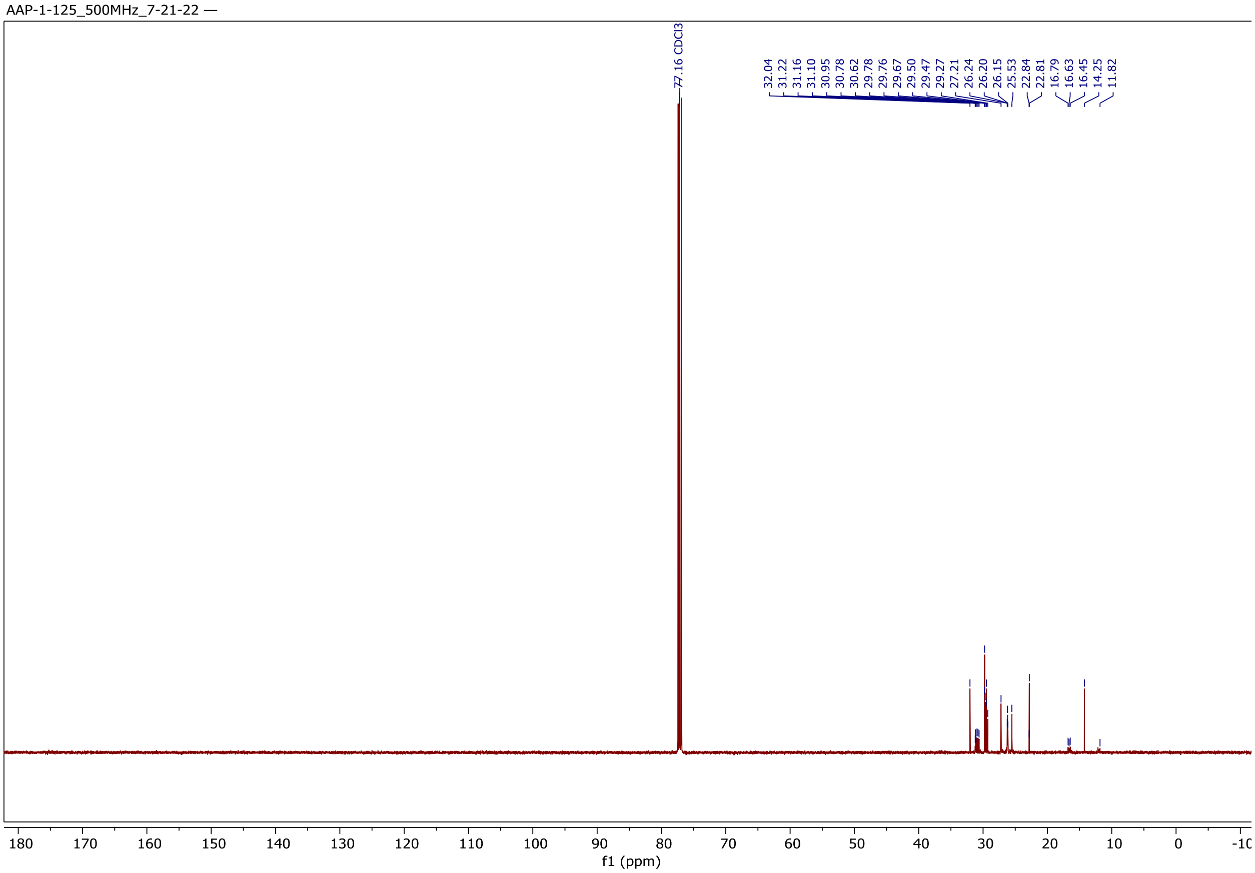

Figure S57. ^{13}C NMR of ^{Cy}**P2P-13,13-Br** in $CDCl_3$

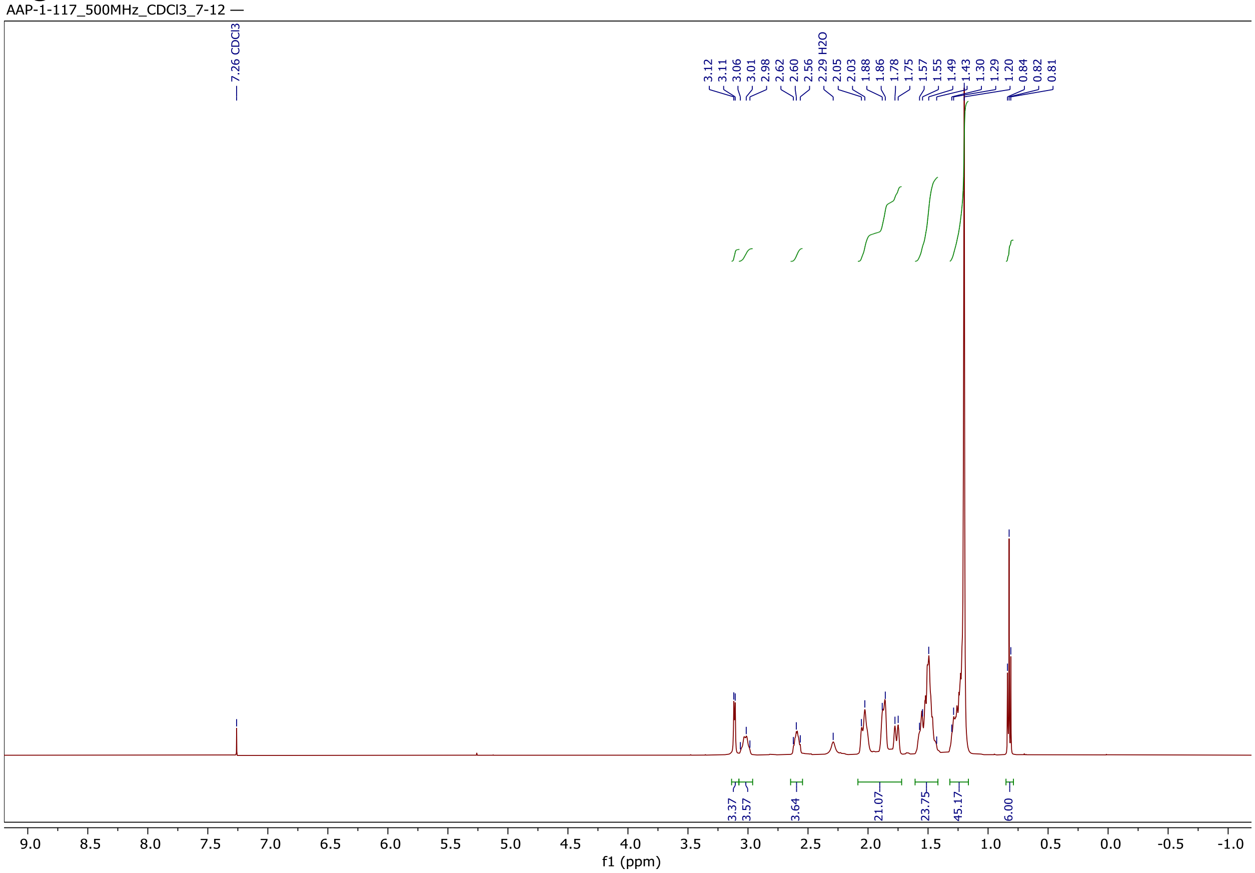

Figure S58. ^{1}H NMR of ^{Cy}**P2P-14,14-Br** in $CDCl_3$

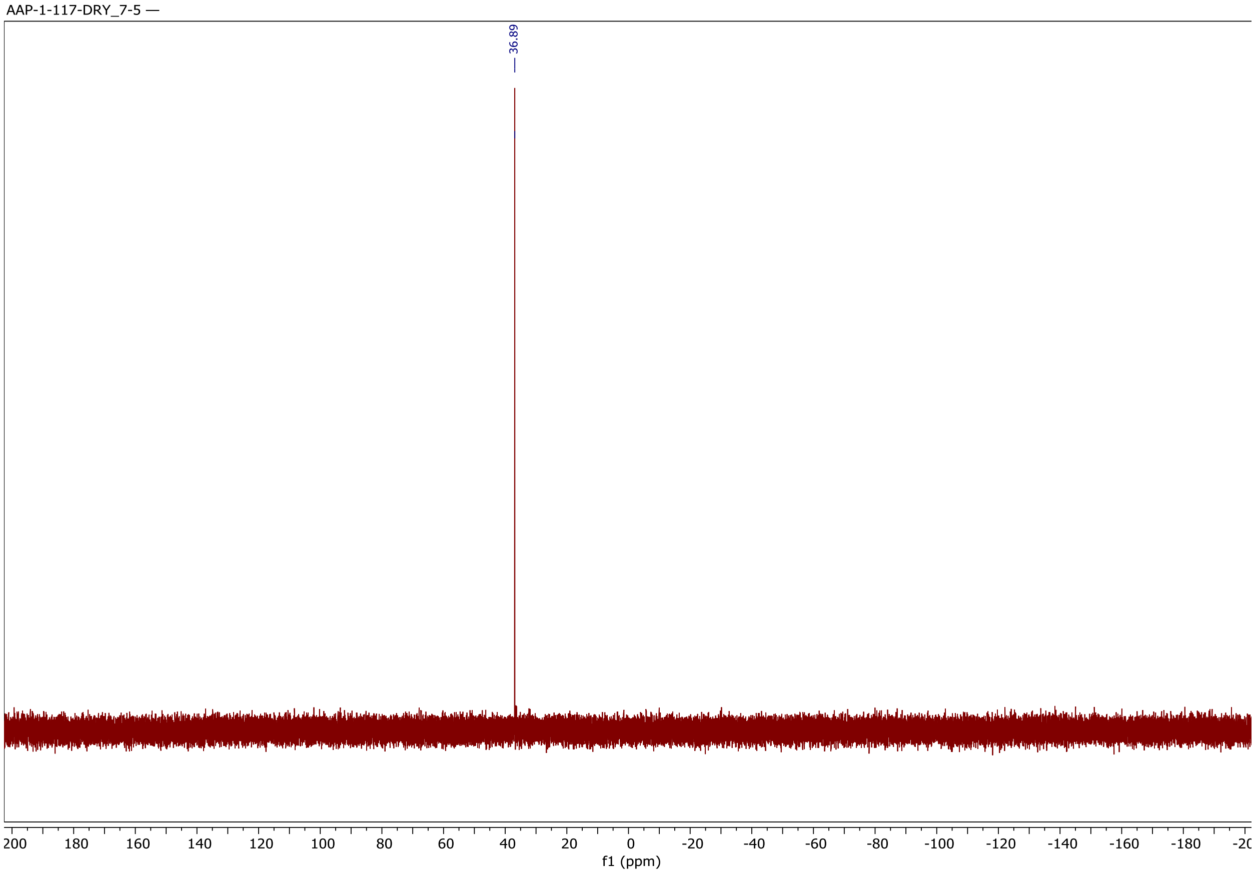

Figure S59. ^{31}P NMR of ^{Cy}**P2P-14,14-Br** in $CDCl_3$

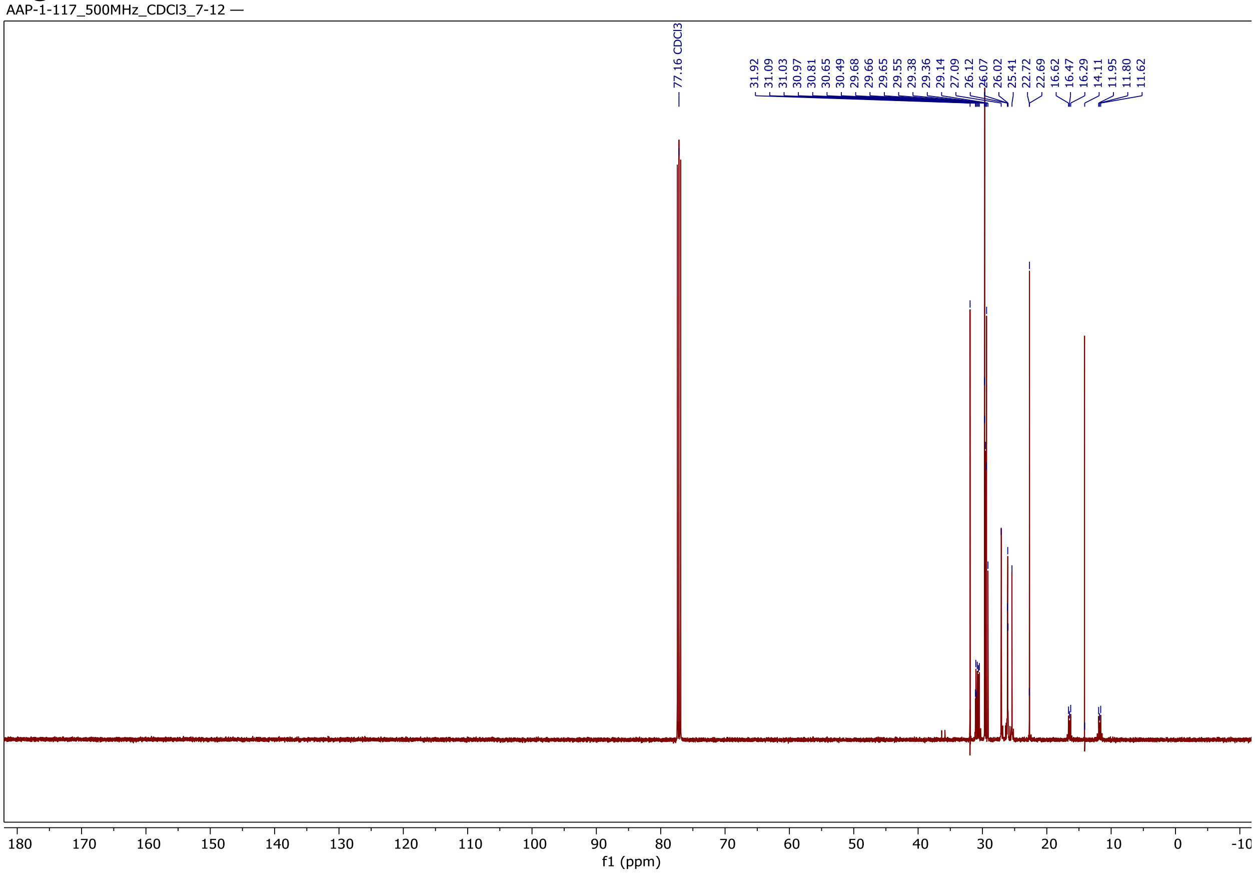

Figure S60. ^{13}C NMR of ^{Cy}**P2P-14,14-Br** in $CDCl_3$

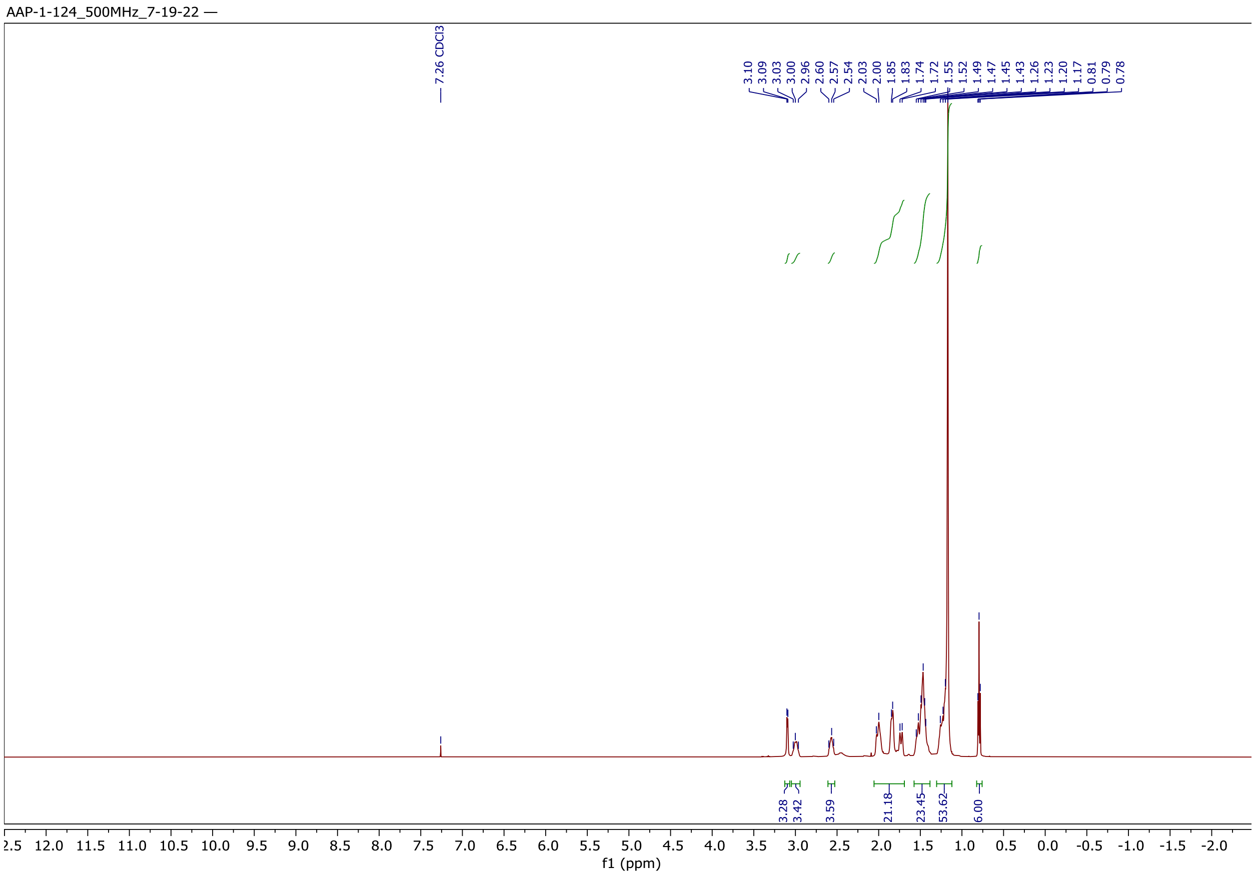

Figure S61. ^{1}H NMR of $^{\mathbf{Cy}}$**P2P-16,16-Br** in $CDCl_3$

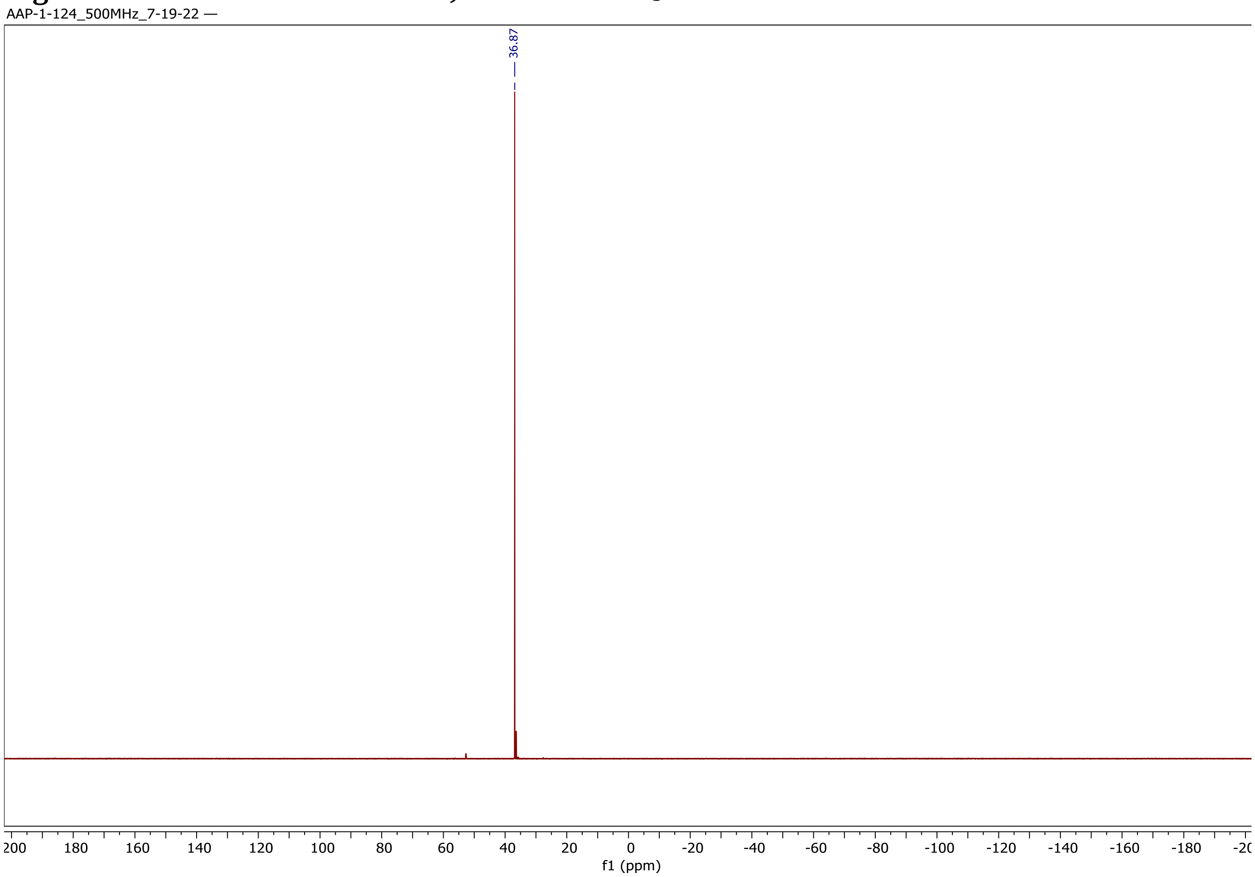

Figure S62. ^{31}P NMR of $^{\mathbf{Cy}}$**P2P-16,16-Br** in $CDCl_3$

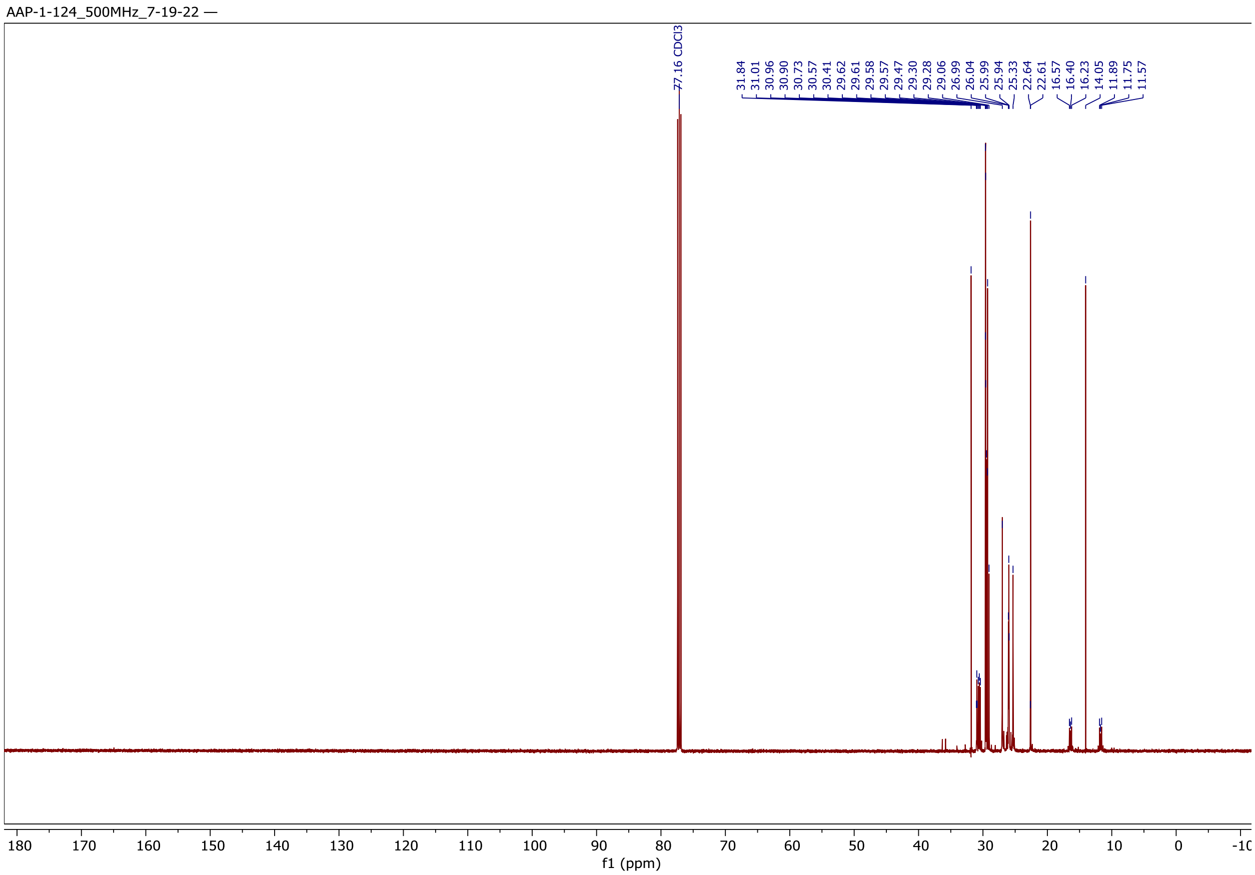

Figure S63. ^{13}C NMR of $^{\mathbf{Cy}}$**P2P-16,16-Br** in $CDCl_3$

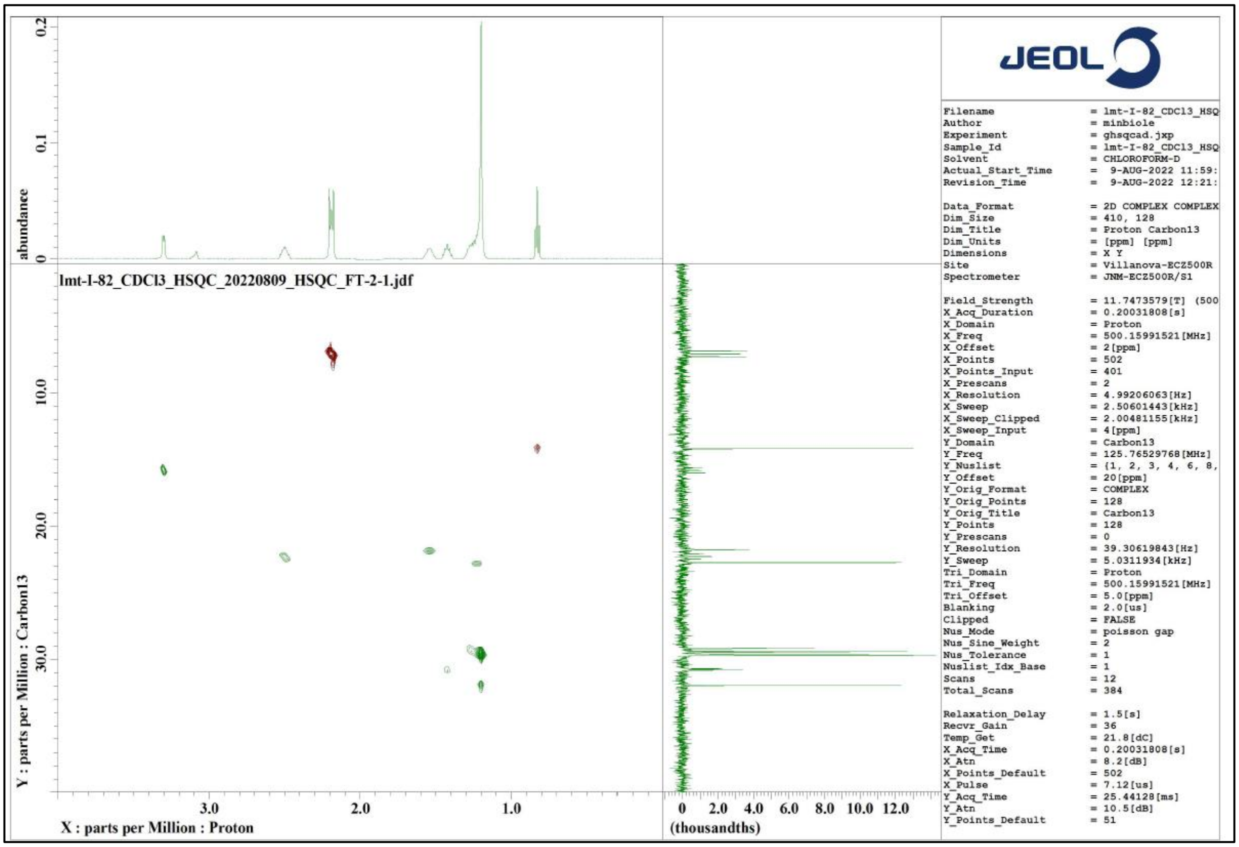

Figure S64. HSQC of **^{Me}P2P-12,12-Br** in $CDCl_3$ correlating the proton and carbon NMR signals. The two spectroscopically unique methyl groups in the molecule are identified as red in the figure, while all methylene signals are noted as green.

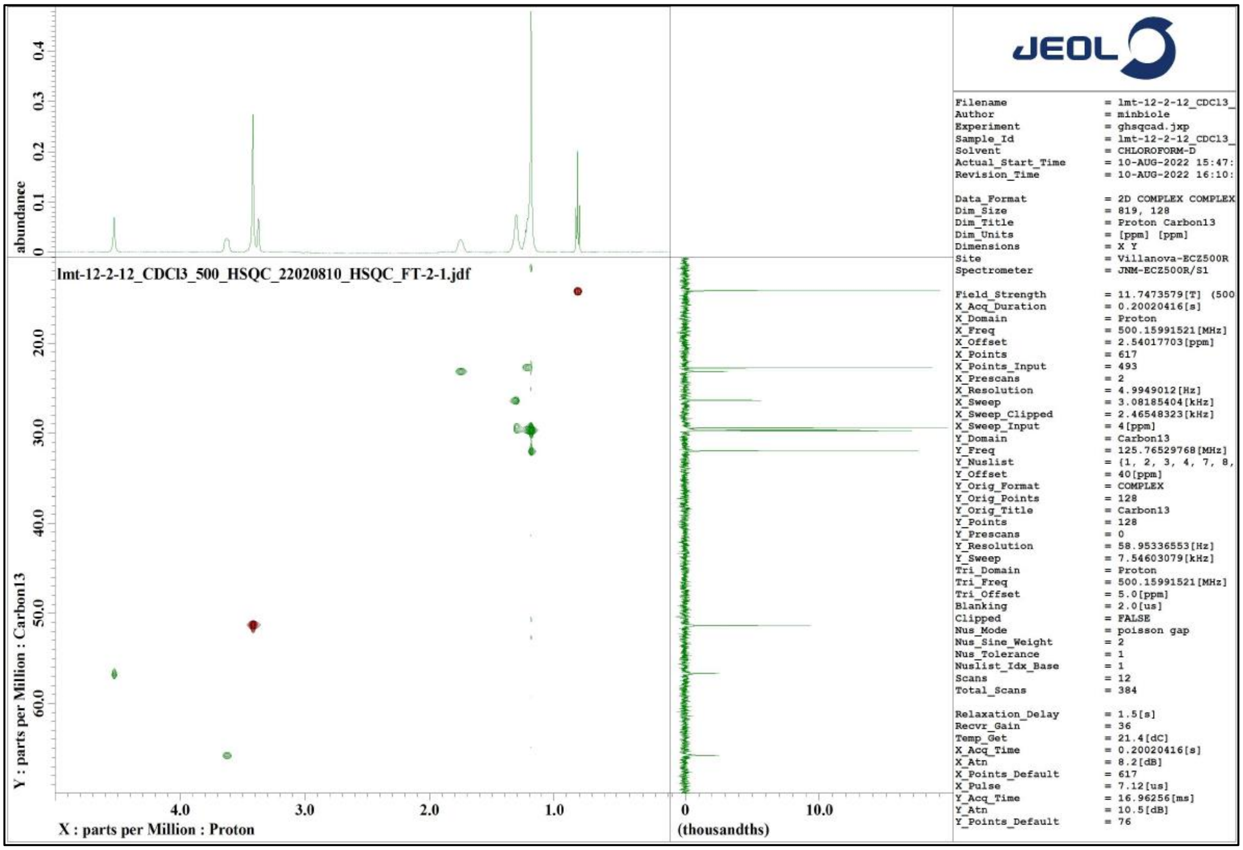

Figure S65. HSQC of **12(2)12** in $CDCl_3$ correlating the proton and carbon NMR signals. The two spectroscopically unique methyl groups in the molecule are identified as red in the figure, while all methylene signals are noted as green.

III. Biofilm Eradication Procedure

Biofilm eradication experiments were performed using a pegged-lid microtiter plate assay to determine the MBEC values for compounds of interest, as previously described.[1,2] Briefly, 125 mL of mid-log phase culture diluted to *ca.* 10^6 CFU/mL in tryptic soy broth (TSB) was added to wells of plat-bottom 96-well plates (Thermo Scientific, 266120) and covered with 96-pegged lid (Thermo Scientific, 445497). Plates were incubated to establish bacterial biofilms after incubation at 37 ºC for 24 hours. The pegged lid was then removed, washed with PBS, and transferred to another 96-well plate containing 2-fold serial dilutions of the test compounds (the "challenge plate"). The total volume in each well was 150 mL, comprising of 75 mL of compound diluted in water/DMSO, with a starting DMSO concentration of 2.5 %, and 75 mL of TSB. Plates were incubated statically at 37 ºC for 24 hours. Next, the pegged lids were transferred to a fresh 96-well plate containing 180 mL of TSB and incubated overnight at 37 ºC. MBEC values were determined as the lowest test concentration that resulted eradicated biofilm (i.e., wells displaying no turbidity in the final plate).

1. Yang, H.; Abouelhassan, Y.; Burch, G.M.; Kallifidas, D.; Huang, G.; Yousaf, H.; Jin,S.; Luesch, H.; Huigens, R.W. A Highly Potent Class of Halogenated Phenazine Antibacterial and Biofilm-Eradicating Agents Accessed Through a Modular Wohl-Aue Synthesis. *Sci. Rep.* **2017,** *7*, 2003. DOI: 10.1038/s41598-017-01045-3
2. Raval, Y. S., Flurin, L., Mohamed, A., Greenwood-Quaintance, K. E., Beyenal, H., & Patel, R. *In Vitro* Activity of Hydrogen Peroxide and Hypochlorous Acid Generated by Electrochemical Scaffolds Against Planktonic and Biofilm Bacteria. *Antimicrob Agents Chemother*. **2021**, *65*, e01966-20. DOI: 10.1128/AAC.01966-20
